# A Multilayer Visibility Graph Framework for Ultrashort-Term Analysis of Multichannel Physiological Signals

**DOI:** 10.3390/s26206668

**Published:** 2026-10-21

**Authors:** Laura Lavezzo, Anna Di Marco, Aleandra Viti, Rebecca Ciacchini, Ciro Conversano, Enzo Pasquale Scilingo, Mimma Nardelli

**Affiliations:** 1 Department of Information Engineering and Research Centre “E. Piaggio”, University of Pisa, 56122 Pisa, Italy; laura.lavezzo@phd.unipi.it (L.L.); anna.dimarco@ing.unipi.it (A.D.M.); enzo.scilingo@unipi.it (E.P.S.); 2 Department of Surgical, Medical, Molecular Pathology and Critical Care Medicine, University of Pisa, 56126 Pisa, Italy; aleandra.viti@ing.unipi.it (A.V.); rebecca.ciacchini@med.unipi.it (R.C.); ciro.conversano@unipi.it (C.C.)

**Keywords:** physiological sensing, wearable sensors, multilayer visibility graph, physiological interactions, digital biomarkers, electrodermal activity (EDA), physiological signal analysis

## Abstract

Recent advances in physiological sensing technologies enable continuous acquisition of multichannel biosignals for digital health applications. Because physiological interactions arise from nonlinear mechanisms, their characterization requires analytical approaches capable of capturing both individual signal dynamics and cross-signal dependencies, often from short recordings. This study proposes a multilayer visibility graph (VG) framework free of phase-space embedding parameters for analyzing physiological interactions from multichannel biosignals. The approach was evaluated in a simulation study based on unidirectionally coupled Rössler oscillators and in two physiological datasets comprising synchronized cardiovascular recordings from healthy adults and wearable electrodermal activity (EDA) recordings acquired during a Mindfulness-Based Stress Reduction (MBSR) intervention. Average Degree (DA) was used to characterize the topological organization of individual and multiplex visibility graphs, while inter-layer mutual information (I) between degree sequences was introduced to quantify cross-layer dependence. In the simulation study, coupling-related changes in the response dynamics were already detectable from single-layer VGs at short window lengths, whereas inter-layer mutual information captured coupling-dependent changes in cross-layer organization. Feature estimates began to stabilize at approximately 480 samples and approached a plateau at longer window lengths. In the cardiovascular dataset, sex-related differences were primarily observed in configurations involving systolic blood pressure: DA differed in single-layer and multichannel representations, while inter-layer mutual information showed significant sex differences, specifically in the interbeat interval-systolic blood pressure duplex. In the EDA dataset, the proposed descriptors identified changes in trainer–trainee physiological organization across the MBSR intervention. These findings indicate that VG-based descriptors provide complementary information on within-signal topology and inter-layer dependence and can be applied to short multichannel physiological recordings without phase-space reconstruction.

## 1. Introduction

Advances in physiological sensing technologies enable the simultaneous acquisition of multiple biosignals, creating new opportunities to investigate interactions among physiological systems and between interacting individuals [1,2,3,4]. Such interactions arise from nonlinear and multiscale regulatory mechanisms and are not fully characterized by analyzing each signal independently [1,5,6].

However, extracting reliable information from multichannel physiological recordings remains challenging when only short observation intervals are available. Many nonlinear approaches require sufficiently long time series to obtain stable estimates and may rely on phase-space reconstruction, for which its results depend on choices such as the embedding dimension and time delay [7,8]. These limitations are particularly relevant in applications involving rapidly evolving physiological states or wearable naturalistic recordings, where temporal localization and limited recording duration constrain the amount of data available for analysis. These methodological challenges arise across different physiological settings. Within individuals, for example, cardiovascular regulation results from continuous interactions among cardiac, vascular, autonomic, respiratory, endocrine, and neural mechanisms, with the relationship between heart period and blood pressure providing a well-established example of coupled physiological dynamics [5,9,10,11,12,13]. Physiological interactions can also occur between individuals [14,15,16,17,18,19,20,21,22,23,24]. Electrodermal activity (EDA), which is closely related to sympathetic nervous system activity [25], has been used to investigate coordinated autonomic responses during social and interpersonal interactions [26,27]. Thus, both multisystem cardiovascular recordings and interpersonal EDA recordings provide relevant settings in which analytical methods must characterize interactions among simultaneously acquired physiological signals rather than isolated time series.

Visibility graph (VG) analysis provides a network-based approach for characterizing nonlinear time-series structure without requiring phase-space reconstruction. Originally introduced by Lacasa et al. [28], the method maps individual observations to graph nodes and connects them according to a geometric visibility criterion, thereby translating temporal organization into network topology. VG-derived measures have been applied to several physiological signals, including cardiovascular, sleep, and neonatal recordings [7,29,30,31,32,33]. Among these measures, Average Degree (DA) provides a compact description of graph connectivity and has shown discriminative ability in physiological applications [7,30,31,32,33]. Nevertheless, two methodological issues remain insufficiently characterized. First, VG descriptors depend on the amount of data used to construct the graph, yet their behavior across short and ultrashort observation windows has not been systematically evaluated. Second, most physiological VG applications have focused on individual time series, whereas multichannel recordings require representations capable of preserving both the topology of individual signals and the dependence among them. Multiplex network representations provide a natural extension of VG analysis to multichannel data by representing each synchronized signal as a separate layer while preserving the temporal correspondence of nodes across layers [34]. This representation also makes it possible to distinguish complementary aspects of multivariate dynamics. Layer-wise and multiplex DA characterize changes in network topology, whereas inter-layer measures can explicitly quantify dependencies across synchronized layers. In particular, inter-layer mutual information (I) between degree sequences provides a measure of the statistical dependence between the topological organization of different layers [34]. Combining these descriptors may therefore provide complementary information on within-signal organization and cross-signal dependence.

In this study, we propose and evaluate a multilayer VG framework free of phase-space embedding parameters for the analysis of short multichannel physiological recordings. The framework is assessed across three complementary settings. First, a simulation study based on unidirectionally coupled Rössler oscillators provides a controlled benchmark in which interaction strength is known and can be systematically varied. Second, synchronized beat-to-beat heart-period and blood-pressure recordings from healthy adults are used to investigate multisystem cardiovascular dynamics. Third, wearable EDA recordings collected from trainers and trainees during a Mindfulness-Based Stress Reduction (MBSR) intervention are used to examine interpersonal physiological organization. Across these settings, we evaluate both DA and I over multiple observation lengths, with the aims of determining their sensitivity to recording duration, distinguishing within-layer topological changes from cross-layer dependence, and assessing the applicability of the framework to short multichannel physiological signals.

## 2. Materials and Methods

### 2.1. Synthetic Validation of Multichannel Interaction Analysis

A synthetic multivariate dataset was generated to evaluate the ability of the proposed framework to detect different levels of interaction under controlled conditions before its application to physiological sensing data.

Synthetic time series were generated from two unidirectionally coupled Rössler oscillators, following the configuration proposed in [35]. The first oscillator acted as the driver and the second as the response system. The dynamics were governed by the following equations:

(1) $$\begin{array}{cc} {\dot{x}}_{1} & =-\omega_{1}x_{2}-x_{3}+\sigma \xi_{1}\left(t\right), \end{array}$$

(2) $$\begin{array}{cc} {\dot{x}}_{2} & =\omega_{1}x_{1}+a_{1}x_{2}+\sigma \xi_{2}\left(t\right), \end{array}$$

(3) $$\begin{array}{cc} {\dot{x}}_{3} & =b+x_{3}\left(x_{1}-c_{1}\right)+\sigma \xi_{3}\left(t\right), \end{array}$$

(4) $$\begin{array}{cc} {\dot{y}}_{1} & =-\omega_{2}y_{2}-y_{3}+\kappa \left(x_{1}-y_{1}\right)+\sigma \xi_{4}\left(t\right), \end{array}$$

(5) $$\begin{array}{cc} {\dot{y}}_{2} & =\omega_{2}y_{1}+a_{2}y_{2}+\sigma \xi_{5}\left(t\right), \end{array}$$

(6) $$\begin{array}{cc} {\dot{y}}_{3} & =b+y_{3}\left(y_{1}-c_{1}\right)+\sigma \xi_{6}\left(t\right). \end{array}$$

Parameters were fixed as

(7) $$\omega_{1}=0.5,\;\omega_{2}=2.515,\;a_{1}=0.15,\;a_{2}=0.72,\;b=0.2,\;c_{1}=10.$$

The scalar κ denotes the coupling strength, varied as

**Figure 1:**
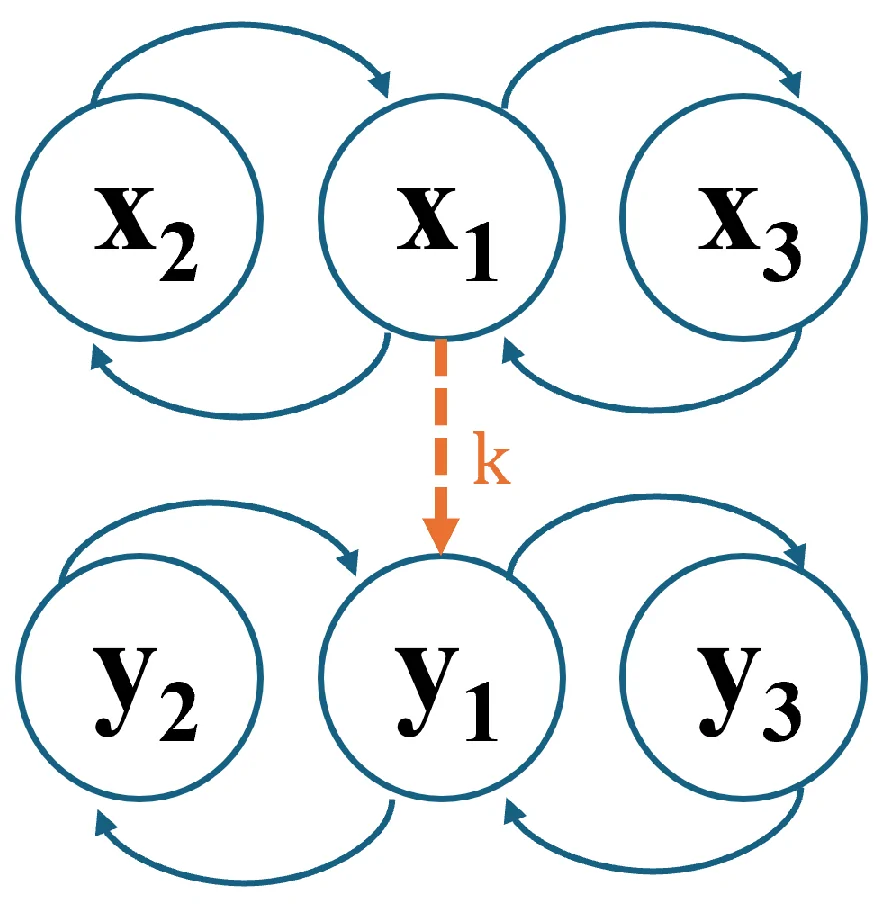
Interaction graph for coupled Rössler systems. This diagram shows the connections between two maps: map X ((**top row**) nodes $x_{1},x_{2}$, and $x_{3}$) and map Y ((**bottom row**) nodes $y_{1},y_{2}$, and $y_{3}$). The blue arrows show constant internal interactions within each system. The parameterized connection across maps is shown by the orange dashed arrow (*k*). The graph is adapted [35].

(8) κ∈{0,0.35,0.7,1.1},

and σ=0.03 [1] is the amplitude of Gaussian white noise, with $\xi_{i}\left(t\right)∼N\left(0,1\right)$. The system was numerically integrated using an adaptive Runge–Kutta scheme (MATLAB 2021b^®^, *ode45*), with default relative ($\mathrm{RelTol}=10^{-3}$) and absolute ($\mathrm{AbsTol}=10^{-6}$) tolerances over the interval t∈[0,2753] s, and sampled at $f_{s}=4$ Hz (step size Δt=0.25s). For each coupling value, 30 independent realizations were generated, initializing the seed at 42. The first 1000 samples were discarded to remove transient dynamics before subsequent analyses. A schematic representation of the relationship across time series can be seen in Figure 1.

### 2.2. Multichannel Cardiovascular Sensing Dataset (HYPOL)

The publicly available HYPOL (Healthy Young POLes) dataset [36] collects beat-to-beat cardiovascular recordings from 276 healthy young adults of Polish descent (147 women and 129 men, aged 19–30 years, 25.75 ± 2.59), collected at the Department of Cardiology–Intensive Therapy, Poznan University of Medical Sciences. Ethical approval was obtained from the local bioethics committee at the Poznan University of Medical Sciences. Synchronized electrocardiographic (ECG) and continuous non-invasive finger arterial blood pressure were recorded during supine resting state using non-invasive sensors (bipolar chest-lead ECG and Portapres 2 or Finapres Nova, FMS, Netherlands) providing multimodal cardiovascular sensing data. The database includes interbeat (RR) intervals derived from ECG recordings, as well as systolic (SBP), diastolic (DBP), and mean arterial pressure time series, together with basic clinical characteristics. Further information on the HYPOL dataset can be found in [36]. The recording sessions lasted up to 45 min. For the present study, the first 15 min of recordings without visible long artifacts were selected for each subject. According to the original database preprocessing procedure [36], outliers were corrected using piecewise cubic interpolation. Beats flagged as non-sinus origin and values outside physiological ranges (RR outside 500–1500 ms; blood pressure outside 10–270 mmHg) were identified as outliers.Processing was performed using MATLAB 2021b^®^. The resulting RR, SBP, and DBP time series were used for subsequent analyses.

### 2.3. Wearable Electrodermal Sensing During a Mindfulness-Based Stress Reduction Intervention

To complement the validation on publicly available cardiovascular recordings, we prospectively acquired a wearable physiological sensing dataset recording EDA signals from infertile couples undergoing an MBSR intervention [37] at the University Hospital of Pisa. The experimental protocol enabled synchronized acquisition of physiological signals from interacting individuals during a controlled therapeutic intervention. This study was approved by the local Ethics Committee (ID 27213, Comitato Etico Regionale della Toscana, Sezione Area Vasta Nord Ovest), and all participants provided written informed consent prior to participation. A total of 12 couples diagnosed with unexplained infertility were enrolled across three consecutive MBSR programs while awaiting assisted reproductive technology. Each program followed the standard MBSR protocol consisting of eight weekly sessions delivered by certified instructors. For the present study, only participants who regularly attended the intervention and completed both the first and final sessions were included, resulting in a final dataset of 15 individuals (36.77 ± 4.33 years, 8 female). Wearable physiological sensing was performed using synchronized Shimmer3 GSR+ devices (Shimmer Research Ltd., Dublin, Ireland) placed on participants and trainer. EDA signals were continuously recorded with a sampling rate of 125 Hz during two phases in each MBSR session: the final meditation task (9 min long) and a preceding 3 min resting state condition, during which participants were instructed to relax with their eyes closed. The EDA electrodes were placed on the index and middle fingers of the non-dominant hand. The software Shimmer^®^ ConsensysPRO v. 1.6.0 was used for data recording, monitoring, sensor synchronization, and event making.

The EDA signals were preprocessed and analyzed using MATLAB 2024b^®^. EDA raw signals were resampled at 50 Hz and processed using the cvxEDA algorithm [38] (τ0 = 2, τ1 = 0.7, delta-knot = 10, $\alpha =8\times 10^{-3}$, $\gamma =1\times 10^{-2}$, solver = quadprog, baseline correction = linear; toolbox version 1.0) to remove noise and motion artifacts and to extract sudomotor nerve activity (SMNA), reflecting autonomic control of sweat gland activity in response to various environmental and individual factors, e.g., emotional arousal and thermoregulation [39]. Series were then downsampled at 4 Hz to allow comparability of window length. SMNA is modeled as a sparse, non-negative impulse train, consisting of a sequence of discrete neural bursts.

**Figure 2:**
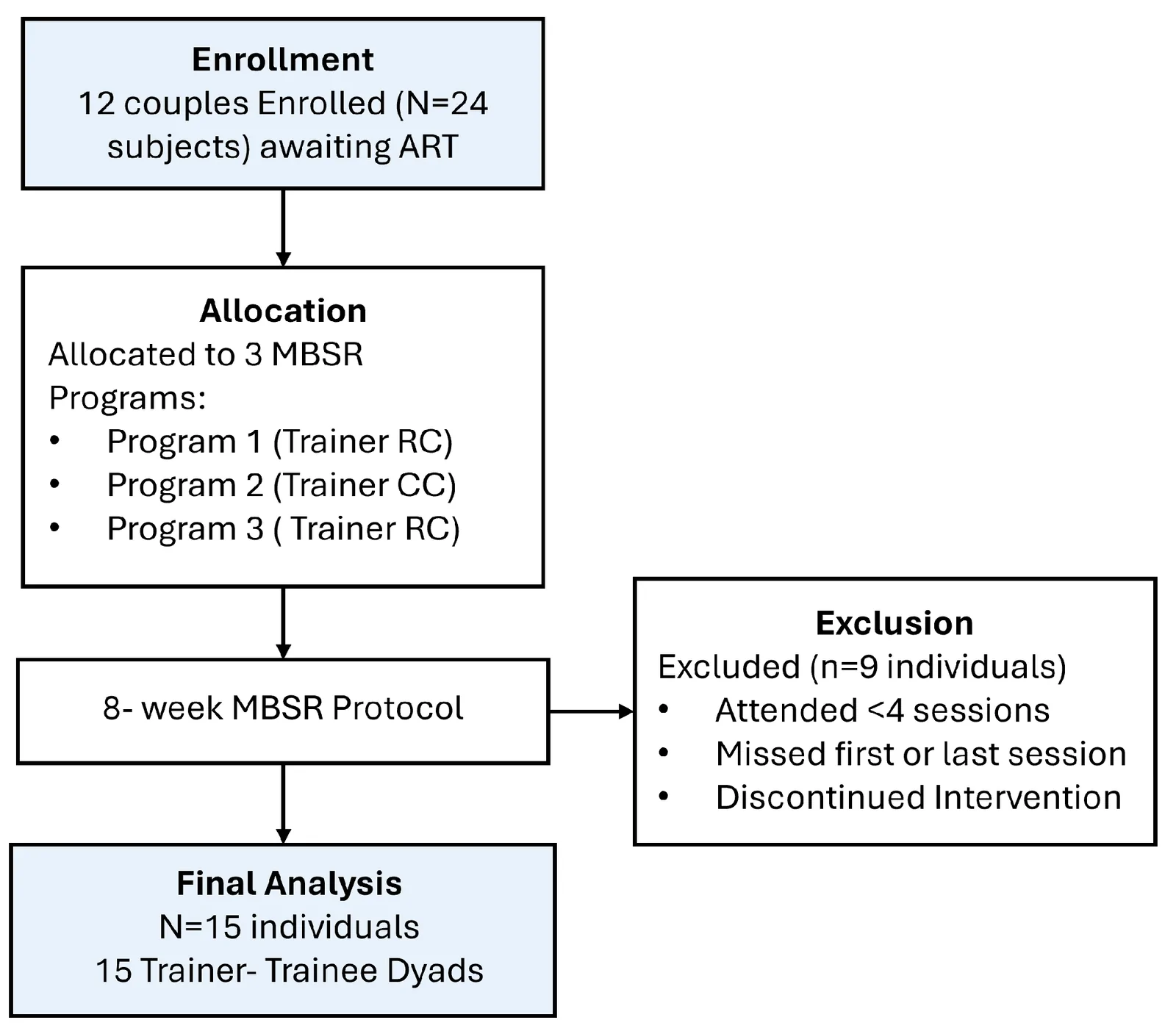
Participant flow diagram. Twelve couples (24 individuals) were enrolled across three MBSR programs. Nine individuals were excluded from the analysis because they attended fewer than four sessions, missed the first or final assessment session, or discontinued the intervention. The final sample comprised 15 trainees, each paired with the instructor of their MBSR group, yielding 15 trainer–trainee dyads.

In this study, we used the VG approach to quantify the interpersonal physiological interaction between each trainer–trainee pair monitored in the three MBSR groups, using SMNA time series from a total of 15 individuals who participated in both the first and final MBSR sessions (see Figure 2 for more details).

### 2.4. Visibility Graph (VG) Analysis

The VG computational framework was applied to the three physiological sensing scenarios described above by transforming synchronized multichannel biosignals into multiplex visibility graphs. This method transforms time series (the physiological recordings in the case of this study) into complex network representations, allowing the characterization of their dynamical properties through network-based metrics [28]. In this approach, each data point is treated as a node, and connections between nodes are established according to a geometric visibility criterion, whereby two points are linked if a direct “line of sight” exists between them. This mapping preserves key structural properties of the original signal, allowing features such as periodicity, fractality, and nonlinearity to be characterized through graph topology. Although originally introduced for univariate signals [28], VG analysis can naturally be extended to multichannel physiological recordings through multiplex representations [34], where each sensed physiological variable is mapped onto an independent network layer while preserving temporal alignment across nodes. Within the resulting networks, node-level properties, such as the degree (number of connections of a node), can be used to obtain global descriptors. In the proposed framework, the Average Degree (DA) was adopted as the primary digital biomarker because it provides a compact description of the organization of multichannel physiological dynamics. In contrast, interlayer mutual information (I) has been presented as a multilayer feature of information flow among degrees of freedom and synchronization [34]. VG-based measures have demonstrated strong discriminative power in physiological applications, particularly in cardiovascular time series analysis, where they have been successfully used to characterize sleep stages and pathological conditions (e.g., [7,31,33]).

**Figure 3:**
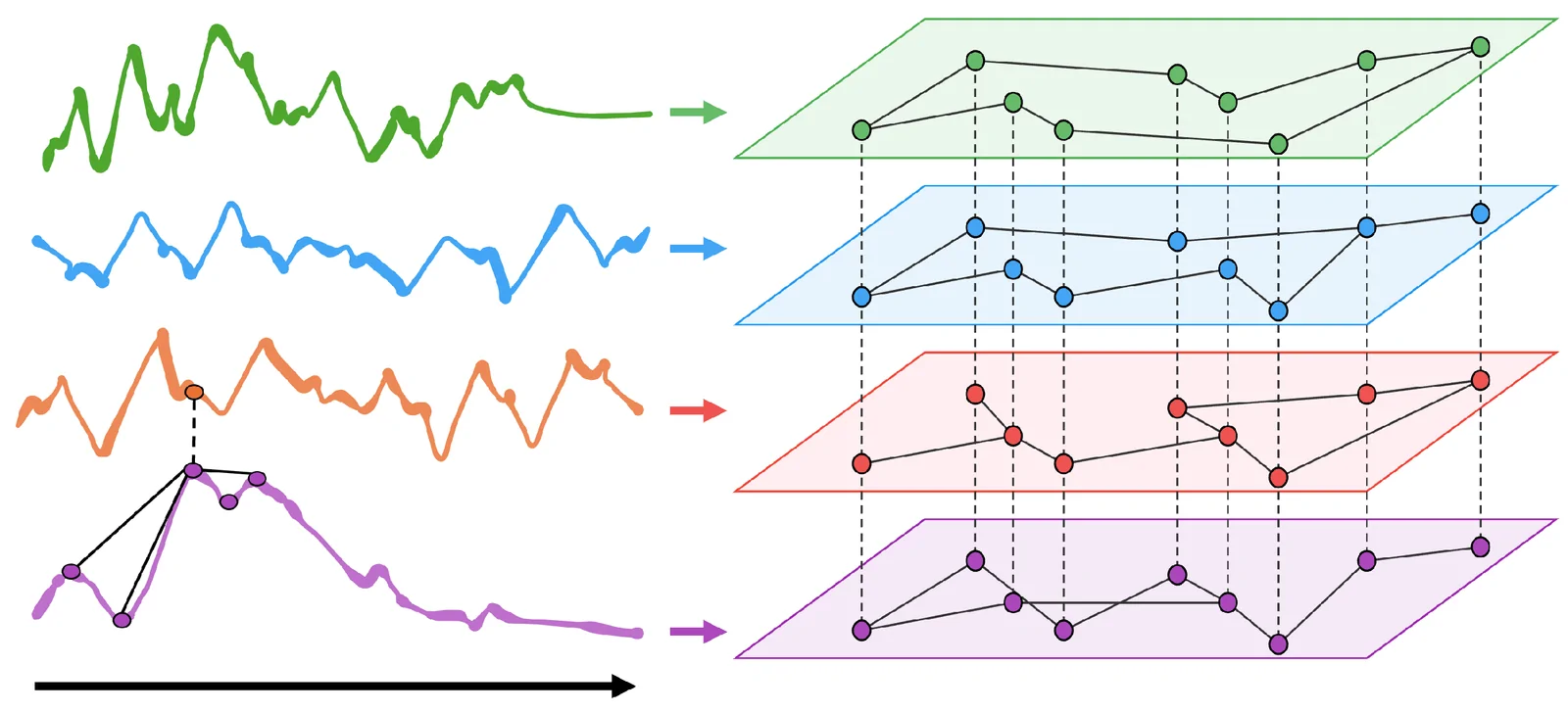
Schematic overview of the multiplex visibility graph construction. Synchronized time series (**left**) are individually transformed into single-layer visibility graphs (**right**). Intra-layer connections (solid black lines) are established based on the geometric visibility criterion, while vertical dashed lines represent inter-layer identity links connecting time-matched nodes across different layers. Partially adapted from [34].

The analysis pipeline is schematically illustrated in Figure 3. Synchronized multichannel recordings were first segmented according to the window lengths described in the Windowing Length Assessment Section. Within each window, every signal was independently mapped into a natural visibility graph, with samples represented as nodes and visibility relationships defining intra-layer edges. The resulting single-layer graphs were subsequently assembled into a multiplex representation in which temporally corresponding nodes were connected across layers through identity links. Two complementary network descriptors were then extracted: Average Degree (DA), describing graph topology, and inter-layer mutual information ($I_{\alpha ,\beta }$) between intra-layer degree sequences, describing statistical dependence across layers. When multiple windows were available, descriptor values were aggregated within participant/realization before statistical analysis, as specified in the Windowing Length Assessment Section. For each individual time series of length *N*, the single-layer VG was constructed according to the following visibility criterion:

(9) $$s_{k}<s_{i}+\frac{s_{j}-s_{i}}{t_{j}-t_{i}}\left(t_{k}-t_{i}\right),\;\forall t_{k}\in \left(t_{i},t_{j}\right).$$

where $t_{i}$ refers to the time-point of sample i, and $s_{i}$ is the corresponding data sample.

This create a single-layer VG, represented by an N×N adjacency matrix. From the VG, the Average Degree (DA) was defined as follows:

(10) $$DA=\frac{1}{N}\sum_{i=1}^{N}k_{i},$$

where $k_{i}$ is the degree of node *i*, and *N* is the number of nodes, corresponding to the series length.

The single-layer graph has been extended to an *M*-layer multiplex network constructed from *M* time series of length *N*. The multiplex is defined over the node set V={(i,α)∣i∈{1,…,N},α∈{1,…,M}}, yielding a total node cardinality of |V|=M·N. The overall structure is represented by a supra-adjacency matrix A structured as an M×M block matrix:

(11) $$A_{\alpha \beta }=\left\{\begin{array}{cc} A^{\left[\alpha \right]} & \mathrm{if}\;\alpha =\beta ,\\ I_{N} & \mathrm{if}\;\alpha \neq \beta , \end{array}\right.$$

where $A^{\left[\alpha \right]}$ is the N×N adjacency matrix of the single-layer VG at layer α, and $I_{N}$ is the identity matrix establishing diagonal inter-layer links between time-matching nodes across layers. The degree d(i,α) of node *i* at layer α integrates both intra-layer visibility connections and inter-layer identity edges:

(12) $$d\left(i,\alpha \right)=k_{i}^{\left[\alpha \right]}+\left(M-1\right)=\sum_{j=1}^{N}A_{ij}^{\left[\alpha \right]}+\left(M-1\right),$$

where $k_{i}^{\left[\alpha \right]}$ denotes the intra-layer degree of node *i* derived from the visibility graph on layer α, and M−1 accounts for the inter-layer identity couplings to the corresponding nodes in the remaining layers. Therefore, the global Average Degree is computed as

(13) $${\mathrm{DA}}_{\mathrm{multiplex}}=\frac{1}{M\cdot N}\sum_{\alpha =1}^{M}\sum_{i=1}^{N}d\left(i,\alpha \right)=\left(\frac{1}{M}\sum_{\alpha =1}^{M}{\mathrm{DA}}^{\left[\alpha \right]}\right)+\left(M-1\right)$$

A graphical representation of the multiplex construction can be seen in Figure 3.

To complement the global structural characterization provided by the Average Degree (DA), an inter-layer degree mutual information ($I_{\alpha ,\beta }$) metric was introduced as a local feature [34]. For a pair of layers α and β, $I_{\alpha ,\beta }$ measures the information shared between time-matching nodes degree series:

(14) $$I_{\alpha ,\beta }=\sum_{k^{\left[\alpha \right]}}\sum_{k^{\left[\beta \right]}}P\left(k^{\left[\alpha \right]},k^{\left[\beta \right]}\right)\ln\left(\frac{P(k^{\left[\alpha \right]},k^{\left[\beta \right]})}{P\left(k^{\left[\alpha \right]}\right)\;P\left(k^{\left[\beta \right]}\right)}\right),$$

where $P(k^{\left[\alpha \right]},k^{\left[\beta \right]})$ is the joint probability density function of the node degrees across layers α and β, and $P(k^{\left[\alpha \right]})$ and $P(k^{\left[\beta \right]})$ are the respective marginal distributions. In this work, the joint and marginal probabilities were estimated non-parametrically using a multivariate Gaussian Kernel Density Estimator [40]. When M>2, the overall inter-layer mutual information $\bar{I}$ is computed as the average across all unique pairwise layer combinations. Henceforth, *I* will be referred to the two-layer configuration, while the multilayer version will be referred as $\bar{I}$.

The following configurations were tested on each dataset.

Synthetic Validation (Rössler System):

- Single Layer: Individual VGs constructed separately for each of the six simulated time series ($x_{1},x_{2},x_{3},y_{1},y_{2},y_{3}$).
- Duplex: Pairwise multiplex network constructed for the directly coupled $x_{1}$–$y_{1}$ subsystem to evaluate localized inter-layer interactions.
- Multiplex: A complete 6-layer multiplex network embedding all six time series ($x_{1},x_{2},x_{3},y_{1},y_{2},y_{3}$).

Multisystem Cardiovascular Sensing (HYPOL Dataset):

- Single Layer: Individual VGs constructed for RR, SBP, and DBP time series separately.
- Duplex: Pairwise multiplex networks constructed for RR–SBP, RR–DBP, and SBP–DBP combinations.
- Multiplex: A 3-layer multiplex network embedding RR, SBP, and DBP simultaneously.

Wearable Interpersonal Sensing:

- Duplex: Pairwise multiplex networks constructed for the coupled trainer–trainee.

#### Windowing Length Assessment

Synthetic, public data, and ad hoc were used to assess feature stability across recording lengths. In both application, windows were extracted per subject or realization starting from the recording onset. Windows were selected as follows:

- Synthetic Validation: Eleven window lengths (N∈{30,60,120,240,480,600,720,900,1200,2400,3600} samples) were extracted post-transient across 4 coupling levels (30 realizations each, for a total 120 segments per each window length). A single window was extracted for each N. DA was computed for all single-layer, duplex $x_{1}$–$y_{1}$, and 6-layer configurations, while $\left(\bar{I}\right)$ was calculated specifically for the $x_{1}$–$y_{1}$ duplex.
- Multisystem Cardiovascular Sensing (HYPOL): Fifteen window lengths were tested (N∈{60,120,180,240,300,360,420,480,540,600,660,720,780,840,900} s). For each length, a single window was extracted per subject (n=147 women, n=129 men). DA and *I* were calculated across all single-layer, pairwise duplex, and 3-layer configurations.
- Wearable EDA Signals During the MBSR Intervention: Non-overlapping ultrashort-term wearable recordings of 60 and 180 s were used for each phase of resting state and meditation; then, we computed the mean of DA and *I* values computed across the windows.

### 2.5. Statistical Analysis

Statistical analyses were designed to evaluate the robustness of the extracted network features (DA and *I*) across sensing scenarios, recording lengths, and physiological conditions.

For the synthetic validation (Rössler system), differences among four coupling strengths were assessed across realizations for each window length using the Kruskal–Wallis omnibus test. Global effect sizes were estimated using epsilon squared ($\epsilon^{2}$) accompanied by 95% bootstrap confidence intervals (CIs, 1000 repetitions). Significant main effects were followed by unpaired Mann–Whitney U tests for post hoc pairwise comparisons. Pairwise effect sizes were quantified using the rank correlation effect size (*r*) along with their 95% bootstrap CIs.

For the multichannel cardiovascular sensing dataset (HYPOL), sex differences (males vs. females) in DA and *I* were evaluated across all network configurations and window lengths using the Mann–Whitney U test. Effect sizes were quantified using the rank correlation (*r*) along with 95% bootstrap CIs.

For the wearable electrodermal sensing dataset, within-couple (trainer–trainee) differences between sessions (first vs. last) and conditions (rest vs. meditation) were evaluated using paired Wilcoxon signed-rank tests across configurations and window lengths.

Correction was performed separately within prespecified families of related hypotheses. For the synthetic dataset, each family comprised all tests performed across window lengths for a given network configuration, descriptor, and time-series combination; omnibus and post hoc tests were corrected separately. For the HYPOL dataset, each family comprised the sex comparisons across all window lengths for a given network configuration, time-series combination, and descriptor. For the EDA dataset, each family comprised the four planned within-dyad comparisons (first rest vs. first meditation, last rest vs. last meditation, first vs. last rest, and first vs. last meditation) for a given descriptor and window length. Benjamini–Hochberg adjusted *p*-values are rank-dependent and therefore are not obtained by multiplying all raw *p*-values by a constant factor; consequently, the ratio between raw and adjusted *p*-values may differ across comparisons. Statistical analyses were performed in R (v. 4.4.2).

#### 2.5.1. EDA Sensing Dataset Surrogates Statistical Analysis

To assess whether the physiological organization observed in real trainer–trainee dyads differed from that obtained from dyad-unspecific pairings, we conducted a surrogate analysis using pseudo-dyads composed of individuals who had not interacted. Following established protocols in dyadic physiology [41,42], pseudo-dyads were constructed by pairing trainees with trainers from independent experimental sessions in which they had never interacted. For example, the trainer from session 1 was paired with trainees from sessions 2 and 3.

The surrogate’s null reference distribution was created by window shuffling across experimental conditions, sessions, and time points, including both homologous and non-homologous pairings. Specifically, the surrogate pairings included matched and mismatched conditions combinations (for example, meditation–meditation, rest–rest, and meditation–rest), as well matched and mismatched time points (e.g., first–first, last–last, first–last, and last–first).

For each descriptor, the empirical distribution of real dyadic interactions was then statistically evaluated against the surrogate’s null reference distribution using two-sample one-tailed Kolmogorov–Smirnov (KS) tests and Mann–Whitney U tests. The KS test statistic served as a non-parametric effect size metric. The Mann–Whitney U test was used to assess stochastic dominance between the real and surrogate distributions, namely whether an observation drawn from the real distribution was more likely to exceed an observation drawn from the surrogate distribution. Both KS and Mann–Whitney U FDR correction (BH) were applied. As the real dyads, the surrogate’s distribution was generated in MATLAB 2024b^®^, while statistical analyses were performed in R (v. 4.4.2).

#### 2.5.2. EDA Sensing Dataset Linear Mixed Model

The participants were divided into three groups, each led by one of two different trainers, as illustrated in Figure 2. To evaluate the relationships within the electrodermal dataset, a linear mixed-effect model (LMM) was employed. The following model was applied to both physiological descriptors, DA and *I*, for both window lengths (60 s and 180 s):

(15) $$Meas_{aggregated}∼Phase\times Session+Trainer+Sex+\left(1|Subject\right)$$

where

- $Meas_{aggregated}$: The mean value of DA or *I* calculated for each subject across all windows for a given duration (60 s or 180 s).
- Phase: Represents the experimental condition (rest or meditation).
- Session: Represents the temporal progression (first or last session).
- Trainer: Included as a fixed effect, as the limited number of levels (two trainers) precludes its inclusion as a random effect.
- Sex: The biological sex of the subject.
- (1|Subject): The random intercept for each subject, accounting for inter-individual variability. Note that a nested structure (e.g., Trainer/Subject) was not implemented due to insufficient variance across the trainer groups. Moreover, the nested couple nested structure (e.g., Couple/Subject) was not implemented due to no estimable systematic variance attributable to couple membership beyond what is already captured at the subject level.

The significance of model effects was assessed using Type II ANOVA with Satterthwaite’s approximation.

The analyses were conducted using R (version 4.4.2) and packages lmerTest (v. 3.1-3) and lme4 (v. 1.1-37).

### 2.6. Synchronization Benchmark

Additionally, to establish the construct validity of the DA in the assessment of synchrony on the synthetic dataset, the results were benchmarked against a classical phase-based metric. Specifically, the Mean Phase Coherence (MPC) [43,44] was adopted as a summary index to quantify phase locking between two time series independently of their amplitude dynamics. Given two time series *x* and *y*, with $\phi_{x}$ and $\phi_{y}$ representing their respective instantaneous phases, the MPC metric is computed as follows:

(16) $$\mathrm{MPC}=\left|\frac{1}{N}\sum_{k=0}^{N-1}e^{i(\phi_{x}\left(t_{k}\right)-\phi_{y}\left(t_{k}\right))}\right|$$

where $t_{k}$ represents the time resolution steps, and *N* is the total sample number of each signal. The final values represents a summary of the strength of phase synchronization. This benchmark was selected because the synthetic model emulates cardiorespiratory interactions, where slower respiratory oscillations exert an asymmetrical influence on faster cardiac dynamics [35]. Phase-based coupling analysis constitutes a well-defined and established approach for characterizing such directional phenomena [45,46]. Spearman correlations were computed between MPC and both duplex Average Degree (DA) and inter-layer mutual information ($I_{x_{1},y_{1}}$). Correlations were evaluated separately at each window length to avoid confounding by recording duration. At each window length, the analysis included the 120 simulated realizations obtained across the four coupling conditions (30 realizations per coupling level).

## 3. Results

### 3.1. Statistical Analysis on Synthetic Data (Rössler System)

Visibility graph analysis of the coupled Rössler systems was used to evaluate the dependence of network features (DA and *I*) on window length (*N*) and coupling strength ($k_{1}$–$k_{4}$). Single-layer, duplex, and multiplex configurations were compared.

#### 3.1.1. Single Layer (Rössler System)

**Figure 4:**
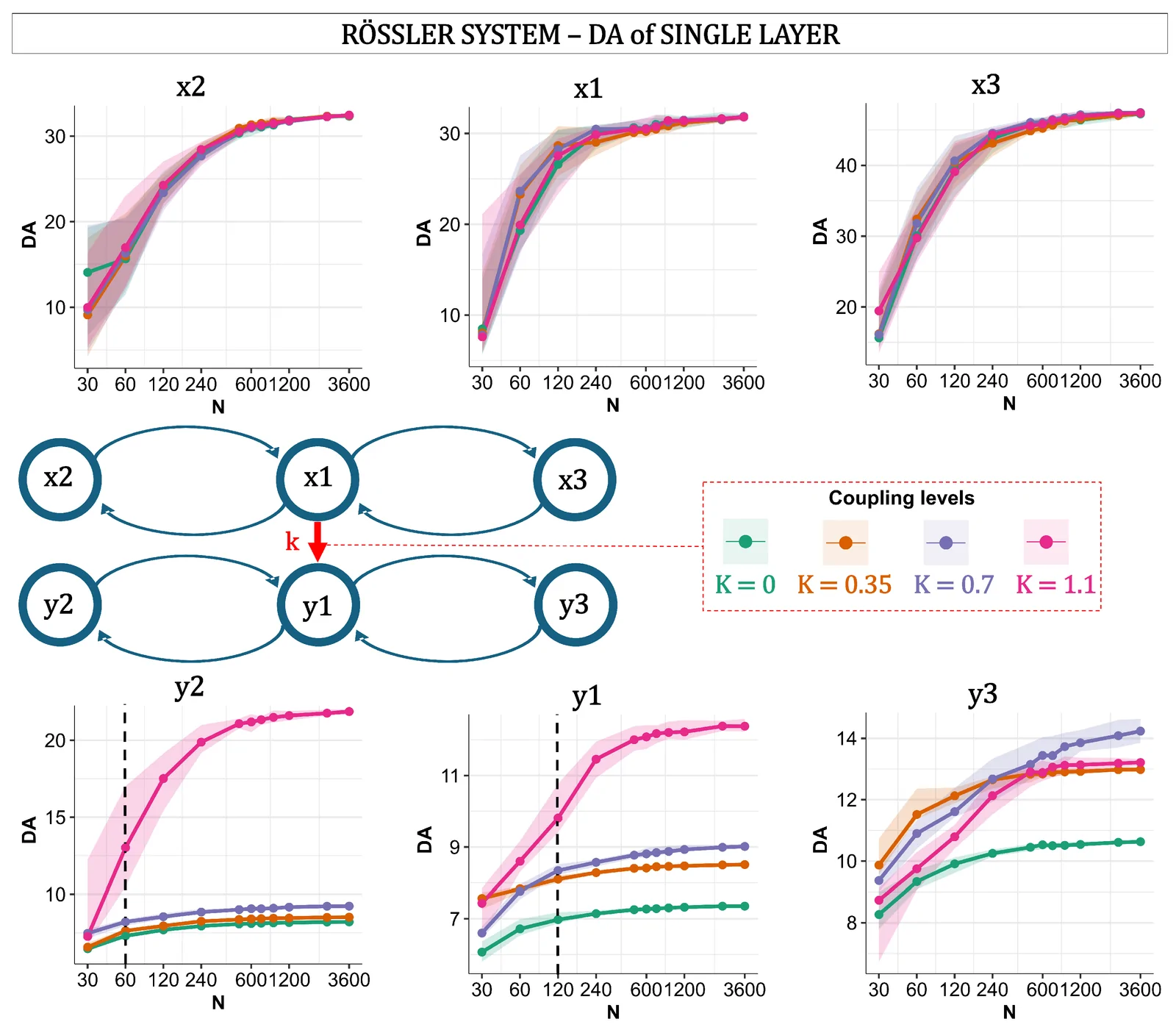
Degree (DA) of two unidirectionally coupled Rössler systems as a function of window length (*N*). The central schematic shows two three-variable Rössler attractors, $X=(x_{1},x_{2},x_{3})$ and $Y=(y_{1},y_{2},y_{3})$, with unidirectional coupling from $x_{1}$ to $y_{1}$. Plots show DA for four coupling levels ($k_{1}$–$k_{4}$). Solid lines and filled circles indicate the median, while shaded bands represent the interquartile range (IQR). Window length is shown on a logarithmic scale (N∈30,60,120,240,480,600,720,900,1200,2400,3600). *Y*-axis limits are independently scaled across panels. Black dashed lines indicate the minimum window length ($N_{\mathrm{stable}}$) at which all coupling levels are statistically distinct (p<0.05). (**Top**) Panels ($x_{2},x_{1},x_{3}$) show the driver system *X*, for which its DA is unaffected by coupling. (**Bottom**) Panels ($y_{2},y_{1},y_{3}$) show the driven system *Y*.

In the single-layer configuration, the DA was evaluated independently for the six state variables ($x_{1},x_{2},x_{3},y_{1},y_{2},y_{3}$). The results are shown in Figure 4. The driver variables ($x_{1},x_{2},x_{3}$) showed no significant differences across coupling levels for any window length (all p>0.05). In contrast, the response subsystem (y) was sensitive to coupling strength, with significant differences detected even at the shortest window length (N=30). For the directly driven variable $y_{1}$, DA significantly discriminated the baseline condition ($k_{1}$) from all higher coupling levels ($k_{2}$, $k_{3}$, $k_{4}$) at N=30 (e.g., $k_{1}$ vs. $k_{2}$: $p<10^{-9}$; $k_{1}$ vs. $k_{4}$: $p<10^{-6}$). The indirect response variables ($y_{2}$, $y_{3}$) also showed significant differences at N=30. However, $y_{3}$ displayed a non-monotonic pattern, with higher DA values at lower coupling strengths (e.g., $k_{2}>k_{3}>k_{4}$; $k_{2}$ vs. $k_{4}$: $p<10^{-7}$).

#### 3.1.2. Duplex (Rössler System)

**Figure 5:**
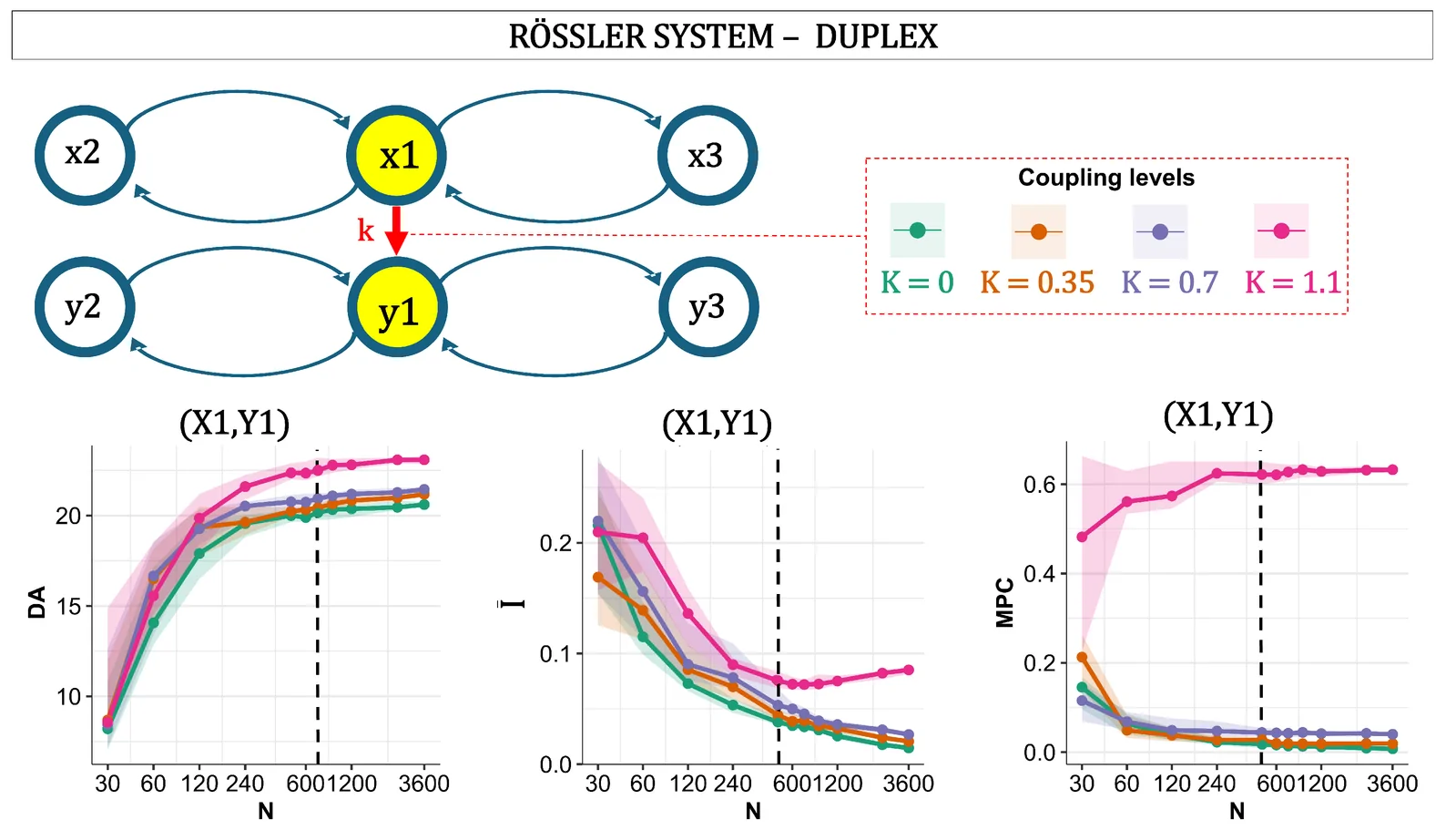
Duplex assessment. Degree (DA) and mutual information (*I*) and phase-coherence MPC of two unidirectionally coupled Rössler systems as a function of window length (*N*). The upper schematic shows two three-variable Rössler attractors, $X=(x_{1},x_{2},x_{3})$ and $Y=(y_{1},y_{2},y_{3})$, with unidirectional coupling from $x_{1}$ to $y_{1}$. Plots show DA for four coupling levels ($k_{1}$–$k_{4}$), the series observed are highlighted in yellow. Solid lines and filled circles indicate the median, while shaded bands represent the interquartile range (IQR). Window length is shown on a logarithmic scale (N∈30,60,120,240,480,600,720,900,1200,2400,3600). *Y*-axis limits are independently scaled across panels. Black dashed lines indicate the minimum window length ($N_{\mathrm{stable}}$) at which all coupling levels are statistically distinct (p<0.05).

The analysis was then extended to the $x_{1}$–$y_{1}$ duplex representation to assess inter-layer interactions (see Figure 5). At the shortest window lengths (N=30), neither the duplex DA nor the inter-layer *I* showed significant differences between coupling levels. Significant differences in *I* emerged from N=60, mainly for comparisons involving the strongest coupling condition (e.g., $k_{1}$ vs. $k_{4}$: $p<10^{-6}$). Duplex DA required longer windows, with the first significant differences observed at N=120 ($k_{1}$ vs. $k_{4}$: p=0.012). For both metrics, pairwise differences became more pronounced with increasing window length.

#### 3.1.3. Multiplex (Rössler System)

**Figure 6:**
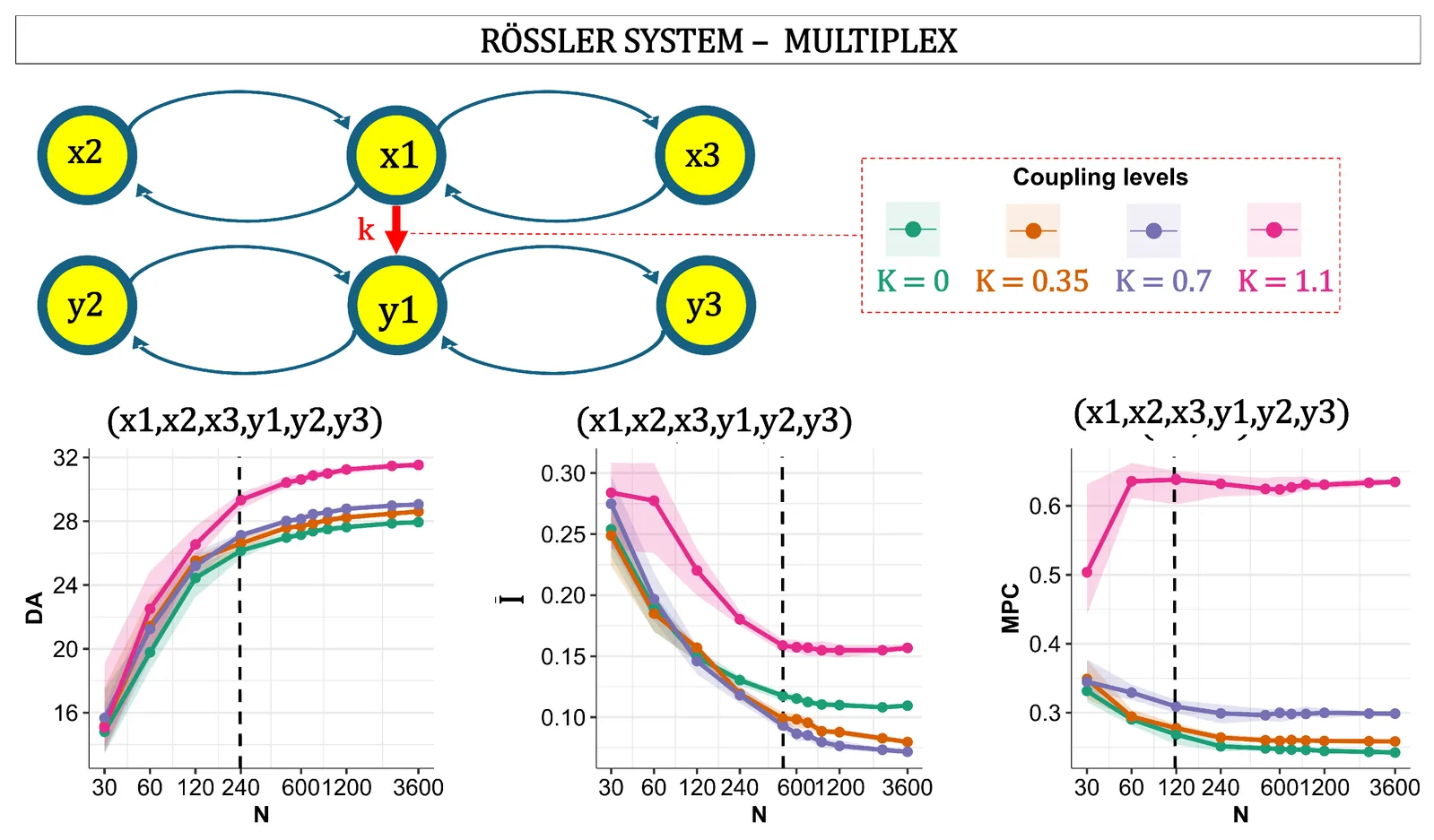
Multiplex assessment. Degree (DA), mutual information ($\left(\bar{I}\right)$), and phase-coherence MPC of two unidirectionally coupled Rössler systems as a function of window length (*N*). The upper schematic shows two 3-variable Rössler attractors, $X=(x_{1},x_{2},x_{3})$ and $Y=(y_{1},y_{2},y_{3})$, with unidirectional coupling from $x_{1}$ to $y_{1}$. Plots show DA for four coupling levels ($k_{1}$–$k_{4}$), and the series observed are highlighted in yellow. Solid lines and filled circles indicate the median, while shaded bands represent the interquartile range (IQR). Window length is shown on a logarithmic scale (N∈30,60,120,240,480,600,720,900,1200,2400,3600). *Y*-axis limits are independently scaled across panels. Black dashed lines indicate the minimum window length ($N_{\mathrm{stable}}$) at which all coupling levels are statistically distinct (p<0.05).

The six-layer multiplex configuration ($x_{1},y_{1},x_{2},y_{2},x_{3},y_{3}$) provided an integrated representation of the system dynamics (results reported in Figure 6). Significant differences were observed from N=60. At this window length, multiplex DA differentiated the baseline from higher coupling levels (e.g., $k_{1}$ vs. $k_{4}$: p=0.012), while multiplex $\bar{I}$ showed a strong response to the highest coupling condition (e.g., $k_{1}$ vs. $k_{4}$: $p<10^{-12}$). With increasing window length, pairwise differences became progressively stronger, including comparisons between adjacent coupling levels.

#### 3.1.4. Effect of Window Length and Feature Stabilization

Across all configurations, feature estimates were more variable at short window lengths and began to stabilize at approximately N=480, approaching a plateau for N≥1000. The window length required to detect coupling-related differences depended on the network representation and metric. Changes in the response subsystem were already detected at N=30 in the single-layer analysis. For inter-layer interactions, significant differences were observed from N=60 for both duplex *I* and multiplex features, whereas duplex DA required N=120. Increasing the window length also improved the resolution of statistical contrasts. At short windows (N≤120), significant differences were mainly observed for comparisons involving the strongest coupling condition (e.g., $k_{1}$ vs. $k_{4}$), whereas at longer windows (N≥240), differences between adjacent coupling levels could also be detected (e.g., $k_{2}$ vs. $k_{3}$).

#### 3.1.5. Synchronization Benchmark and Convergent Validity

To benchmark the proposed network features, Mean Phase Coherence (MPC) was evaluated as a classical phase-based measure of synchronization. MPC was computed either from the directly coupled pair ($x_{1},y_{1}$) or across the six system variables using the corresponding aggregate value for the full system. The Kruskal–Wallis test showed significant differences across coupling strengths ($k_{1}$–$k_{4}$) at all evaluated window lengths (N≥30, all p<0.001). The effect size ($\epsilon^{2}$) increased with window length, from 0.46–0.48 at N=30 to 0.94 for N≥3600. Post hoc comparisons showed that MPC was sensitive to strong coupling differences even at N=30. At this window length, the metric clearly separated the baseline from the highest coupling condition ($k_{1}$ vs. $k_{4}$: $p<10^{-13}$, r=0.83), while smaller differences between coupling levels were progressively resolved with increasing window length. From N=120 onward, the statistical resolution increased, with significant differences emerging between adjacent coupling levels. Effect sizes for comparisons involving $k_{4}$ remained large (r=0.86).

To assess the association between the VG-derived descriptors and conventional phase synchronization, duplex DA and inter-layer mutual information ($I_{x_{1},y_{1}}$) for the directly coupled $x_{1}-y_{1}$ pair were correlated with MPC separately at each window length. Spearman correlations were computed across the 120 observations available at each window length (30 realizations for each of the four coupling conditions).

The association between DA and MPC depended strongly on window length. At N=30, DA showed a weak positive correlation with MPC (ρ=0.32, $p_{\mathrm{FDR}}$ <0.001), whereas no significant association was observed at N=60 (ρ=−0.08, $p_{\mathrm{FDR}}=0.390$) or N=120 (ρ=0.13, $p_{\mathrm{FDR}}=0.180$). From N=240 onward, the association became consistently positive and strong, increasing from ρ=0.60 at N=240 to ρ=0.90 at N=3600 (all $p_{\mathrm{FDR}}<0.001$).

*I* showed a similar window-length-dependent pattern. No significant association with MPC was observed at N=30 (ρ=0.07, $p_{\mathrm{FDR}}=0.447$). Significant positive correlations emerged at N=60 and N=120 (ρ=0.33 for both; $p_{\mathrm{FDR}}<0.001$) and became stronger from N=240 onward, ranging from ρ=0.55 at N=240 to ρ=0.93 at N=3600 (all $p_{\mathrm{FDR}}<0.001$). Complete correlation results are reported in Supplementary Table S17.

### 3.2. Statistical Analysis on Multichannel Cardiovascular Sensing Dataset (HYPOL)

The VG framework was subsequently evaluated on multichannel cardiovascular sensing data. Sex-related differences were evaluated using the Mann–Whitney U test for independent samples.

#### 3.2.1. Single Layer (HYPOL)

**Figure 7:**
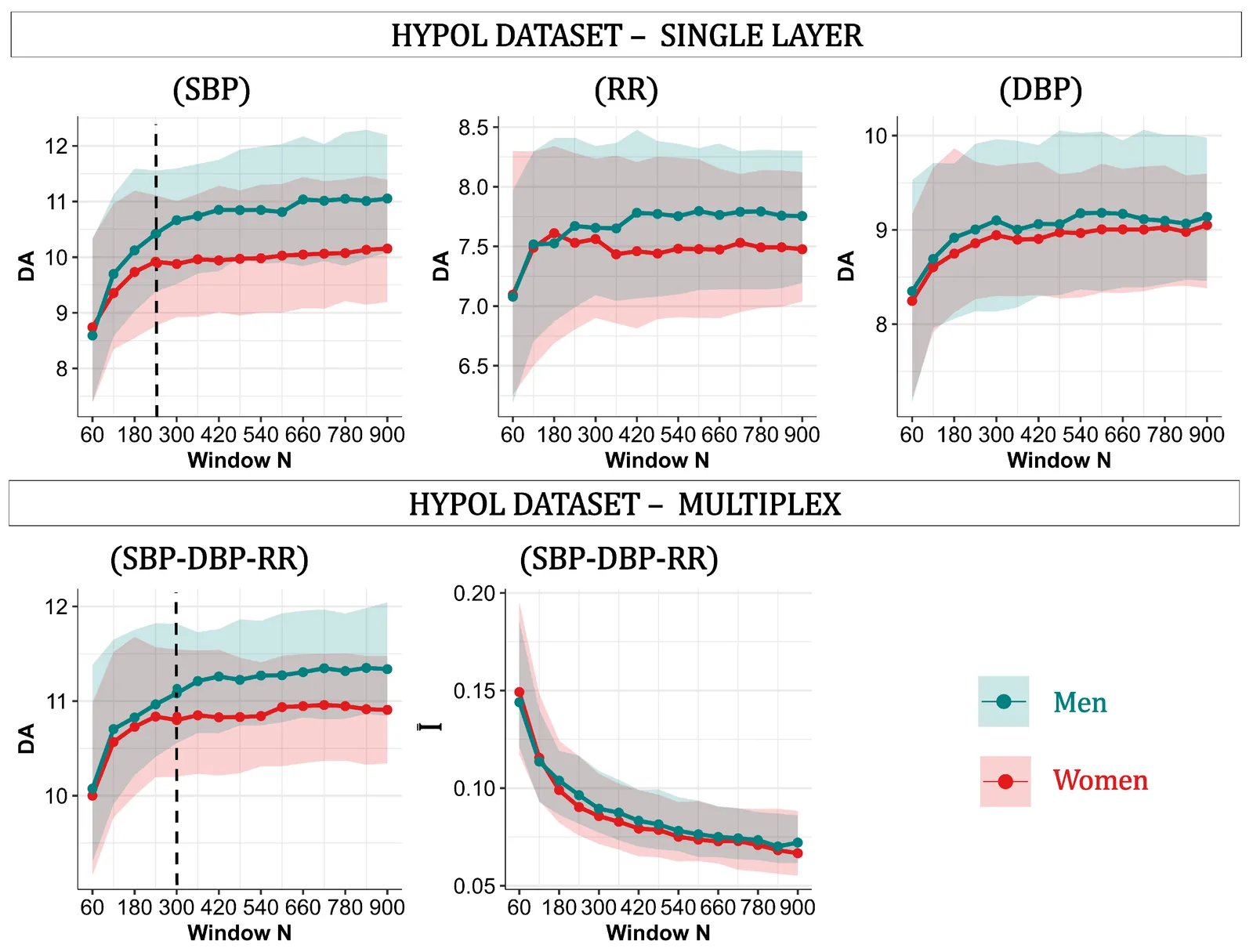
Multiplex and single-layer assessment. (**Upper Panel**) Degree (DA) for the single-layer configuration of each physiological series (RR, SBP, and DBP) highlighting sex-related cardiovascular differences as a function of window *N* (seconds). (**Lower Panel**) Degree (DA) and mutual information (*I*) for the multiplex configuration (RR, SBP, and DBP). *Y*-axis limits are independently scaled across panels. Solid lines and filled circles indicate the median, while shaded bands represent the interquartile range (IQR). Black dashed lines indicate the earliest window duration at which a significant sex difference was observed after FDR correction (p<0.05).

For the single-layer configuration, DA was evaluated independently for RR, SBP, and DBP (results reported in Figure 7, upper panel). The RR and DBP pressure series showed no significant sex differences across any window length (all p>0.05). After N=420, a nearly significant trend is observed for RR, concurrently with the results presented in [47]. After FDR, it is no longer significant. In contrast, systolic blood pressure showed significant differences from N=240 $p_{\mathrm{FDR}}=0.021$) onward. The difference increased with window length, with higher DA values in men than women.

#### 3.2.2. Duplex (HYPOL)

**Figure 8:**
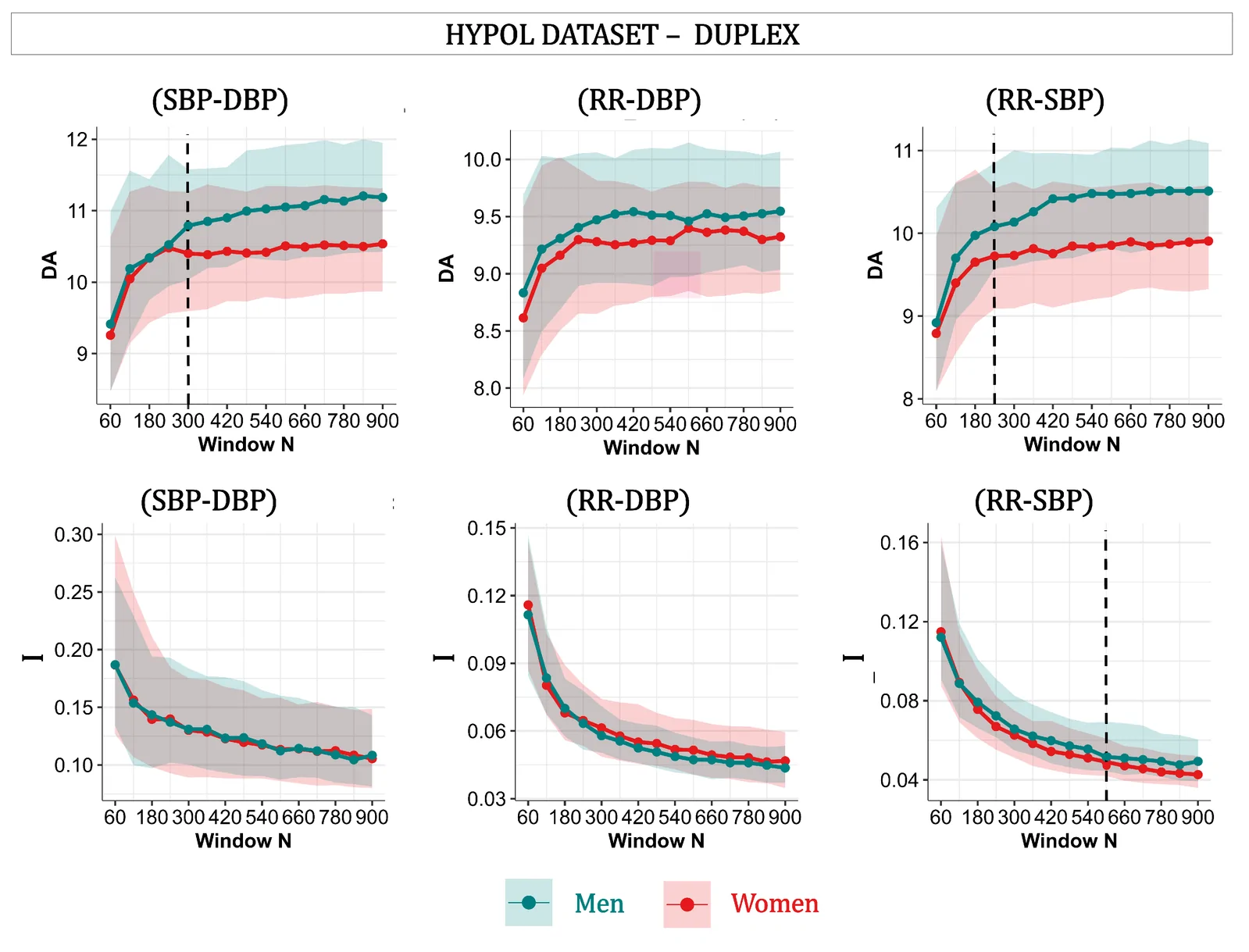
Duplex and single-layer assessment. Degree (DA (**upper panel**)) and (*I* (**lower panel**)) for the duplex configuration of paired physiological series (RR, SBP, and DBP) highlighting sex-related cardiovascular differences as a function of window *N*(seconds). *Y*-axis limits are independently scaled across panels. Solid lines and filled circles indicate the median, while shaded bands represent the interquartile range (IQR). Black dashed lines indicate the earliest window duration at which a significant sex difference was observed after FDR correction (p<0.05).

For duplex representations, the RR–DBP configuration showed no significant sex differences in either DA or *I* at any window length (all ps>0.05) (results reported in Figure 8). In contrast, configurations including the systolic component showed significant differences. For the RR–SBP duplex, both DA and *I* differed significantly from N=240 ($p_{\mathrm{FDR}}=0.047$ and $p_{\mathrm{FDR}}=0.028$, respectively), with stronger effects at longer scales. The systolic–diastolic duplex DA also showed significant differences from N=300 ($p_{\mathrm{FDR}}=0.027$), whereas its *I* remained non-significant across all scales.

#### 3.2.3. Multiplex (HYPOL)

The multiplex configuration combined all three physiological series (results reported in Figure 7, lower panel). Significant sex differences in multiplex DA emerged N=300 ($p_{\mathrm{FDR}}=0.022$, r=0.17) and increased with window length. The longest window reached the strongest significance (e.g., at N=720–900: p<0.0005, r≈0.26). In contrast, multiplex $\bar{I}$ showed no significant sex differences at any window length (all p>0.36).

### 3.3. Statistical Analysis on Wearable EDA Signals During the MBSR Intervention

The primary inferential analysis was based on the linear mixed-effect models described in Section 2.5.2. Paired Wilcoxon tests were retained as non-parametric sensitivity analyses and are reported in Tables S23–S26 in the Supplementary Materials.

#### 3.3.1. Results on EDA Sensing Dataset Surrogates Statistical Analysis

To assess whether the physiological organization of real dyads differed from a broad dyad-unspecific reference distribution, real trainer–trainee recordings were compared with the empirical pseudo-dyad distribution. The number of surrogates was $n_{\mathrm{surr}}=2080$ for N=180 s and $n_{\mathrm{surr}}=17,183$ for N=60 s.

We evaluated the empirical cumulative distribution functions using one-tailed two-sample Kolmogorov–Smirnov (KS) tests (testing the directional hypothesis $F_{\mathrm{real}}\left(x\right)<F_{\mathrm{surr}}\left(x\right)$, denoting that descriptor values in real dyads were stochastically greater than those in the surrogate reference distribution) alongside nonparametric Mann–Whitney *U* tests. The maximal vertical distance between cumulative distributions, denoted as *D*, was employed as an effect-size metric. To control for multiple testing, *p*-values across the four condition contrasts were adjusted using the false discovery rate (FDR, Benjamini–Hochberg procedure, denoted as $p_{\mathrm{FDR}}$).

Real dyads in the final session significantly exceeded the global surrogate benchmark across both network-topology and information-theoretic metrics:

1. **DA.** In the final meditation session, real dyads exhibited significantly higher DA values than the surrogate reference distribution across both temporal window lengths:
  - For the 60 s window (N=60 s), D=0.245, with $p=1.85\times 10^{-8}$ ($p_{\mathrm{FDR}}=7.41\times 10^{-8}$). The corresponding Mann–Whitney U test yielded $p=5.25\times 10^{-10}$ ($p_{\mathrm{FDR}}=2.10\times 10^{-9}$), with $n_{\mathrm{real}}=135$ and $n_{\mathrm{surr}}=17,183$.
  - For the 180 s window (N=180 s), the KS statistic was D=0.252, with p=0.0020 ($p_{\mathrm{FDR}}=0.0082$). The Mann–Whitney U test yielded p=0.0017 ($p_{\mathrm{FDR}}=0.0067$), with $n_{\mathrm{real}}=45$ and $n_{\mathrm{surr}}=2080$.
2. ***I*.** Consistent significant coupling was observed for the mutual-information metric *I*:
  - In the final meditation session, real interactions significantly exceeded the surrogate baseline across both window lengths: D=0.191, $p=2.03\times 10^{-5}$ ($p_{\mathrm{FDR}}=8.12\times 10^{-5}$) for N=60 s; and D=0.248, p=0.0025 ($p_{\mathrm{FDR}}=0.0099$) for N=180 s. The corresponding Mann–Whitney U tests yielded $p=1.07\times 10^{-4}$ ($p_{\mathrm{FDR}}=4.26\times 10^{-4}$) and p=0.0012 ($p_{\mathrm{FDR}}=0.0049$), respectively, demonstrating robust moderate effect sizes.
  - In the initial baseline-rest session, only significant coupling was detected exclusively for the 180 s window by the Mann–Whitney U test (p=0.0205, $p_{\mathrm{FDR}}=0.0410$), while the KS test exhibited a trend toward significance (p=0.0547, $p_{\mathrm{FDR}}=0.0729$, D=0.312).
  - In the final baseline-rest session, real resting-state coupling also significantly diverged from the global surrogate distribution: for N=60 s, D=0.231, p=0.0084 ($p_{\mathrm{FDR}}=0.0169$; Mann–Whitney U p=0.0097, $p_{\mathrm{FDR}}=0.0194$). For N=180 s, real rest displayed a substantial effect size (D=0.380, p=0.0136, $p_{\mathrm{FDR}}=0.0271$), with the Mann–Whitney U test showing a marginal trend after correction (p=0.0452, $p_{\mathrm{FDR}}=0.0602$).

The complete results are reported in Tables S27 and S28 in the Supplementary Materials.

#### 3.3.2. Results on EDA Sensing Dataset Linear Mixed Model

**Figure 9:**
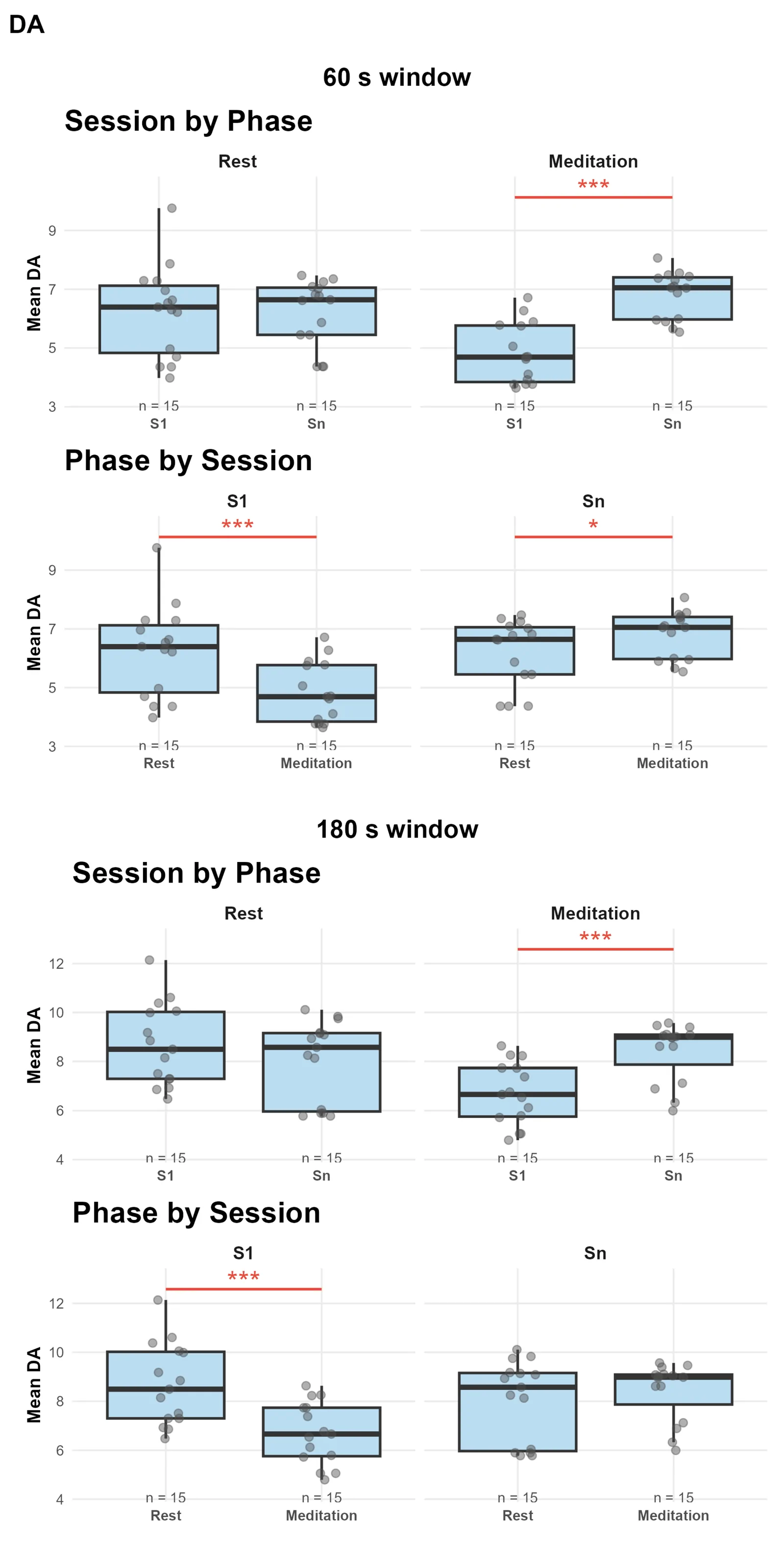
Boxplots of the post hoc comparisons of the DA values, obtained from LMM $DA_{mean}∼Phase\times Session+Trainer+Sex+\left(1|Subject\right)$, for both window lengths (60 s and 180 s). Post hoc comparisons were applied to the Session×Phase interaction, considering two families of contrasts: session by phase (first (*S1*) vs. last session (*Sn*), within rest and within meditation) and phase by session (rest vs. meditation, within *S1* and within *Sn*). Window-level values were averaged within each subject, phase, and session. Red lines indicate significant comparisons (* = p<0.05, *** = p<0.001); the complete results are reported in Tables S27 and S28 in the Supplementary Materials. Multiple-comparison correction was applied within each post hoc comparison group.

**Figure 10:**
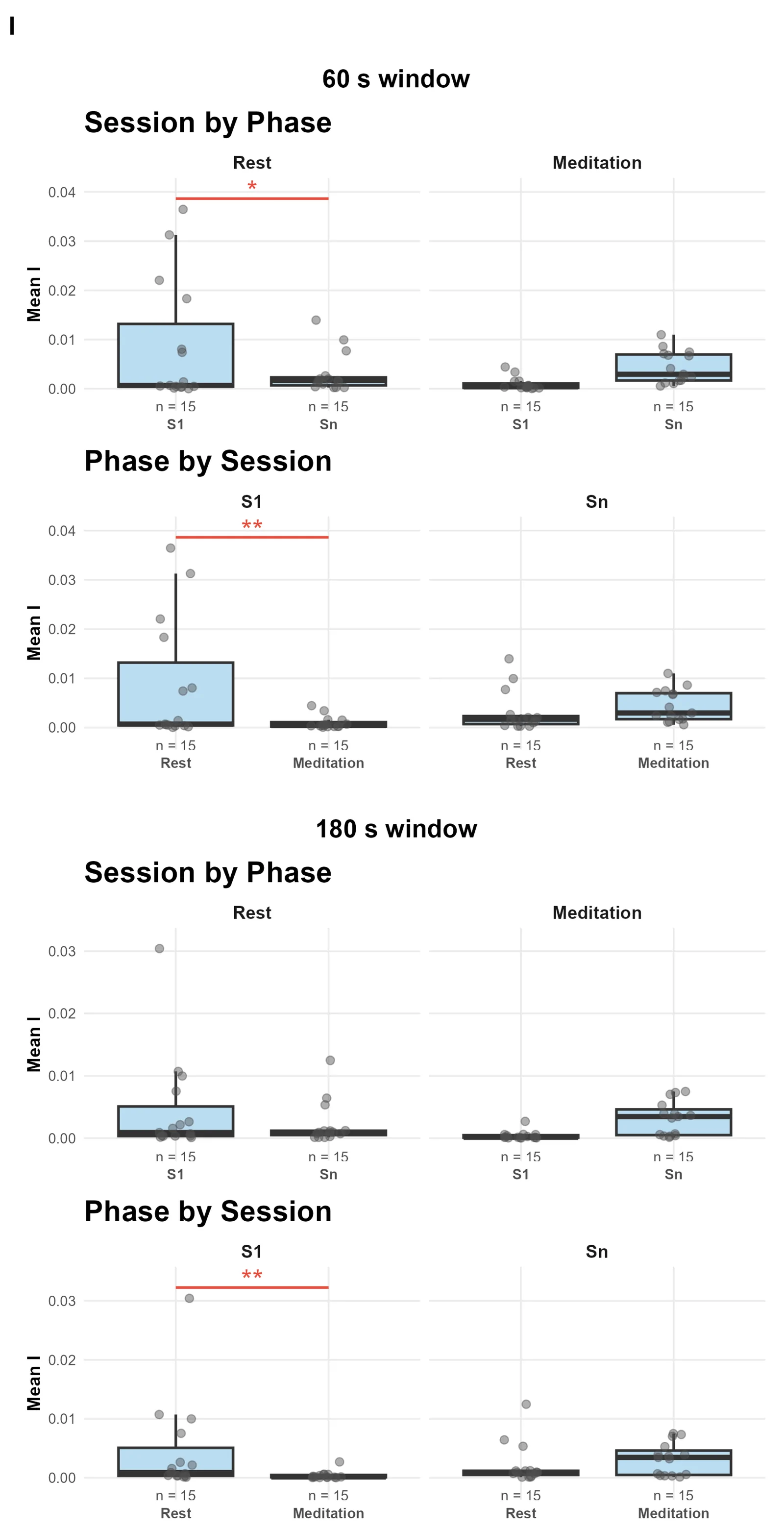
Boxplots of the post hoc comparisons of the *I* values, obtained from the LMM $I_{mean}∼Phase\times Session+Trainer+Sex+\left(1|Subject\right)$, for both window lengths (60 s and 180 s). Post hoc comparisons were applied to the Session×Phase interaction, considering two families of contrasts: session by phase (first (*S1*) vs. last session (*Sn*), within rest and within meditation) and phase by session (rest vs. meditation, within *S1* and within *Sn*). Window-level values were averaged within each subject, phase, and session. Red lines indicate significant comparisons (* = p<0.05, ** = p<0.01); the complete results are reported in Tables S27 and S28 in the Supplementary Materials Multiple-comparison correction was applied within each post hoc comparison group.

Figure 9 and Figure 10 show the boxplots illustrating the post hoc comparisons for DA and *I*, respectively, obtained from the LMM. Type II ANOVA revealed a significant Phase × Session interaction for all four LMMs: DA at 60 s, F(1,42)=24.57, p<0.001, $\eta_{p}^{2}=0.37$; DA at 180 s, F(1,42)=19.98, p<0.001, $\eta_{p}^{2}=0.32$; *I* at 60 s, F(1,42)=6.99, p=0.011, $\eta_{p}^{2}=0.14$; and *I* at 180 s, F(1,42)=5.72, p=0.021, $\eta_{p}^{2}=0.12$. Follow-up pairwise comparisons indicated that DA increased from the first to last session during meditation for both the 60 s and 180 s windows, with large effects (d=−2.50 and d=−1.67, respectively), whereas no significant session-related change was observed during rest. At the first session, DA was significantly lower during meditation than rest for both window lengths (p<0.001); at the last session, this phase difference remained significant for the 60 s window (p=0.037) but not for the 180 s window (p=0.307). For *I*, the 60 s model showed a significant difference between meditation and rest at the first session (p=0.003) but not at the last session, while the session-related change was significant only during rest (p=0.027). For the 180 s window, *I* differed significantly between meditation and rest at the first session (p=0.009), with no significant within-phase change across sessions. Neither trainer nor sex showed a significant main effect in any model.

The complete results are reported in Tables S29 and S30 in the Supplementary Materials.

## 4. Discussion

This study evaluated a multilayer VG framework for the analysis of short multichannel physiological recordings across three complementary settings: a controlled simulation study, beat-to-beat cardiovascular recordings (HYPOL public dataset), and wearable EDA recordings acquired during an MBSR intervention. The analyses support a distinction between two complementary aspects of the network representation. Average Degree (DA) characterizes the topological organization of individual visibility graphs and their multiplex aggregation, whereas inter-layer mutual information (*I*) explicitly quantifies statistical dependence between the degree sequences of different layers. This distinction is important because changes in within-layer topology and changes in cross-layer dependence need not provide equivalent information about an interacting system. By combining synthetic validation with cardiovascular and wearable physiological recordings, we demonstrate that the proposed framework enables robust characterization of physiological interactions from ultrashort recordings without phase-space reconstruction.

The single-layer control of the coupled Rössler system substantially clarifies the interpretation of the synthetic results. The driver variables $x_{1},x_{2},x_{3}$ were unaffected by coupling strength, whereas the response subsystem $y_{1},y_{2},y_{3}$ exhibited coupling-dependent topological changes already detectable at short observation lengths. In particular, the DA of the directly driven variable $y_{1}$ distinguished the uncoupled condition from coupled conditions at N=30. Thus, the present findings do not support a general advantage of a multiplex representation over single-layer analysis for detecting coupling-induced changes. Instead, they show that coupling can modify the intrinsic topology of the response dynamics and that these modifications can themselves be detected by single-layer VGs.The multiplex descriptors provide complementary rather than uniformly superior information. In the $x_{1}-y_{1}$ duplex, inter-layer mutual information detected coupling-related differences from N=60, whereas duplex DA required longer windows. Moreover, because the identity inter-layer links contribute the same M−1 edges to every state node, their contribution to multiplex DA is constant across coupling conditions. Consequently, coupling-related changes in multiplex DA arise from changes in the underlying intra-layer visibility structure rather than from the identity links themselves. Inter-layer mutual information therefore provides a more direct descriptor of cross-layer dependence in the proposed framework.

Comparisons with MPC further support this interpretation. MPC discriminated coupling levels even at the shortest observation lengths, confirming the sensitivity of an established phase-based synchronization measure in the simulated system. The correlations between MPC and the VG-derived descriptors were strongly dependent on observation length. For DA, the association with MPC was weak at very short windows and was not significant at N=60 and N=120, but it became moderate to strong from N=240 onward and progressively increased at longer windows. Inter-layer mutual information showed no significant association with MPC at N=30, weak-to-moderate positive associations at N=60 and N=120, and strong positive associations from N=240 onward. These results provide evidence of convergent validity while also showing that neither DA nor *I* should be regarded as equivalent to phase synchronization. Rather, the descriptors capture related but distinct properties of the coupled dynamics. The increasing agreement at longer windows also highlights the trade-off between temporal localization and statistical stability. Across VG descriptors, estimates became markedly more stable at approximately N=480, consistent with previous observations that VG-based metrics become more reliable as the number of available samples increases [48].

The HYPOL dataset [36] focused on intra-subject multisystem interactions, specifically the relationship between cardiac and vascular dynamics, a key mechanism in cardiovascular regulation. The HYPOL analysis extended the framework to irregularly sampled beat-to-beat cardiovascular recordings and, importantly, the analysis avoided uniform resampling. This sensitivity analysis reduces the possibility that the observed effects were driven by interpolation-induced structure. Sex-related differences were not uniformly distributed across cardiovascular variables or network configurations. At the single-layer level, significant differences were primarily observed for systolic blood pressure, whereas RR and DBP did not show robust differences after multiple-comparison correction. Similarly, duplex effects were concentrated in configurations containing SBP. The RR-SBP duplex showed sex-related differences in both DA and inter-layer mutual information at selected observation lengths, whereas RR-DBP showed no significant differences, and SBP-DBP differences were primarily expressed in DA. In the three-layer RR-SBP-DBP multiplex, DA differed between sexes from intermediate-to-long (300 to 900 s) window durations, whereas mean inter-layer mutual information did not. These findings therefore suggest a more specific interpretation than a generalized sex difference in cardiovascular “complexity” or coupling. The pattern appears to be predominantly associated with the systolic component and, for inter-layer dependence, particularly with the RR-SBP relationship. The observed effects are consistent with previous evidence of sex-related differences in cardiovascular dynamics [47,49,50,51,52], but the present results do not establish a specific autonomic or causal mechanism. In particular, the absence of a significant difference in three-layer inter-layer mutual information argues against interpreting the findings as evidence of globally reduced cardiovascular coupling in women. Instead, they indicate that sex-related differences may depend on the physiological subsystem, network representation, and temporal scale considered. The generally small-to-moderate effect sizes observed in HYPOL further support a cautious interpretation of these differences.

The wearable EDA analysis investigated whether the same framework could characterize changes in interpersonal physiological organization. The EDA dataset addressed inter-individual interactions, introducing two main novelties: the use of SMNA series and the analysis of physiological interactions during a mindfulness protocol involving infertile couples. Because participants contributed repeated observations and multiple trainees shared instructors, the analysis included linear mixed-effect models with subject as a random intercept and trainer as a fixed effect. A significant Phase × Session interaction was observed for both descriptors and both window lengths. The most consistent longitudinal result concerned DA, which increased during meditation from the first to the last MBSR session at both 60 s and 180 s scales, whereas no corresponding session-related change was observed during rest. For inter-layer mutual information, the significant interaction was associated with a more heterogeneous pattern of post hoc contrasts, and the longitudinal meditation effect was less robust after accounting for the repeated-measures structure. This difference further illustrates that DA and inter-layer mutual information should not be interpreted interchangeably. The pseudo-dyad analysis provided an additional test of whether the observed trainer–trainee organization exceeded a dyad-unspecific reference distribution. During the final meditation session, real dyads showed higher DA and inter-layer mutual information than the surrogate benchmark at both temporal scales. Inter-layer mutual information also differed from the surrogate distribution during final-session rest, although the evidence was less consistent across statistical tests at 180 s. These findings indicate that the structure observed in real trainer–trainee recordings cannot be fully reproduced by the broad pseudo-dyad distribution used here, and they are consistent with the presence of a dyad-specific component in the physiological organization emerging across the intervention. Taken together, the longitudinal and surrogate analyses suggest that the changes observed at the end of the MBSR program were not only limited to a generic modification of individual SMNA topology but also involved physiological organization specific to the actual trainer–trainee pairing.

The increasing availability of wearable and multichannel physiological sensing technologies requires computational methods capable of extracting robust biomarkers from ultrashort and heterogeneous recordings. Unlike many nonlinear approaches, the proposed framework can be applied across heterogeneous physiological sensing modalities without modification of the computational pipeline. The present work suggests that multilayer VG analysis represents a promising candidate because it is free of phase-space embedding parameters and applicable across different sensing modalities, ranging from cardiovascular monitoring to EDA. The absence of a parameter selection step (e.g., embedding dimension and time delay as in Takens’ reconstruction) is particularly advantageous for EDA signals, where phase-space reconstruction can be challenging even in univariate settings.

Several limitations should be considered. First, the interpersonal dataset was small and comprised 15 trainees distributed across only three groups and two instructors. The HYPOL sample consists of healthy young adults from a relatively restricted population, limiting generalizability. For EDA analysis, although the mixed-effect models account for repeated observations within trainees and adjust for instructor identity, residual dependence may remain among dyads sharing an instructor recording or an MBSR group. The small number of groups and instructors limits the estimation of these additional sources of variation. Moreover, the EDA findings were obtained using a single preprocessing configuration. Their sensitivity to cvxEDA settings, artifact handling, and the sampling rate of the estimated SMNA was not systematically evaluated. Finally, the proposed framework is free of phase-space embedding parameters, but it still requires analytical choices concerning window duration, VG formulation, preprocessing, network configuration, and descriptor selection.

## 5. Conclusions

This study evaluated a multilayer VG framework for characterizing short multichannel physiological recordings using complementary topological and inter-layer descriptors. The simulation study showed that coupling-induced changes can already be detected in the topology of individual response signals, demonstrating that a multiplex representation is not inherently more sensitive than single-layer analysis. In contrast, inter-layer mutual information provides complementary information specifically related to statistical dependence across synchronized network layers. Its association with MPC supports convergent validity while indicating that network-based and phase-based measures capture related but non-equivalent properties of coupled dynamics.

The physiological applications further showed that the information provided by the framework depends on both the signal combination and the network descriptor. In the cardiovascular dataset, sex-related differences were predominantly associated with systolic blood pressure, with inter-layer differences being most evident in the interbeat interval-systolic blood pressure duplex rather than in the global three-layer mutual information. In the wearable EDA dataset, changes in trainer–trainee physiological organization across the MBSR intervention were identified, although their interpretation is limited by the small sample.

Future work should evaluate the framework in larger and more diverse physiological datasets and quantify its computational requirements under practical acquisition conditions.

## Data Availability

The synthetic datasets generated and analyzed during the current study are available from the corresponding author upon reasonable request. The HYPOL cardiovascular dataset is publicly available at https://hypol.ump.edu.pl/ (accessed on 5 April 2024). The wearable electrodermal activity dataset contains sensitive physiological and clinical data from human participants and is not publicly available due to ethical and privacy restrictions imposed by the study protocol and the approving Ethics Committee. Access to anonymized data may be considered on reasonable request, subject to approval by the corresponding author and the relevant Ethics Committee. The codes used are available at https://github.com/LauraLav99/A-Multilayer-Visibility-Graph-Framework-for-Ultrashort-Term-Analysis (accessed on 30 September 2026).

## Supplementary Materials

The following supporting information can be downloaded at https://www.mdpi.com/article/10.3390/s26206668/s1. **Tables S1–S4**. Statistical results of the Kruskal–Wallis omnibus tests for the synthetic Rössler system: single-layer Average Degree (DA) across all six state variables $(x_{1},x_{2},x_{3},y_{1},y_{2},y_{3}$) (S1); DA and inter-layer Mutual Information (I) for the duplex configuration ($x_{1},y_{1}$) (S2); DA and I for the six-layer multiplex configuration (S3); and Mean Phase Coherence (MPC) for the bivariate pair ($x_{1},y_{1}$) and averaged across all pairs of state variables (S4). **Tables S5–S16**. Post-hoc pairwise comparisons using the Mann–Whitney U test for the synthetic Rössler system, covering single-layer DA for each state variable (S5–S10); DA and (I) for the duplex configuration (S11–S12); MPC for the bivariate pair ($x_{1},y_{1}$) (S13); DA and I for the six-layer multiplex configuration (S14–S15); and MPC averaged across all pairs of state variables (S16). **Table S17**. Spearman rank correlation analysis between MPC and network measures (DA and I) for the bivariate series ($x_{1},y_{1}$), as a function of temporal window size. **Tables S18–S22**. Statistical results of the Mann–Whitney U tests comparing sex-related cardiovascular differences in the HYPOL dataset: single-layer DA for systolic, diastolic, and beat-to-beat series separately (S18); DA and (I) for duplex configurations formed by each pair of cardiovascular series (S19–S21); and DA and I for the three-layer multiplex configuration combining all three series (S22). **Tables S23–S26**. Paired Wilcoxon signed-rank test results comparing meditation and non-meditation conditions during the first and last sessions in the Trainer–Trainee dyad study, using EDA-derived SMNA visibility graphs. The analyses cover DA for 60-s and 180-s windows (S23–S24) and (I) for the same window lengths (S25–S26). **Tables S27–S28**. Surrogate-data analyses comparing real dyadic interactions with the global surrogate distribution for the DA (S27) and (I) (S28) descriptors across 60-s and 180-s windows. **Table S29**. Type II ANOVA results (Satterthwaite’s method) for linear mixed-effects models fitted to DA and I, computed using 60-s and 180-s aggregation windows. **Table S30**. Post-hoc comparisons from the linear mixed-effects models fitted to DA and I, focusing on the Session × Phase interaction. **Figure S1**. Boxplots of DA values derived from SMNA visibility graphs, comparing rest and meditation during the first and last sessions for 60-s and 180-s windows using the Wilcoxon test. **Figure S2**. Boxplots of I values derived from SMNA visibility graphs, comparing rest and meditation during the first and last sessions for 60-s and 180-s windows using the Wilcoxon test.

## Abbreviations

The following abbreviations are used in this manuscript:

VG	Visibility Graph
MBSR	Mindfulness-Based Stress Reduction
EDA	Electrodermal Activity
DA	Average Degree
I	Inter-Layer Mutual Information
RR	Interbeat Interval
SBP	Systolic Blood Pressure
DBP	Diastolic Blood Pressure
SMNA	Sudomotor Nerve Activity
HRV	Heart Rate Variability
KS	Kolmogorov–Smirnov
FDR	False Discovery Rate
LMM	Linear Mixed-Effect Model
MPC	Mean Phase Coherence
ECG	Electrocardiogram
