# Supplementary material for: A Multilayer Visibility Graph Framework for Ultrashort-Term Analysis of Multichannel Physiological Signals
Source: Sensors (Basel). 2026 Oct 21;26(20):6668. doi: 10.3390/s26206668 (PMC13709538; doi:10.3390/s26206668)
Supplement: Supplementary file 1 [file sensors-26-06668-s001.zip › sensors-4486942-supplementary.pdf]

Supplementary Materials: A Multilayer Visibility Graph Framework for Ultrashort-Term Analysis of Multichannel Physiological Signals

Laura Lavezzo [1], Anna Di Marco [1], Aleandra Viti[2,3], Rebecca Ciacchini[2], Ciro Conversano[2], Enzo Pasquale Scilingo[1] and Mimma Nardelli[1]*

S1. Synthetic Validation (Rössler System)

This section contains omnibus and post-hoc statistical test results evaluating the discriminative power of Average Degree (DA) and Mutual Information (I) across the simulated coupling strengths in the Rössler system configurations.

S1.1. Omnibus Tests

Table S1. Statistical results of the Kruskal-Wallis omnibus test for the single-layer Average Degree (DA) across all state variables ($x_1, x_2, x_3, y_1, y_2, y_3$) of the synthetic Rössler system. The table reports the H statistic, degrees of freedom (df), epsilon-squared effect size (ϵ^2) with 95% confidence intervals, and FDR-corrected p-values as a function of the window size (N). Each test compares 120 realizations, 30 for each of the four coupling level k.

single-layer DA					
**Series**	**Window size (N)**	**H Statistic**	**df**	**ϵ^2 [95% CI]**	$p_{\mathbf{fdr}}$
	30	1.12	3	-0.02 [-0.02, 0.09]	0.848
	60	1.94	3	-0.01 [-0.02, 0.10]	0.803
	120	2.36	3	-0.01 [-0.02, 0.10]	0.787
	240	2.97	3	-0.00 [-0.02, 0.11]	0.775
	480	4.32	3	0.01 [-0.02, 0.13]	0.775
x1	600	7.15	3	0.04 [-0.01, 0.17]	0.529
	720	6.34	3	0.03 [-0.01, 0.15]	0.529
	900	2.81	3	-0.00 [-0.02, 0.11]	0.775
	1200	2.80	3	-0.00 [-0.02, 0.11]	0.775
	2400	1.58	3	-0.01 [-0.02, 0.08]	0.812
	3600	0.80	3	-0.02 [-0.02, 0.07]	0.848
	30	1.83	3	-0.01 [-0.02, 0.08]	0.816
	60	0.48	3	-0.02 [-0.02, 0.07]	0.924
	120	1.72	3	-0.01 [-0.02, 0.08]	0.816
	240	2.01	3	-0.01 [-0.02, 0.09]	0.816
	480	2.72	3	-0.00 [-0.02, 0.11]	0.816
x2	600	4.04	3	0.01 [-0.02, 0.12]	0.816
	720	1.56	3	-0.01 [-0.02, 0.10]	0.816
	900	2.10	3	-0.01 [-0.02, 0.09]	0.816
	1200	0.87	3	-0.02 [-0.02, 0.08]	0.915
	2400	2.64	3	-0.00 [-0.02, 0.10]	0.816
	3600	2.33	3	-0.01 [-0.02, 0.10]	0.816
	30	0.36	3	-0.02 [-0.02, 0.07]	0.949
	60	1.78	3	-0.01 [-0.02, 0.08]	0.681
	120	2.11	3	-0.01 [-0.02, 0.09]	0.672
	240	5.20	3	0.02 [-0.02, 0.14]	0.348
	480	3.40	3	0.00 [-0.02, 0.13]	0.459
x3	600	9.88	3	0.06 [0.00, 0.18]	0.216
	720	8.07	3	0.04 [-0.01, 0.17]	0.242

continued on next page

Table S1 – *continued from previous page*

Series	Window size (N)	H Statistic	df	ϵ^2 [95% CI]	$p_{\mathbf{fdr}}$
	900	3.83	3	0.01 [-0.02, 0.13]	0.459
	1200	7.19	3	0.04 [-0.01, 0.17]	0.242
	2400	6.33	3	0.03 [-0.01, 0.17]	0.266
	3600	3.42	3	0.00 [-0.02, 0.11]	0.459
y1	30	63.01	3	0.52 [0.38, 0.66]	<0.001
	60	93.71	3	0.78 [0.69, 0.85]	<0.001
	120	106.48	3	0.89 [0.85, 0.91]	<0.001
	240	110.50	3	0.93 [0.90, 0.94]	<0.001
	480	111.58	3	0.94 [0.92, 0.94]	<0.001
	600	111.58	3	0.94 [0.92, 0.94]	<0.001
	720	111.58	3	0.94 [0.92, 0.94]	<0.001
	900	111.57	3	0.94 [0.92, 0.94]	<0.001
	1200	111.58	3	0.94 [0.92, 0.94]	<0.001
	2400	111.57	3	0.94 [0.92, 0.94]	<0.001
	3600	111.58	3	0.94 [0.92, 0.94]	<0.001
y2	30	44.39	3	0.36 [0.24, 0.52]	<0.001
	60	102.29	3	0.86 [0.80, 0.90]	<0.001
	120	108.87	3	0.91 [0.88, 0.93]	<0.001
	240	111.27	3	0.93 [0.92, 0.94]	<0.001
	480	111.58	3	0.94 [0.92, 0.94]	<0.001
	600	111.58	3	0.94 [0.92, 0.94]	<0.001
	720	111.58	3	0.94 [0.92, 0.94]	<0.001
	900	111.58	3	0.94 [0.92, 0.94]	<0.001
	1200	111.58	3	0.94 [0.92, 0.94]	<0.001
	2400	111.58	3	0.94 [0.92, 0.94]	<0.001
	3600	111.57	3	0.94 [0.92, 0.94]	<0.001
y3	30	59.50	3	0.49 [0.36, 0.62]	<0.001
	60	78.55	3	0.65 [0.54, 0.75]	<0.001
	120	80.36	3	0.67 [0.55, 0.77]	<0.001
	240	69.96	3	0.58 [0.46, 0.70]	<0.001
	480	70.49	3	0.58 [0.46, 0.70]	<0.001
	600	72.73	3	0.60 [0.48, 0.71]	<0.001
	720	77.08	3	0.64 [0.51, 0.74]	<0.001
	900	87.70	3	0.73 [0.63, 0.81]	<0.001
	1200	96.21	3	0.80 [0.73, 0.86]	<0.001
	2400	104.47	3	0.87 [0.82, 0.90]	<0.001
	3600	105.91	3	0.89 [0.84, 0.91]	<0.001

Table S2. Statistical results of the Kruskal-Wallis omnibus test for the duplex configuration (x_1, y_1) of the synthetic Rössler system. The table reports the H statistic, degrees of freedom (df), epsilon-squared effect size (ϵ^2) with 95% confidence intervals, and FDR-corrected p-values for both the multiplex Average Degree (DA) and the inter-layer Mutual Information (I) as a function of the window size (N). Each coupling level k has 30 realizations.

**Duplex (x1,y1)**					
**Measure**	**Window size (N)**	**H Statistic**	**df**	ϵ^2 $[95\%CI]$	$p_{\mathbf{fdr}}$
DA	30	6.14	3	0.03 [-0.01. 0.16]	0.122
	60	3.54	3	0.00 [-0.02, 0.12]	0.348
	120	8.67	3	0.05 [-0.00, 0.19]	0.044
	240	54.24	3	0.44 [0.31, 0.57]	<0.001
	480	71.61	3	0.59 [0.48, 0.71]	<0.001
	600	82.99	3	0.69 [0.59, 0.79]	<0.001
	720	79.35	3	0.66 [0.55, 0.75]	<0.001
	900	81.15	3	0.67 [0.56, 0.77]	<0.001
	1200	91.38	3	0.76 [0.67, 0.84]	<0.001
	2400	95.93	3	0.80 [0.72, 0.86]	<0.001
	3600	101.69	3	0.85 [0.79, 0.89]	<0.001
I	30	2.13	3	-0.01 [-0.02, 0.10]	0.559
	60	28.11	3	0.22 [0.10, 0.37]	<0.001
	120	28.84	3	0.22 [0.12, 0.37]	<0.001
	240	55.38	3	0.45 [0.33, 0.59]	<0.001
	480	78.52	3	0.65 [0.55, 0.75]	<0.001
	600	85.39	3	0.71 [0.61, 0.79]	<0.001
	720	85.35	3	0.71 [0.60, 0.79]	<0.001
	900	85.57	3	0.71 [0.59, 0.79]	<0.001
	1200	87.36	3	0.73 [0.63, 0.81]	<0.001
	2400	106.20	3	0.89 [0.85, 0.91]	<0.001
	3600	110.15	3	0.92 [0.90, 0.93]	<0.001

Table S3. Statistical results of the Kruskal-Wallis omnibus test for the 6-layer multiplex configuration $(x_1, x_2, x_3, y_1, y_2, y_3)$ of the synthetic Rössler system. The table reports the H statistic, degrees of freedom (df). epsilon-squared effect size (ϵ^2) with 95% confidence intervals, and FDR-corrected p-values for both the multiplex Average Degree (DA) and the inter-layer Mutual Information ($\bar{I}$) as a function of the window size (N). Each coupling level k has 30 realizations.

Multiplex (x1,x2,x3,y1,y2,y3)					
**Measure**	**Window size (N)**	**H Statistic**	**df**	ϵ^2 [95% CI]	$p_{\mathbf{fdr}}$
DA	30	1.81	3	-0.01 [-0.02. 0.09]	0.641
	60	9.34	3	0.05 [-0.01, 0.19]	0.029
	120	31.98	3	0.25 [0.11, 0.43]	<0.001
	240	84.27	3	0.70 [0.60, 0.79]	<0.001
	480	99.09	3	0.83 [0.75, 0.88]	<0.001
	600	103.98	3	0.87 [0.82, 0.90]	<0.001
	720	103.47	3	0.87 [0.80, 0.90]	<0.001
	900	105.12	3	0.88 [0.83, 0.91]	<0.001
	1200	108.06	3	0.91 [0.87, 0.92]	<0.001
	2400	110.59	3	0.93 [0.91, 0.93]	<0.001
	3600	111.47	3	0.94 [0.92, 0.94]	<0.001
$\bar{I}$	30	5.32	3	0.02 [-0.01, 0.15]	0.161
	60	58.19	3	0.48 [0.35, 0.60]	<0.001
	120	63.83	3	0.52 [0.39, 0.65]	<0.001
	240	82.37	3	0.68 [0.58, 0.78]	<0.001
	480	98.15	3	0.82 [0.73, 0.87]	<0.001
	600	105.54	3	0.88 [0.83, 0.91]	<0.001
	720	108.20	3	0.91 [0.87, 0.92]	<0.001
	900	108.01	3	0.91 [0.87, 0.92]	<0.001
	1200	109.67	3	0.92 [0.89, 0.93]	<0.001
	2400	109.54	3	0.92 [0.89, 0.93]	<0.001
	3600	111.32	3	0.93 [0.92, 0.94]	<0.001

Table S4. Statistical results of the Kruskal-Wallis omnibus test for the Mean Phase hoerence (MPC) of the synthetic Rössler system for two serie (x_1, y_1) and averaged across all pairs $(x_1, x_2, x_3, y_1, y_2, y_3)$. The table reports the H statistic, degrees of freedom (df), epsilon-squared effect size (ϵ^2) with 95% confidence intervals, and FDR-corrected p-values as a function of the window size (N). Each coupling level k has 30 realizations.

MPC					
**Series**	**Window (N)**	**H Statistic**	**df**	ϵ^2 $[95\%CI]$	$p_{\mathbf{fdr}}$
(x1,y1)	30	56.83	3	0.46 [0.32. 0.61]	<0.001
	60	68.39	3	0.56 [0.44, 0.67]	<0.001
	120	68.30	3	0.56 [0.44, 0.67]	<0.001
	240	80.39	3	0.67 [0.55, 0.77]	<0.001
	480	90.99	3	0.76 [0.66, 0.84]	<0.001
	600	96.46	3	0.81 [0.72, 0.86]	<0.001
	720	97.33	3	0.81 [0.74, 0.86]	<0.001
	900	101.20	3	0.85 [0.78, 0.89]	<0.001
	1200	104.06	3	0.87 [0.82, 0.90]	<0.001
	2400	110.04	3	0.92 [0.89, 0.93]	<0.001
	3600	111.57	3	0.94 [0.92, 0.94]	<0.001
(x1,x2,x3,y1,y2,y3)	30	58.69	3	0.48 [0.34, 0.60]	<0.001
	60	88.74	3	0.74 [0.62, 0.83]	<0.001
	120	94.44	3	0.79 [0.70, 0.85]	<0.001
	240	105.55	3	0.88 [0.83, 0.91]	<0.001
	480	107.79	3	0.90 [0.87, 0.93]	<0.001
	600	109.01	3	0.91 [0.88, 0.93]	<0.001
	720	110.41	3	0.93 [0.90, 0.93]	<0.001
	900	111.47	3	0.94 [0.92, 0.94]	<0.001
	1200	111.57	3	0.94 [0.92, 0.94]	<0.001
	2400	111.57	3	0.94 [0.92, 0.94]	<0.001
	3600	111.57	3	0.94 [0.92, 0.94]	<0.001

1.2. Post-Hoc Analyses

Table S5. Post-hoc pairwise comparisons (Mann-Whitney U test) for the single-layer Average Degree (DA) extracted from the x_1 state variable. The table reports the median and interquartile range (IQR) for each coupled group (k_1 to k_4, with 30 realizations each), the effect size (r) with 95% confidence intervals, and the FDR-corrected p-values across varying temporal window sizes (N).

DA - sigle layer (x1)					
**Window (N)**	**Comparison**	**Median (IQR) Group 1**	**Median (IQR) Group 2**	**Effect Size** r [95%CI]	$p_{\mathbf{fdr}}$
30	k_1 vs k_2	8.467 (8.317)	8.033 (8.733)	0.02 [0.00. 0.28]	0.952
	k_1 vs k_3	8.467 (8.317)	7.800 (11.033)	0.03 [0.00. 0.30]	0.921
	k_1 vs k_4	8.467 (8.317)	7.600 (14.933)	0.09 [0.01. 0.33]	0.921
	k_2 vs k_3	8.033 (8.733)	7.800 (11.033)	0.07 [0.00. 0.33]	0.921
	k_2 vs k_4	8.033 (8.733)	7.600 (14.933)	0.06 [0.01. 0.32]	0.921
	k_3 vs k_4	7.800 (11.033)	7.600 (14.933)	0.14 [0.01. 0.38]	0.897
60	k_1 vs k_2	19.317 (8.242)	23.300 (7.883)	0.18 [0.01. 0.40]	0.897
	k_1 vs k_3	19.317 (8.242)	23.650 (10.867)	0.04 [0.00. 0.30]	0.921
	k_1 vs k_4	19.317 (8.242)	19.917 (8.400)	0.01 [0.00. 0.30]	0.968
	k_2 vs k_3	23.300 (7.883)	23.650 (10.867)	0.06 [0.00. 0.34]	0.921
	k_2 vs k_4	23.300 (7.883)	19.917 (8.400)	0.16 [0.00. 0.40]	0.897
	k_3 vs k_4	23.650 (10.867)	19.917 (8.400)	0.06 [0.00. 0.33]	0.921
120	k_1 vs k_2	26.600 (6.337)	28.658 (5.471)	0.12 [0.01. 0.37]	0.897
	k_1 vs k_3	26.600 (6.337)	28.258 (4.104)	0.11 [0.00, 0.35]	0.921
	k_1 vs k_4	26.600 (6.337)	27.542 (6.100)	0.07 [0.00, 0.31]	0.921
	k_2 vs k_3	28.658 (5.471)	28.258 (4.104)	0.05 [0.00, 0.29]	0.921
	k_2 vs k_4	28.658 (5.471)	27.542 (6.100)	0.17 [0.01, 0.41]	0.897
	k_3 vs k_4	28.258 (4.104)	27.542 (6.100)	0.13 [0.01, 0.38]	0.897
240	k_1 vs k_2	29.987 (2.690)	29.029 (2.985)	0.06 [0.00, 0.31]	0.921
	k_1 vs k_3	29.987 (2.690)	30.471 (2.081)	0.13 [0.00, 0.38]	0.897
	k_1 vs k_4	29.987 (2.690)	29.871 (2.196)	0.07 [0.01, 0.33]	0.921
	k_2 vs k_3	29.029 (2.985)	30.471 (2.081)	0.22 [0.01, 0.46]	0.614
	k_2 vs k_4	29.029 (2.985)	29.871 (2.196)	0.14 [0.01, 0.37]	0.897
	k_3 vs k_4	30.471 (2.081)	29.871 (2.196)	0.08 [0.00, 0.34]	0.921
480	k_1 vs k_2	30.635 (1.362)	30.094 (0.925)	0.24 [0.03, 0.49]	0.614
	k_1 vs k_3	30.635 (1.362)	30.523 (1.527)	0.05 [0.00, 0.33]	0.921
	k_1 vs k_4	30.635 (1.362)	30.469 (1.439)	0.02 [0.00, 0.29]	0.952
	k_2 vs k_3	30.094 (0.925)	30.523 (1.527)	0.17 [0.01, 0.43]	0.897
	k_2 vs k_4	30.094 (0.925)	30.469 (1.439)	0.23 [0.02, 0.47]	0.614
	k_3 vs k_4	30.523 (1.527)	30.469 (1.439)	0.05 [0.00, 0.31]	0.921
600	k_1 vs k_2	30.548 (0.925)	30.217 (0.938)	0.30 [0.04, 0.53]	0.484
	k_1 vs k_3	30.548 (0.925)	30.535 (1.506)	0.01 [0.00, 0.29]	0.968
	k_1 vs k_4	30.548 (0.925)	30.488 (1.659)	0.02 [0.00, 0.30]	0.952
	k_2 vs k_3	30.217 (0.938)	30.535 (1.506)	0.30 [0.06, 0.50]	0.484
	k_2 vs k_4	30.217 (0.938)	30.488 (1.659)	0.24 [0.03, 0.49]	0.614
	k_3 vs k_4	30.535 (1.506)	30.488 (1.659)	0.03 [0.00, 0.30]	0.921
720	k_1 vs k_2	30.993 (1.741)	30.469 (0.746)	0.24 [0.03, 0.47]	0.614
	k_1 vs k_3	30.993 (1.741)	30.846 (1.283)	0.02 [0.00, 0.31]	0.952
	k_1 vs k_4	30.993 (1.741)	30.735 (1.853)	0.05 [0.01, 0.31]	0.921
	k_2 vs k_3	30.469 (0.746)	30.846 (1.283)	0.30 [0.07, 0.52]	0.484
	k_2 vs k_4	30.469 (0.746)	30.735 (1.853)	0.25 [0.03, 0.47]	0.614
	k_3 vs k_4	30.846 (1.283)	30.735 (1.853)	0.04 [0.00, 0.30]	0.921
900	k_1 vs k_2	31.323 (1.785)	30.833 (1.478)	0.13 [0.00, 0.38]	0.897
	k_1 vs k_3	31.323 (1.785)	31.269 (1.174)	0.01 [0.00, 0.30]	0.971
	k_1 vs k_4	31.323 (1.785)	31.417 (1.351)	0.06 [0.00, 0.32]	0.921
	k_2 vs k_3	30.833 (1.478)	31.269 (1.174)	0.14 [0.01, 0.39]	0.897
	k_2 vs k_4	30.833 (1.478)	31.417 (1.351)	0.21 [0.01, 0.44]	0.636
	k_3 vs k_4	31.269 (1.174)	31.417 (1.351)	0.08 [0.00, 0.34]	0.921
1200	k_1 vs k_2	31.385 (1.307)	31.197 (0.943)	0.08 [0.01, 0.34]	0.921
	k_1 vs k_3	31.385 (1.307)	31.473 (0.665)	0.12 [0.01. 0.37]	0.897
	k_1 vs k_4	31.385 (1.307)	31.385 (0.854)	0.04 [0.00. 0.31]	0.921
	k_2 vs k_3	31.197 (0.943)	31.473 (0.665)	0.22 [0.02. 0.47]	0.614
	k_2 vs k_4	31.197 (0.943)	31.385 (0.854)	0.12 [0.00. 0.36]	0.897
	k_3 vs k_4	31.473 (0.665)	31.385 (0.854)	0.09 [0.00. 0.34]	0.921
2400	k_1 vs k_2	31.591 (1.049)	31.490 (0.539)	0.12 [0.01. 0.34]	0.897
	k_1 vs k_3	31.591 (1.049)	31.525 (0.761)	0.05 [0.01, 0.32]	0.921
	k_1 vs k_4	31.591 (1.049)	31.626 (0.495)	0.01 [0.00, 0.29]	0.968
	k_2 vs k_3	31.490 (0.539)	31.525 (0.761)	0.10 [0.00, 0.33]	0.921
	k_2 vs k_4	31.490 (0.539)	31.626 (0.495)	0.16 [0.01, 0.40]	0.897
	k_3 vs k_4	31.525 (0.761)	31.626 (0.495)	0.04 [0.00, 0.34]	0.921
3600	k_1 vs k_2	31.829 (0.502)	31.843 (0.486)	0.07 [0.00, 0.33]	0.921
	k_1 vs k_3	31.829 (0.502)	31.876 (0.674)	0.03 [0.00, 0.30]	0.921
	k_1 vs k_4	31.829 (0.502)	31.800 (0.575)	0.12 [0.01, 0.39]	0.897
	k_2 vs k_3	31.843 (0.486)	31.876 (0.674)	0.04 [0.00, 0.31]	0.921
	k_2 vs k_4	31.843 (0.486)	31.800 (0.575)	0.04 [0.01, 0.30]	0.921
	k_3 vs k_4	31.876 (0.674)	31.800 (0.575)	0.07 [0.01, 0.32]	0.921

Table S6. Post-hoc pairwise comparisons (Mann-Whitney U test) for the single-layer Average Degree (DA) extracted from the x_2 state variable. The table reports the median and interquartile range (IQR) for each coupled group (k_1 to k_4), the effect size (r) with 95% confidence intervals, and the FDR-corrected p-values across varying temporal window sizes (N).

DA - sigle layer (x2)					
**Window (N)**	**Comparison**	**Median (IQR) Group 1**	**Median (IQR) Group 2**	**Effect Size** r [95%CI]	$p_{\mathbf{fdr}}$
	k_1 vs k_2	14.067 (12.583)	9.100 (13.850)	0.15 [0.01, 0.41]	0.952
30	k_1 vs k_3	14.067 (12.583)	9.700 (14.667)	0.09 [0.01, 0.38]	0.952
	k_1 vs k_4	14.067 (12.583)	9.967 (10.967)	0.16 [0.01, 0.41]	0.952
	k_2 vs k_3	9.100 (13.850)	9.700 (14.667)	0.05 [0.01, 0.29]	0.952
	k_2 vs k_4	9.100 (13.850)	9.967 (10.967)	0.01 [0.00, 0.29]	0.977
	k_3 vs k_4	9.700 (14.667)	9.967 (10.967)	0.04 [0.00, 0.30]	0.952
60	k_1 vs k_2	15.650 (9.083)	15.967 (8.442)	0.02 [0.01, 0.29]	0.977
	k_1 vs k_3	15.650 (9.083)	16.283 (8.458)	0.00 [0.00, 0.29]	0.991
	k_1 vs k_4	15.650 (9.083)	16.967 (10.767)	0.07 [0.01, 0.34]	0.952
	k_2 vs k_3	15.967 (8.442)	16.283 (8.458)	0.01 [0.00, 0.29]	0.977
	k_2 vs k_4	15.967 (8.442)	16.967 (10.767)	0.06 [0.00, 0.31]	0.952
	k_3 vs k_4	16.283 (8.458)	16.967 (10.767)	0.09 [0.01, 0.32]	0.952
120	k_1 vs k_2	23.550 (3.767)	24.192 (3.517)	0.03 [0.00, 0.29]	0.961
	k_1 vs k_3	23.550 (3.767)	23.383 (3.938)	0.12 [0.01, 0.36]	0.952
	k_1 vs k_4	23.550 (3.767)	24.250 (5.379)	0.04 [0.00, 0.28]	0.952
	k_2 vs k_3	24.192 (3.517)	23.383 (3.938)	0.15 [0.01, 0.38]	0.952
	k_2 vs k_4	24.192 (3.517)	24.250 (5.379)	0.02 [0.00, 0.31]	0.961
	k_3 vs k_4	23.383 (3.938)	24.250 (5.379)	0.13 [0.01, 0.39]	0.952
240	k_1 vs k_2	27.908 (1.967)	28.383 (1.910)	0.10 [0.00, 0.35]	0.952
	k_1 vs k_3	27.908 (1.967)	27.679 (1.785)	0.08 [0.01, 0.32]	0.952
	k_1 vs k_4	27.908 (1.967)	28.429 (2.760)	0.04 [0.00, 0.33]	0.952
	k_2 vs k_3	28.383 (1.910)	27.679 (1.785)	0.19 [0.02, 0.44]	0.952
	k_2 vs k_4	28.383 (1.910)	28.429 (2.760)	0.05 [0.00, 0.32]	0.952
	k_3 vs k_4	27.679 (1.785)	28.429 (2.760)	0.10 [0.00, 0.32]	0.952
480	k_1 vs k_2	30.337 (1.394)	30.917 (1.068)	0.22 [0.02, 0.45]	0.952
	k_1 vs k_3	30.337 (1.394)	30.517 (1.069)	0.07 [0.01, 0.35]	0.952
	k_1 vs k_4	30.337 (1.394)	30.494 (1.330)	0.08 [0.00, 0.32]	0.952
	k_2 vs k_3	30.917 (1.068)	30.517 (1.069)	0.14 [0.01, 0.40]	0.952
	k_2 vs k_4	30.917 (1.068)	30.494 (1.330)	0.11 [0.00, 0.35]	0.952
	k_3 vs k_4	30.517 (1.069)	30.494 (1.330)	0.00 [0.00, 0.30]	1.000
600	k_1 vs k_2	30.988 (1.558)	31.327 (1.128)	0.21 [0.02, 0.45]	0.952
	k_1 vs k_3	30.988 (1.558)	30.967 (0.827)	0.01 [0.00, 0.32]	0.977
	k_1 vs k_4	30.988 (1.558)	31.090 (0.928)	0.07 [0.01, 0.34]	0.952
	k_2 vs k_3	31.327 (1.128)	30.967 (0.827)	0.24 [0.02, 0.49]	0.952
	k_2 vs k_4	31.327 (1.128)	31.090 (0.928)	0.13 [0.00, 0.37]	0.952
	k_3 vs k_4	30.967 (0.827)	31.090 (0.928)	0.10 [0.01, 0.36]	0.952
720	k_1 vs k_2	31.067 (1.274)	31.483 (1.352)	0.14 [0.01, 0.38]	0.952
	k_1 vs k_3	31.067 (1.274)	31.210 (0.869)	0.10 [0.01, 0.35]	0.952
	k_1 vs k_4	31.067 (1.274)	31.257 (1.014)	0.10 [0.00, 0.35]	0.952
	k_2 vs k_3	31.483 (1.352)	31.210 (0.869)	0.09 [0.00, 0.34]	0.952
	k_2 vs k_4	31.483 (1.352)	31.257 (1.014)	0.06 [0.00, 0.30]	0.952
	k_3 vs k_4	31.210 (0.869)	31.257 (1.014)	0.03 [0.00, 0.30]	0.961
900	k_1 vs k_2	31.294 (1.027)	31.629 (1.121)	0.14 [0.01, 0.39]	0.952
	k_1 vs k_3	31.294 (1.027)	31.433 (0.641)	0.04 [0.00, 0.31]	0.952
	k_1 vs k_4	31.294 (1.027)	31.479 (1.033)	0.04 [0.00, 0.28]	0.952
	k_2 vs k_3	31.629 (1.121)	31.433 (0.641)	0.18 [0.01, 0.43]	0.952
	k_2 vs k_4	31.629 (1.121)	31.479 (1.033)	0.12 [0.01, 0.36]	0.952
	k_3 vs k_4	31.433 (0.641)	31.479 (1.033)	0.03 [0.01, 0.30]	0.961
1200	k_1 vs k_2	31.894 (0.660)	31.828 (0.555)	0.03 [0.00, 0.29]	0.955
	k_1 vs k_3	31.894 (0.660)	31.729 (0.640)	0.07 [0.00, 0.33]	0.952
	k_1 vs k_4	31.894 (0.660)	31.831 (0.557)	0.04 [0.00, 0.31]	0.952
	k_2 vs k_3	31.828 (0.555)	31.729 (0.640)	0.12 [0.01, 0.35]	0.952
	k_2 vs k_4	31.828 (0.555)	31.831 (0.557)	0.06 [0.00, 0.32]	0.952
	k_3 vs k_4	31.729 (0.640)	31.831 (0.557)	0.05 [0.00, 0.32]	0.952
2400	k_1 vs k_2	32.309 (0.547)	32.335 (0.435)	0.17 [0.01, 0.41]	0.952
	k_1 vs k_3	32.309 (0.547)	32.267 (0.523)	0.02 [0.00, 0.30]	0.970
	k_1 vs k_4	32.309 (0.547)	32.255 (0.357)	0.04 [0.01, 0.29]	0.952
	k_2 vs k_3	32.335 (0.435)	32.267 (0.523)	0.19 [0.01, 0.43]	0.952
	k_2 vs k_4	32.335 (0.435)	32.255 (0.357)	0.14 [0.00, 0.39]	0.952
	k_3 vs k_4	32.267 (0.523)	32.255 (0.357)	0.05 [0.00, 0.30]	0.952
3600	k_1 vs k_2	32.323 (0.200)	32.390 (0.267)	0.13 [0.01, 0.39]	0.952
	k_1 vs k_3	32.323 (0.200)	32.438 (0.409)	0.08 [0.00, 0.33]	0.952
	k_1 vs k_4	32.323 (0.200)	32.455 (0.307)	0.20 [0.01, 0.46]	0.952
	k_2 vs k_3	32.390 (0.267)	32.438 (0.409)	0.05 [0.00, 0.33]	0.952
	k_2 vs k_4	32.390 (0.267)	32.455 (0.307)	0.08 [0.01, 0.34]	0.952
	k_3 vs k_4	32.438 (0.409)	32.455 (0.307)	0.07 [0.01, 0.34]	0.952

Table S7. Post-hoc pairwise comparisons (Mann-Whitney U test) for the single-layer Average Degree (DA) extracted from the x_3 state variable. The table reports the median and interquartile range (IQR) for each coupled group (k_1 to k_4) with 30 realizations each, the effect size (r) with 95% confidence intervals, and the FDR-corrected p-values across varying temporal window sizes (N).

DA - sigle layer (x3)					
**Window (N)**	**Comparison**	**Median (IQR) Group 1**	**Median (IQR) Group 2**	**Effect Size** r [95%CI]	$p_{\mathbf{fdr}}$
30	k_1 vs k_2	15.633 (7.100)	16.200 (7.200)	0.06 [0.00, 0.32]	0.852
	k_1 vs k_3	15.633 (7.100)	16.100 (8.467)	0.00 [0.00, 0.31]	0.988
	k_1 vs k_4	15.633 (7.100)	19.433 (11.450)	0.03 [0.00, 0.31]	0.918
	k_2 vs k_3	16.200 (7.200)	16.100 (8.467)	0.07 [0.00, 0.32]	0.832
	k_2 vs k_4	16.200 (7.200)	19.433 (11.450)	0.01 [0.00, 0.30]	0.988
	k_3 vs k_4	16.100 (8.467)	19.433 (11.450)	0.05 [0.00, 0.32]	0.852
60	k_1 vs k_2	30.050 (7.417)	32.400 (7.717)	0.16 [0.01, 0.43]	0.573
	k_1 vs k_3	30.050 (7.417)	31.817 (10.042)	0.08 [0.00, 0.32]	0.781
	k_1 vs k_4	30.050 (7.417)	29.750 (7.742)	0.04 [0.01, 0.30]	0.898
	k_2 vs k_3	32.400 (7.717)	31.817 (10.042)	0.06 [0.00, 0.32]	0.852
	k_2 vs k_4	32.400 (7.717)	29.750 (7.742)	0.14 [0.01, 0.40]	0.631
	k_3 vs k_4	31.817 (10.042)	29.750 (7.742)	0.04 [0.00, 0.32]	0.881
120	k_1 vs k_2	39.342 (7.179)	40.433 (6.608)	0.14 [0.01, 0.40]	0.631
	k_1 vs k_3	39.342 (7.179)	40.633 (6.504)	0.13 [0.01, 0.37]	0.650
	k_1 vs k_4	39.342 (7.179)	39.108 (7.913)	0.01 [0.00, 0.29]	0.988
	k_2 vs k_3	40.433 (6.608)	40.633 (6.504)	0.03 [0.01, 0.29]	0.933
	k_2 vs k_4	40.433 (6.608)	39.108 (7.913)	0.12 [0.00, 0.38]	0.668
	k_3 vs k_4	40.633 (6.504)	39.108 (7.913)	0.13 [0.01, 0.37]	0.650
240	k_1 vs k_2	43.812 (2.917)	43.129 (3.560)	0.08 [0.00, 0.34]	0.781
	k_1 vs k_3	43.812 (2.917)	44.546 (2.377)	0.21 [0.02, 0.45]	0.524
	k_1 vs k_4	43.812 (2.917)	44.321 (2.767)	0.14 [0.01, 0.38]	0.631
	k_2 vs k_3	43.129 (3.560)	44.546 (2.377)	0.25 [0.02, 0.47]	0.389
	k_2 vs k_4	43.129 (3.560)	44.321 (2.767)	0.19 [0.02, 0.44]	0.528
	k_3 vs k_4	44.546 (2.377)	44.321 (2.767)	0.07 [0.00, 0.34]	0.799
480	k_1 vs k_2	45.673 (1.626)	44.858 (1.728)	0.19 [0.01, 0.44]	0.528
	k_1 vs k_3	45.673 (1.626)	46.054 (1.831)	0.02 [0.00, 0.30]	0.971
	k_1 vs k_4	45.673 (1.626)	45.590 (1.552)	0.06 [0.01, 0.32]	0.852
	k_2 vs k_3	44.858 (1.728)	46.054 (1.831)	0.20 [0.01, 0.46]	0.524
	k_2 vs k_4	44.858 (1.728)	45.590 (1.552)	0.16 [0.01, 0.40]	0.573
	k_3 vs k_4	46.054 (1.831)	45.590 (1.552)	0.09 [0.01, 0.35]	0.769
600	k_1 vs k_2	45.883 (1.885)	45.245 (1.399)	0.25 [0.03, 0.47]	0.389
	k_1 vs k_3	45.883 (1.885)	46.062 (1.118)	0.12 [0.00, 0.38]	0.700
	k_1 vs k_4	45.883 (1.885)	45.822 (1.662)	0.03 [0.00, 0.28]	0.918
	k_2 vs k_3	45.245 (1.399)	46.062 (1.118)	0.40 [0.16, 0.59]	0.132
	k_2 vs k_4	45.245 (1.399)	45.822 (1.662)	0.28 [0.04, 0.50]	0.389
	k_3 vs k_4	46.062 (1.118)	45.822 (1.662)	0.10 [0.01, 0.35]	0.755
720	k_1 vs k_2	45.997 (1.322)	45.665 (1.224)	0.23 [0.01, 0.46]	0.429
	k_1 vs k_3	45.997 (1.322)	46.461 (0.803)	0.10 [0.01, 0.33]	0.755
	k_1 vs k_4	45.997 (1.322)	46.324 (1.641)	0.05 [0.00, 0.30]	0.852
	k_2 vs k_3	45.665 (1.224)	46.461 (0.803)	0.35 [0.08, 0.56]	0.231
	k_2 vs k_4	45.665 (1.224)	46.324 (1.641)	0.28 [0.04, 0.49]	0.389
	k_3 vs k_4	46.461 (0.803)	46.324 (1.641)	0.08 [0.00, 0.34]	0.781
900	k_1 vs k_2	46.364 (1.811)	46.250 (1.563)	0.03 [0.00, 0.30]	0.933
	k_1 vs k_3	46.364 (1.811)	46.746 (1.427)	0.17 [0.01, 0.42]	0.549
	k_1 vs k_4	46.364 (1.811)	46.506 (1.576)	0.14 [0.01, 0.40]	0.631
	k_2 vs k_3	46.250 (1.563)	46.746 (1.427)	0.20 [0.02, 0.45]	0.524
	k_2 vs k_4	46.250 (1.563)	46.506 (1.576)	0.18 [0.02, 0.45]	0.528
	k_3 vs k_4	46.746 (1.427)	46.506 (1.576)	0.02 [0.00, 0.28]	0.962
1200	k_1 vs k_2	46.414 (1.348)	46.556 (1.159)	0.08 [0.00, 0.33]	0.781
	k_1 vs k_3	46.414 (1.348)	47.143 (1.020)	0.31 [0.07, 0.55]	0.330
	k_1 vs k_4	46.414 (1.348)	46.945 (1.128)	0.17 [0.01, 0.41]	0.549
	k_2 vs k_3	46.556 (1.159)	47.143 (1.020)	0.25 [0.03, 0.49]	0.389
	k_2 vs k_4	46.556 (1.159)	46.945 (1.128)	0.10 [0.00, 0.34]	0.755
	k_3 vs k_4	47.143 (1.020)	46.945 (1.128)	0.17 [0.01, 0.41]	0.551
2400	k_1 vs k_2	47.060 (1.245)	47.035 (0.958)	0.00 [0.00, 0.30]	0.988
	k_1 vs k_3	47.060 (1.245)	47.412 (0.611)	0.23 [0.01, 0.47]	0.429
	k_1 vs k_4	47.060 (1.245)	47.315 (0.593)	0.18 [0.01, 0.43]	0.528
	k_2 vs k_3	47.035 (0.958)	47.412 (0.611)	0.27 [0.03, 0.50]	0.389
	k_2 vs k_4	47.035 (0.958)	47.315 (0.593)	0.20 [0.01, 0.46]	0.528
	k_3 vs k_4	47.412 (0.611)	47.315 (0.593)	0.11 [0.01, 0.36]	0.715
3600	k_1 vs k_2	47.257 (0.467)	47.375 (0.645)	0.09 [0.01, 0.37]	0.769
	k_1 vs k_3	47.257 (0.467)	47.464 (0.520)	0.24 [0.02, 0.46]	0.403
	k_1 vs k_4	47.257 (0.467)	47.387 (0.564)	0.13 [0.00, 0.37]	0.666
	k_2 vs k_3	47.375 (0.645)	47.464 (0.520)	0.14 [0.01, 0.39]	0.631
	k_2 vs k_4	47.375 (0.645)	47.387 (0.564)	0.05 [0.00, 0.30]	0.857
	k_3 vs k_4	47.464 (0.520)	47.387 (0.564)	0.09 [0.00, 0.35]	0.779

Table S8. Post-hoc pairwise comparisons (Mann-Whitney U test) for the single-layer Average Degree (DA) extracted from the y_1 state variable. The table reports the median and interquartile range (IQR) for each coupled group (k_1 to k_4), the effect size (r) with 95% confidence intervals, and the FDR-corrected p-values across varying temporal window sizes (N).

DA - sigle layer (y1)					
**Window (N)**	**Comparison**	**Median (IQR) Group 1**	**Median (IQR) Group 2**	**Effect Size** r [95%CI]	$p_{\mathbf{fdr}}$
30	k_1 vs k_2	6.067 (0.567)	7.567 (0.467)	0.84 [0.78, 0.86]	<0.001
	k_1 vs k_3	6.067 (0.567)	6.600 (0.317)	0.69 [0.51, 0.81]	<0.001
	k_1 vs k_4	6.067 (0.567)	7.433 (1.367)	0.68 [0.53, 0.79]	<0.001
	k_2 vs k_3	7.567 (0.467)	6.600 (0.317)	0.66 [0.47, 0.81]	<0.001
	k_2 vs k_4	7.567 (0.467)	7.433 (1.367)	0.09 [0.00, 0.36]	<0.001
	k_3 vs k_4	6.600 (0.317)	7.433 (1.367)	0.33 [0.06, 0.59]	<0.001
60	k_1 vs k_2	6.717 (0.450)	7.833 (0.167)	0.86 [0.82, 0.86]	<0.001
	k_1 vs k_3	6.717 (0.450)	7.767 (0.417)	0.86 [0.83, 0.86]	<0.001
	k_1 vs k_4	6.717 (0.450)	8.600 (0.858)	0.86 [0.82, 0.86]	<0.001
	k_2 vs k_3	7.833 (0.167)	7.767 (0.417)	0.15 [0.01, 0.39]	<0.001
	k_2 vs k_4	7.833 (0.167)	8.600 (0.858)	0.78 [0.64, 0.85]	<0.001
	k_3 vs k_4	7.767 (0.417)	8.600 (0.858)	0.75 [0.61, 0.84]	<0.001
120	k_1 vs k_2	6.975 (0.308)	8.100 (0.204)	0.86 [0.82, 0.86]	<0.001
	k_1 vs k_3	6.975 (0.308)	8.342 (0.300)	0.86 [0.82, 0.86]	<0.001
	k_1 vs k_4	6.975 (0.308)	9.808 (1.367)	0.86 [0.82, 0.86]	<0.001
	k_2 vs k_3	8.100 (0.204)	8.342 (0.300)	0.64 [0.47, 0.77]	<0.001
	k_2 vs k_4	8.100 (0.204)	9.808 (1.367)	0.86 [0.82, 0.86]	<0.001
	k_3 vs k_4	8.342 (0.300)	9.808 (1.367)	0.85 [0.81, 0.86]	<0.001
240	k_1 vs k_2	7.142 (0.117)	8.283 (0.071)	0.86 [0.82, 0.86]	<0.001
	k_1 vs k_3	7.142 (0.117)	8.571 (0.198)	0.86 [0.82, 0.86]	<0.001
	k_1 vs k_4	7.142 (0.117)	11.458 (1.012)	0.86 [0.82, 0.86]	<0.001
	k_2 vs k_3	8.283 (0.071)	8.571 (0.198)	0.82 [0.71, 0.86]	<0.001
	k_2 vs k_4	8.283 (0.071)	11.458 (1.012)	0.86 [0.82, 0.86]	<0.001
	k_3 vs k_4	8.571 (0.198)	11.458 (1.012)	0.86 [0.83, 0.86]	<0.001
480	k_1 vs k_2	7.254 (0.093)	8.402 (0.082)	0.86 [0.82, 0.86]	<0.001
	k_1 vs k_3	7.254 (0.093)	8.771 (0.170)	0.86 [0.82, 0.86]	<0.001
	k_1 vs k_4	7.254 (0.093)	12.004 (0.690)	0.86 [0.82, 0.86]	<0.001
	k_2 vs k_3	8.402 (0.082)	8.771 (0.170)	0.86 [0.82, 0.86]	<0.001
	k_2 vs k_4	8.402 (0.082)	12.004 (0.690)	0.86 [0.83, 0.86]	<0.001
	k_3 vs k_4	8.771 (0.170)	12.004 (0.690)	0.86 [0.82, 0.86]	<0.001
600	k_1 vs k_2	7.272 (0.064)	8.412 (0.046)	0.86 [0.82, 0.86]	<0.001
	k_1 vs k_3	7.272 (0.064)	8.808 (0.257)	0.86 [0.83, 0.86]	<0.001
	k_1 vs k_4	7.272 (0.064)	12.083 (0.663)	0.86 [0.82, 0.86]	<0.001
	k_2 vs k_3	8.412 (0.046)	8.808 (0.257)	0.86 [0.82, 0.86]	<0.001
	k_2 vs k_4	8.412 (0.046)	12.083 (0.663)	0.86 [0.82, 0.86]	<0.001
	k_3 vs k_4	8.808 (0.257)	12.083 (0.663)	0.86 [0.82, 0.86]	<0.001
720	k_1 vs k_2	7.283 (0.053)	8.449 (0.047)	0.86 [0.82, 0.86]	<0.001
	k_1 vs k_3	7.283 (0.053)	8.843 (0.252)	0.86 [0.82, 0.86]	<0.001
	k_1 vs k_4	7.283 (0.053)	12.176 (0.495)	0.86 [0.83, 0.86]	<0.001
	k_2 vs k_3	8.449 (0.047)	8.843 (0.252)	0.86 [0.82, 0.86]	<0.001
	k_2 vs k_4	8.449 (0.047)	12.176 (0.495)	0.86 [0.82, 0.86]	<0.001
	k_3 vs k_4	8.843 (0.252)	12.176 (0.495)	0.86 [0.82, 0.86]	<0.001
900	k_1 vs k_2	7.303 (0.051)	8.458 (0.041)	0.86 [0.82, 0.86]	<0.001
	k_1 vs k_3	7.303 (0.051)	8.876 (0.229)	0.86 [0.83, 0.86]	<0.001
	k_1 vs k_4	7.303 (0.051)	12.208 (0.484)	0.86 [0.82, 0.86]	<0.001
	k_2 vs k_3	8.458 (0.041)	8.876 (0.229)	0.86 [0.82, 0.86]	<0.001
	k_2 vs k_4	8.458 (0.041)	12.208 (0.484)	0.86 [0.82, 0.86]	<0.001
	k_3 vs k_4	8.876 (0.229)	12.208 (0.484)	0.86 [0.83, 0.86]	<0.001
1200	k_1 vs k_2	7.323 (0.061)	8.471 (0.037)	0.86 [0.82, 0.86]	<0.001
	k_1 vs k_3	7.323 (0.061)	8.930 (0.184)	0.86 [0.83, 0.86]	<0.001
	k_1 vs k_4	7.323 (0.061)	12.224 (0.549)	0.86 [0.83, 0.86]	<0.001
	k_2 vs k_3	8.471 (0.037)	8.930 (0.184)	0.86 [0.83, 0.86]	<0.001
	k_2 vs k_4	8.471 (0.037)	12.224 (0.549)	0.86 [0.82, 0.86]	<0.001
	k_3 vs k_4	8.930 (0.184)	12.224 (0.549)	0.86 [0.83, 0.86]	<0.001
2400	k_1 vs k_2	7.353 (0.046)	8.498 (0.037)	0.86 [0.82, 0.86]	<0.001
	k_1 vs k_3	7.353 (0.046)	8.991 (0.102)	0.86 [0.82, 0.86]	<0.001
	k_1 vs k_4	7.353 (0.046)	12.386 (0.284)	0.86 [0.82, 0.86]	<0.001
	k_2 vs k_3	8.498 (0.037)	8.991 (0.102)	0.86 [0.83, 0.86]	<0.001
	k_2 vs k_4	8.498 (0.037)	12.386 (0.284)	0.86 [0.82, 0.86]	<0.001
	k_3 vs k_4	8.991 (0.102)	12.386 (0.284)	0.86 [0.82, 0.86]	<0.001
3600	k_1 vs k_2	7.353 (0.040)	8.508 (0.014)	0.86 [0.83, 0.86]	<0.001
	k_1 vs k_3	7.353 (0.040)	9.015 (0.133)	0.86 [0.83, 0.86]	<0.001
	k_1 vs k_4	7.353 (0.040)	12.383 (0.355)	0.86 [0.83, 0.86]	<0.001
	k_2 vs k_3	8.508 (0.014)	9.015 (0.133)	0.86 [0.83, 0.86]	<0.001
	k_2 vs k_4	8.508 (0.014)	12.383 (0.355)	0.86 [0.82, 0.86]	<0.001
	k_3 vs k_4	9.015 (0.133)	12.383 (0.355)	0.86 [0.82, 0.86]	<0.001

Table S9. Post-hoc pairwise comparisons (Mann-Whitney U test) for the single-layer Average Degree (DA) extracted from the y_2 state variable. The table reports the median and interquartile range (IQR) for each coupled group (k_1 to k_4) with 30 realizations each, the effect size (r) with 95% confidence intervals, and the FDR-corrected p-values across varying temporal window sizes (N).

DA - sigle layer (y2)					
**Window (N)**	**Comparison**	**Median (IQR) Group 1**	**Median (IQR) Group 2**	**Effect Size** r [95%CI]	$p_{\mathbf{fdr}}$
30	k_1 vs k_2	6.467 (0.367)	6.567 (0.583)	0.11 [0.01, 0.36]	<0.001
	k_1 vs k_3	6.467 (0.367)	7.467 (0.600)	0.79 [0.68, 0.84]	<0.001
	k_1 vs k_4	6.467 (0.367)	7.267 (5.500)	0.51 [0.27, 0.71]	<0.001
	k_2 vs k_3	6.567 (0.583)	7.467 (0.600)	0.65 [0.48, 0.79]	<0.001
	k_2 vs k_4	6.567 (0.583)	7.267 (5.500)	0.44 [0.20, 0.64]	<0.001
	k_3 vs k_4	7.467 (0.600)	7.267 (5.500)	0.03 [0.00, 0.31]	<0.001
60	k_1 vs k_2	7.300 (0.158)	7.633 (0.267)	0.65 [0.46, 0.78]	<0.001
	k_1 vs k_3	7.300 (0.158)	8.217 (0.425)	0.86 [0.82, 0.86]	<0.001
	k_1 vs k_4	7.300 (0.158)	13.017 (6.525)	0.86 [0.82, 0.86]	<0.001
	k_2 vs k_3	7.633 (0.267)	8.217 (0.425)	0.76 [0.65, 0.84]	<0.001
	k_2 vs k_4	7.633 (0.267)	13.017 (6.525)	0.86 [0.81, 0.86]	<0.001
	k_3 vs k_4	8.217 (0.425)	13.017 (6.525)	0.81 [0.69, 0.86]	<0.001
120	k_1 vs k_2	7.692 (0.142)	7.950 (0.150)	0.75 [0.63, 0.82]	<0.001
	k_1 vs k_3	7.692 (0.142)	8.550 (0.379)	0.86 [0.82, 0.86]	<0.001
	k_1 vs k_4	7.692 (0.142)	17.508 (3.721)	0.86 [0.83, 0.86]	<0.001
	k_2 vs k_3	7.950 (0.150)	8.550 (0.379)	0.86 [0.83, 0.86]	<0.001
	k_2 vs k_4	7.950 (0.150)	17.508 (3.721)	0.86 [0.82, 0.86]	<0.001
	k_3 vs k_4	8.550 (0.379)	17.508 (3.721)	0.86 [0.83, 0.86]	<0.001
240	k_1 vs k_2	7.942 (0.067)	8.250 (0.100)	0.85 [0.80, 0.86]	<0.001
	k_1 vs k_3	7.942 (0.067)	8.850 (0.283)	0.86 [0.82, 0.86]	<0.001
	k_1 vs k_4	7.942 (0.067)	19.879 (1.823)	0.86 [0.83, 0.86]	<0.001
	k_2 vs k_3	8.250 (0.100)	8.850 (0.283)	0.86 [0.82, 0.86]	<0.001
	k_2 vs k_4	8.250 (0.100)	19.879 (1.823)	0.86 [0.82, 0.86]	<0.001
	k_3 vs k_4	8.850 (0.283)	19.879 (1.823)	0.86 [0.82, 0.86]	<0.001
480	k_1 vs k_2	8.077 (0.073)	8.367 (0.068)	0.86 [0.83, 0.86]	<0.001
	k_1 vs k_3	8.077 (0.073)	9.010 (0.117)	0.86 [0.82, 0.86]	<0.001
	k_1 vs k_4	8.077 (0.073)	21.079 (0.635)	0.86 [0.82, 0.86]	<0.001
	k_2 vs k_3	8.367 (0.068)	9.010 (0.117)	0.86 [0.82, 0.86]	<0.001
	k_2 vs k_4	8.367 (0.068)	21.079 (0.635)	0.86 [0.82, 0.86]	<0.001
	k_3 vs k_4	9.010 (0.117)	21.079 (0.635)	0.86 [0.83, 0.86]	<0.001
600	k_1 vs k_2	8.105 (0.070)	8.405 (0.076)	0.86 [0.83, 0.86]	<0.001
	k_1 vs k_3	8.105 (0.070)	9.070 (0.151)	0.86 [0.83, 0.86]	<0.001
	k_1 vs k_4	8.105 (0.070)	21.192 (1.173)	0.86 [0.82, 0.86]	<0.001
	k_2 vs k_3	8.405 (0.076)	9.070 (0.151)	0.86 [0.83, 0.86]	<0.001
	k_2 vs k_4	8.405 (0.076)	21.192 (1.173)	0.86 [0.82, 0.86]	<0.001
	k_3 vs k_4	9.070 (0.151)	21.192 (1.173)	0.86 [0.83, 0.86]	<0.001
720	k_1 vs k_2	8.125 (0.049)	8.421 (0.057)	0.86 [0.82, 0.86]	<0.001
	k_1 vs k_3	8.125 (0.049)	9.067 (0.190)	0.86 [0.83, 0.86]	<0.001
	k_1 vs k_4	8.125 (0.049)	21.337 (0.524)	0.86 [0.82, 0.86]	<0.001
	k_2 vs k_3	8.421 (0.057)	9.067 (0.190)	0.86 [0.82, 0.86]	<0.001
	k_2 vs k_4	8.421 (0.057)	21.337 (0.524)	0.86 [0.82, 0.86]	<0.001
	k_3 vs k_4	9.067 (0.190)	21.337 (0.524)	0.86 [0.83, 0.86]	<0.001
900	k_1 vs k_2	8.147 (0.043)	8.450 (0.048)	0.86 [0.82, 0.86]	<0.001
	k_1 vs k_3	8.147 (0.043)	9.101 (0.190)	0.86 [0.82, 0.86]	<0.001
	k_1 vs k_4	8.147 (0.043)	21.493 (0.786)	0.86 [0.83, 0.86]	<0.001
	k_2 vs k_3	8.450 (0.048)	9.101 (0.190)	0.86 [0.82, 0.86]	<0.001
	k_2 vs k_4	8.450 (0.048)	21.493 (0.786)	0.86 [0.82, 0.86]	<0.001
	k_3 vs k_4	9.101 (0.190)	21.493 (0.786)	0.86 [0.83, 0.86]	<0.001
1200	k_1 vs k_2	8.167 (0.033)	8.462 (0.040)	0.86 [0.83, 0.86]	<0.001
	k_1 vs k_3	8.167 (0.033)	9.171 (0.139)	0.86 [0.82, 0.86]	<0.001
	k_1 vs k_4	8.167 (0.033)	21.606 (0.608)	0.86 [0.82, 0.86]	<0.001
	k_2 vs k_3	8.462 (0.040)	9.171 (0.139)	0.86 [0.83, 0.86]	<0.001
	k_2 vs k_4	8.462 (0.040)	21.606 (0.608)	0.86 [0.82, 0.86]	<0.001
	k_3 vs k_4	9.171 (0.139)	21.606 (0.608)	0.86 [0.83, 0.86]	<0.001
2400	k_1 vs k_2	8.202 (0.028)	8.504 (0.039)	0.86 [0.82, 0.86]	<0.001
	k_1 vs k_3	8.202 (0.028)	9.229 (0.096)	0.86 [0.83, 0.86]	<0.001
	k_1 vs k_4	8.202 (0.028)	21.761 (0.388)	0.86 [0.81, 0.86]	<0.001
	k_2 vs k_3	8.504 (0.039)	9.229 (0.096)	0.86 [0.82, 0.86]	<0.001
	k_2 vs k_4	8.504 (0.039)	21.761 (0.388)	0.86 [0.82, 0.86]	<0.001
	k_3 vs k_4	9.229 (0.096)	21.761 (0.388)	0.86 [0.82, 0.86]	<0.001
3600	k_1 vs k_2	8.210 (0.030)	8.520 (0.030)	0.86 [0.82, 0.86]	<0.001
	k_1 vs k_3	8.210 (0.030)	9.234 (0.098)	0.86 [0.82, 0.86]	<0.001
	k_1 vs k_4	8.210 (0.030)	21.869 (0.351)	0.86 [0.82, 0.86]	<0.001
	k_2 vs k_3	8.520 (0.030)	9.234 (0.098)	0.86 [0.82, 0.86]	<0.001
	k_2 vs k_4	8.520 (0.030)	21.869 (0.351)	0.86 [0.82, 0.86]	<0.001
	k_3 vs k_4	9.234 (0.098)	21.869 (0.351)	0.86 [0.82, 0.86]	<0.001

Table S10. Post-hoc pairwise comparisons (Mann-Whitney U test) for the single-layer Average Degree (DA) extracted from the y_3 state variable. The table reports the median and interquartile range (IQR) for each coupled group (k_1 to k_4), the effect size (r) with 95% confidence intervals, and the FDR-corrected p-values across varying temporal window sizes (N).

DA - sigle layer (y3)					
**Window (N)**	**Comparison**	**Median (IQR) Group 1**	**Median (IQR) Group 2**	**Effect Size** r [95%CI]	$p_{\mathbf{fdr}}$
30	k_1 vs k_2	8.267 (1.200)	9.867 (1.167)	0.82 [0.74, 0.85]	<0.001
	k_1 vs k_3	8.267 (1.200)	9.367 (0.750)	0.61 [0.43, 0.75]	<0.001
	k_1 vs k_4	8.267 (1.200)	8.733 (2.400)	0.02 [0.01, 0.32]	0.894
	k_2 vs k_3	9.867 (1.167)	9.367 (0.750)	0.44 [0.21, 0.64]	<0.001
	k_2 vs k_4	9.867 (1.167)	8.733 (2.400)	0.74 [0.60, 0.83]	<0.001
	k_3 vs k_4	9.367 (0.750)	8.733 (2.400)	0.47 [0.27, 0.65]	<0.001
60	k_1 vs k_2	9.333 (0.625)	11.517 (0.983)	0.86 [0.83, 0.86]	<0.001
	k_1 vs k_3	9.333 (0.625)	10.900 (0.925)	0.86 [0.81, 0.86]	<0.001
	k_1 vs k_4	9.333 (0.625)	9.750 (1.233)	0.26 [0.03, 0.50]	0.052
	k_2 vs k_3	11.517 (0.983)	10.900 (0.925)	0.57 [0.35, 0.73]	<0.001
	k_2 vs k_4	11.517 (0.983)	9.750 (1.233)	0.72 [0.53, 0.85]	<0.001
	k_3 vs k_4	10.900 (0.925)	9.750 (1.233)	0.58 [0.40, 0.75]	<0.001
120	k_1 vs k_2	9.908 (0.504)	12.125 (0.508)	0.86 [0.82, 0.86]	<0.001
	k_1 vs k_3	9.908 (0.504)	11.608 (0.950)	0.86 [0.82, 0.86]	<0.001
	k_1 vs k_4	9.908 (0.504)	10.792 (0.775)	0.74 [0.60, 0.83]	<0.001
	k_2 vs k_3	12.125 (0.508)	11.608 (0.950)	0.29 [0.04, 0.52]	0.028
	k_2 vs k_4	12.125 (0.508)	10.792 (0.775)	0.63 [0.40, 0.80]	<0.001
	k_3 vs k_4	11.608 (0.950)	10.792 (0.775)	0.55 [0.31, 0.74]	<0.001
240	k_1 vs k_2	10.250 (0.233)	12.650 (0.396)	0.86 [0.82, 0.86]	<0.001
	k_1 vs k_3	10.250 (0.233)	12.675 (1.410)	0.86 [0.82, 0.86]	<0.001
	k_1 vs k_4	10.250 (0.233)	12.129 (1.198)	0.86 [0.82, 0.86]	<0.001
	k_2 vs k_3	12.650 (0.396)	12.675 (1.410)	0.04 [0.00, 0.34]	<0.001
	k_2 vs k_4	12.650 (0.396)	12.129 (1.198)	0.29 [0.05, 0.55]	0.027
	k_3 vs k_4	12.675 (1.410)	12.129 (1.198)	0.24 [0.03, 0.47]	0.073
480	k_1 vs k_2	10.448 (0.216)	12.831 (0.436)	0.86 [0.82, 0.86]	<0.001
	k_1 vs k_3	10.448 (0.216)	13.148 (1.191)	0.86 [0.82, 0.86]	<0.001
	k_1 vs k_4	10.448 (0.216)	12.900 (0.718)	0.86 [0.83, 0.86]	<0.001
	k_2 vs k_3	12.831 (0.436)	13.148 (1.191)	0.29 [0.05, 0.53]	0.027
	k_2 vs k_4	12.831 (0.436)	12.900 (0.718)	0.05 [0.00, 0.33]	0.734
	k_3 vs k_4	13.148 (1.191)	12.900 (0.718)	0.26 [0.03, 0.49]	0.049
600	k_1 vs k_2	10.530 (0.200)	12.837 (0.200)	0.86 [0.82, 0.86]	<0.001
	k_1 vs k_3	10.530 (0.200)	13.443 (1.257)	0.86 [0.82, 0.86]	<0.001
	k_1 vs k_4	10.530 (0.200)	12.885 (0.659)	0.86 [0.82, 0.86]	<0.001
	k_2 vs k_3	12.837 (0.200)	13.443 (1.257)	0.37 [0.12, 0.58]	0.006
	k_2 vs k_4	12.837 (0.200)	12.885 (0.659)	0.08 [0.01, 0.35]	0.539
	k_3 vs k_4	13.443 (1.257)	12.885 (0.659)	0.34 [0.12, 0.56]	0.011
720	k_1 vs k_2	10.500 (0.144)	12.893 (0.249)	0.86 [0.82, 0.86]	<0.001
	k_1 vs k_3	10.500 (0.144)	13.436 (1.152)	0.86 [0.82, 0.86]	<0.001
	k_1 vs k_4	10.500 (0.144)	13.061 (0.485)	0.86 [0.82, 0.86]	<0.001
	k_2 vs k_3	12.893 (0.249)	13.436 (1.152)	0.51 [0.29, 0.69]	<0.001
	k_2 vs k_4	12.893 (0.249)	13.061 (0.485)	0.22 [0.02, 0.47]	0.099
	k_3 vs k_4	13.436 (1.152)	13.061 (0.485)	0.37 [0.13, 0.58]	0.005
900	k_1 vs k_2	10.512 (0.197)	12.907 (0.204)	0.86 [0.83, 0.86]	<0.001
	k_1 vs k_3	10.512 (0.197)	13.731 (1.006)	0.86 [0.82, 0.86]	<0.001
	k_1 vs k_4	10.512 (0.197)	13.122 (0.583)	0.86 [0.82, 0.86]	<0.001
	k_2 vs k_3	12.907 (0.204)	13.731 (1.006)	0.76 [0.62, 0.84]	<0.001
	k_2 vs k_4	12.907 (0.204)	13.122 (0.583)	0.33 [0.08, 0.57]	0.014
	k_3 vs k_4	13.731 (1.006)	13.122 (0.583)	0.48 [0.27, 0.67]	<0.001
1200	k_1 vs k_2	10.541 (0.129)	12.920 (0.196)	0.86 [0.83, 0.86]	<0.001
	k_1 vs k_3	10.541 (0.129)	13.855 (0.691)	0.86 [0.82, 0.86]	<0.001
	k_1 vs k_4	10.541 (0.129)	13.136 (0.427)	0.86 [0.82, 0.86]	<0.001
	k_2 vs k_3	12.920 (0.196)	13.855 (0.691)	0.83 [0.76, 0.86]	<0.001
	k_2 vs k_4	12.920 (0.196)	13.136 (0.427)	0.42 [0.18, 0.62]	0.001
	k_3 vs k_4	13.855 (0.691)	13.136 (0.427)	0.67 [0.50, 0.78]	<0.001
2400	k_1 vs k_2	10.609 (0.084)	12.977 (0.088)	0.86 [0.82, 0.86]	<0.001
	k_1 vs k_3	10.609 (0.084)	14.087 (0.900)	0.86 [0.82, 0.86]	<0.001
	k_1 vs k_4	10.609 (0.084)	13.186 (0.303)	0.86 [0.82, 0.86]	<0.001
	k_2 vs k_3	12.977 (0.088)	14.087 (0.900)	0.86 [0.82, 0.86]	<0.001
	k_2 vs k_4	12.977 (0.088)	13.186 (0.303)	0.58 [0.36, 0.75]	<0.001
	k_3 vs k_4	14.087 (0.900)	13.186 (0.303)	0.82 [0.75, 0.86]	<0.001
3600	k_1 vs k_2	10.629 (0.062)	12.982 (0.102)	0.86 [0.82, 0.86]	<0.001
	k_1 vs k_3	10.629 (0.062)	14.235 (0.775)	0.86 [0.83, 0.86]	<0.001
	k_1 vs k_4	10.629 (0.062)	13.211 (0.230)	0.86 [0.82, 0.86]	<0.001
	k_2 vs k_3	12.982 (0.102)	14.235 (0.775)	0.86 [0.83, 0.86]	<0.001
	k_2 vs k_4	12.982 (0.102)	13.211 (0.230)	0.62 [0.44, 0.77]	<0.001
	k_3 vs k_4	14.235 (0.775)	13.211 (0.230)	0.85 [0.80, 0.86]	<0.001

Table S11. Post-hoc pairwise comparisons (Mann-Whitney U test) for the Average Degree (DA) of duplex (x_1, y_1) configuration across varying simulated coupling strengths (k_1 to k_4). The table details the median and interquartile range (IQR) for each group, the effect size (r) with 95% confidence intervals, and the FDR-corrected p-values across different window lengths (N).

**DA - duplex (x1.y1)**					
**Window (N)**	**Comparison**	**Median (IQR) Group 1**	**Median (IQR) Group 2**	**Effect Size** r [95%CI]	$p_{\mathbf{fdr}}$
30	k_1 vs k_2	8.200 (3.758)	8.683 (4.117)	0.18 [0.01. 0.41]	0.218
	k_1 vs k_3	8.200 (3.758)	8.450 (5.442)	0.05 [0.00, 0.31]	0.789
	k_1 vs k_4	8.200 (3.758)	8.567 (6.858)	0.27 [0.03, 0.49]	0.058
	k_2 vs k_3	8.683 (4.117)	8.450 (5.442)	0.16 [0.01, 0.39]	0.297
	k_2 vs k_4	8.683 (4.117)	8.567 (6.858)	0.09 [0.01, 0.34]	0.565
	k_3 vs k_4	8.450 (5.442)	8.567 (6.858)	0.25 [0.03, 0.48]	0.078
60	k_1 vs k_2	14.067 (4.317)	16.500 (3.921)	0.24 [0.03, 0.47]	0.090
	k_1 vs k_3	14.067 (4.317)	16.658 (5.183)	0.13 [0.01, 0.39]	0.400
	k_1 vs k_4	14.067 (4.317)	15.558 (3.954)	0.18 [0.01, 0.41]	0.228
	k_2 vs k_3	16.500 (3.921)	16.658 (5.183)	0.08 [0.01, 0.33]	0.650
	k_2 vs k_4	16.500 (3.921)	15.558 (3.954)	0.04 [0.00, 0.30]	0.798
	k_3 vs k_4	16.658 (5.183)	15.558 (3.954)	0.05 [0.00, 0.31]	0.771
120	k_1 vs k_2	17.896 (3.198)	19.346 (2.742)	0.25 [0.03, 0.48]	0.078
	k_1 vs k_3	17.896 (3.198)	19.275 (1.923)	0.27 [0.03, 0.49]	0.060
	k_1 vs k_4	17.896 (3.198)	19.858 (3.229)	0.35 [0.13, 0.56]	0.0119
	k_2 vs k_3	19.346 (2.742)	19.275 (1.923)	0.00 [0.00, 0.28]	1.000
	k_2 vs k_4	19.346 (2.742)	19.858 (3.229)	0.13 [0.01, 0.39]	0.4037
	k_3 vs k_4	19.275 (1.923)	19.858 (3.229)	0.12 [0.00, 0.37]	0.462
240	k_1 vs k_2	19.560 (1.212)	19.633 (1.525)	0.16 [0.01, 0.40]	0.297
	k_1 vs k_3	19.560 (1.212)	20.523 (1.028)	0.46 [0.22, 0.65]	<0.001
	k_1 vs k_4	19.560 (1.212)	21.602 (1.368)	0.79 [0.69, 0.84]	<0.001
	k_2 vs k_3	19.633 (1.525)	20.523 (1.028)	0.30 [0.06, 0.53]	0.0334
	k_2 vs k_4	19.633 (1.525)	21.602 (1.368)	0.72 [0.58, 0.82]	<0.001
	k_3 vs k_4	20.523 (1.028)	21.602 (1.368)	0.59 [0.40, 0.74]	<0.001
480	k_1 vs k_2	19.996 (0.676)	20.234 (0.504)	0.29 [0.06, 0.52]	0.036
	k_1 vs k_3	19.996 (0.676)	20.758 (0.831)	0.55 [0.36, 0.71]	<0.001
	k_1 vs k_4	19.996 (0.676)	22.365 (0.884)	0.84 [0.79, 0.86]	<0.001
	k_2 vs k_3	20.234 (0.504)	20.758 (0.831)	0.34 [0.10, 0.54]	0.0149
	k_2 vs k_4	20.234 (0.504)	22.365 (0.884)	0.82 [0.75, 0.85]	<0.001
	k_3 vs k_4	20.758 (0.831)	22.365 (0.884)	0.77 [0.64, 0.85]	<0.001
600	k_1 vs k_2	19.892 (0.488)	20.323 (0.430)	0.34 [0.10, 0.56]	0.0149
	k_1 vs k_3	19.892 (0.488)	20.743 (0.730)	0.67 [0.50, 0.80]	<0.001
	k_1 vs k_4	19.892 (0.488)	22.348 (1.034)	0.86 [0.81, 0.86]	<0.001
	k_2 vs k_3	20.323 (0.430)	20.743 (0.730)	0.49 [0.26, 0.68]	<0.001
	k_2 vs k_4	20.323 (0.430)	22.348 (1.034)	0.85 [0.81, 0.86]	<0.001
	k_3 vs k_4	20.743 (0.730)	22.348 (1.034)	0.82 [0.75, 0.85]	<0.001
720	k_1 vs k_2	20.160 (0.883)	20.435 (0.383)	0.23 [0.02, 0.47]	0.115
	k_1 vs k_3	20.160 (0.883)	20.937 (0.604)	0.57 [0.38, 0.72]	<0.001
	k_1 vs k_4	20.160 (0.883)	22.495 (1.048)	0.86 [0.82, 0.86]	<0.001
	k_2 vs k_3	20.435 (0.383)	20.937 (0.604)	0.48 [0.25, 0.67]	<0.001
	k_2 vs k_4	20.435 (0.383)	22.495 (1.048)	0.86 [0.82, 0.86]	<0.001
	k_3 vs k_4	20.937 (0.604)	22.495 (1.048)	0.82 [0.75, 0.86]	<0.001
900	k_1 vs k_2	20.327 (0.908)	20.645 (0.742)	0.36 [0.13, 0.58]	0.008
	k_1 vs k_3	20.327 (0.908)	21.093 (0.557)	0.63 [0.46, 0.75]	<0.001
	k_1 vs k_4	20.327 (0.908)	22.792 (0.771)	0.86 [0.82, 0.86]	<0.001
	k_2 vs k_3	20.645 (0.742)	21.093 (0.557)	0.34 [0.11, 0.55]	0.013
	k_2 vs k_4	20.645 (0.742)	22.792 (0.771)	0.86 [0.83, 0.86]	<0.001
	k_3 vs k_4	21.093 (0.557)	22.792 (0.771)	0.85 [0.80, 0.86]	<0.001
1200	k_1 vs k_2	20.370 (0.660)	20.830 (0.453)	0.55 [0.36, 0.70]	<0.001
	k_1 vs k_3	20.370 (0.660)	21.191 (0.415)	0.76 [0.63, 0.84]	<0.001
	k_1 vs k_4	20.370 (0.660)	22.807 (0.612)	0.86 [0.82, 0.86]	<0.001
	k_2 vs k_3	20.830 (0.453)	21.191 (0.415)	0.47 [0.26, 0.66]	<0.001
	k_2 vs k_4	20.830 (0.453)	22.807 (0.612)	0.86 [0.82, 0.86]	<0.001
	k_3 vs k_4	21.191 (0.415)	22.807 (0.612)	0.86 [0.82, 0.86]	<0.001
2400	k_1 vs k_2	20.456 (0.541)	20.984 (0.279)	0.60 [0.42, 0.74]	<0.001
	k_1 vs k_3	20.456 (0.541)	21.280 (0.383)	0.81 [0.73, 0.85]	<0.001
	k_1 vs k_4	20.456 (0.541)	23.084 (0.323)	0.86 [0.82, 0.86]	<0.001
	k_2 vs k_3	20.984 (0.279)	21.280 (0.383)	0.55 [0.36, 0.73]	<0.001
	k_2 vs k_4	20.984 (0.279)	23.084 (0.323)	0.86 [0.82, 0.86]	<0.001
	k_3 vs k_4	21.280 (0.383)	23.084 (0.323)	0.86 [0.82, 0.86]	<0.001
3600	k_1 vs k_2	20.608 (0.232)	21.177 (0.242)	0.77 [0.67, 0.84]	<0.001
	k_1 vs k_3	20.608 (0.232)	21.448 (0.335)	0.85 [0.81, 0.86]	<0.001
	k_1 vs k_4	20.608 (0.232)	23.086 (0.442)	0.86 [0.82, 0.86]	<0.001
	k_2 vs k_3	21.177 (0.242)	21.448 (0.335)	0.53 [0.32, 0.69]	<0.001
	k_2 vs k_4	21.177 (0.242)	23.086 (0.442)	0.86 [0.83, 0.86]	<0.001
	k_3 vs k_4	21.448 (0.335)	23.086 (0.442)	0.86 [0.83, 0.86]	<0.001

Table S12. Post-hoc pairwise comparisons (Mann-Whitney U test) for the Mutual Information of the degree series (I) extracted from the duplex (x_1, y_1) configuration across varying simulated coupling strengths (k_1 to k_4). The table details the median and interquartile range (IQR) for each group, the effect size (r) with 95% confidence intervals, and the FDR-corrected p-values across different window lengths (N).

I - duplex (x1,y1)					
**Window (N)**	**Comparison**	**Median (IQR) Group 1**	**Median (IQR) Group 2**	**Effect Size r [95%CI]**	$p_{\mathbf{fdr}}$
30	k_1 vs k_2	0.216 (0.099)	0.169 (0.119)	0.18 [00.01, 0.43]	0.220
	k_1 vs k_3	0.216 (0.099)	0.220 (0.125)	0.03 [0.00. 0.31]	0.871
	k_1 vs k_4	0.216 (0.099)	0.210 (0.116)	0.03 [0.00, 0.31]	0.866
	k_2 vs k_3	0.169 (0.119)	0.220 (0.125)	0.13 [0.01, 0.40]	0.409
	k_2 vs k_4	0.169 (0.119)	0.210 (0.116)	0.15 [0.01, 0.41]	0.339
	k_3 vs k_4	0.220 (0.125)	0.210 (0.116)	0.00 [0.00, 0.29]	1.000
60	k_1 vs k_2	0.115 (0.047)	0.139 (0.064)	0.21 [0.02, 0.47]	0.152
	k_1 vs k_3	0.115 (0.047)	0.156 (0.086)	0.29 [0.04, 0.52]	0.038
	k_1 vs k_4	0.115 (0.047)	0.204 (0.067)	0.65 [0.47, 0.79]	<0.001
	k_2 vs k_3	0.139 (0.064)	0.156 (0.086)	0.11 [0.00, 0.35]	0.483
	k_2 vs k_4	0.139 (0.064)	0.204 (0.067)	0.49 [0.27, 0.68]	<0.001
	k_3 vs k_4	0.156 (0.086)	0.204 (0.067)	0.37 [0.13, 0.58]	0.007
120	k_1 vs k_2	0.073 (0.020)	0.085 (0.035)	0.25 [0.02, 0.49]	0.081
	k_1 vs k_3	0.073 (0.020)	0.090 (0.051)	0.33 [0.09, 0.55]	0.018
	k_1 vs k_4	0.073 (0.020)	0.136 (0.052)	0.67 [0.52, 0.78]	<0.001
	k_2 vs k_3	0.085 (0.035)	0.090 (0.051)	0.09 [0.00, 0.34]	0.059
	k_2 vs k_4	0.085 (0.035)	0.136 (0.052)	0.45 [0.23, 0.64]	<0.001
	k_3 vs k_4	0.090 (0.051)	0.136 (0.052)	0.36 [0.09, 0.56]	0.009
240	k_1 vs k_2	0.053 (0.013)	0.070 (0.020)	0.50 [0.27, 0.68]	<0.001
	k_1 vs k_3	0.053 (0.013)	0.078 (0.041)	0.69 [0.53, 0.81]	<0.001
	k_1 vs k_4	0.053 (0.013)	0.090 (0.016)	0.81 [0.71, 0.86]	<0.001
	k_2 vs k_3	0.070 (0.020)	0.078 (0.041)	0.31 [0.08, 0.53]	0.026
	k_2 vs k_4	0.070 (0.020)	0.090 (0.016)	0.58 [0.39, 0.74]	<0.001
	k_3 vs k_4	0.078 (0.041)	0.090 (0.016)	0.22 [0.02, 0.48]	0.128
480	k_1 vs k_2	0.038 (0.007)	0.044 (0.008)	0.43 [0.19, 0.63]	0.001
	k_1 vs k_3	0.038 (0.007)	0.053 (0.020)	0.69 [0.53, 0.80]	<0.001
	k_1 vs k_4	0.038 (0.007)	0.075 (0.015)	0.86 [0.83, 0.86]	<0.001
	k_2 vs k_3	0.044 (0.008)	0.053 (0.020)	0.46 [0.23, 0.66]	<0.001
	k_2 vs k_4	0.044 (0.008)	0.075 (0.015)	0.85 [0.81, 0.86]	<0.001
	k_3 vs k_4	0.053 (0.020)	0.075 (0.015)	0.62 [0.46, 0.76]	<0.001
600	k_1 vs k_2	0.035 (0.008)	0.039 (0.009)	0.39 [0.14, 0.59]	0.003
	k_1 vs k_3	0.035 (0.008)	0.050 (0.011)	0.72 [0.56, 0.81]	<0.001
	k_1 vs k_4	0.035 (0.008)	0.072 (0.012)	0.86 [0.83, 0.86]	<0.001
	k_2 vs k_3	0.039 (0.009)	0.050 (0.011)	0.49 [0.26, 0.68]	<0.001
	k_2 vs k_4	0.039 (0.009)	0.072 (0.012)	0.85 [0.82, 0.86]	<0.001
	k_3 vs k_4	0.050 (0.011)	0.072 (0.012)	0.81 [0.72, 0.86]	<0.001
720	k_1 vs k_2	0.034 (0.006)	0.039 (0.009)	0.47 [0.25, 0.65]	<0.001
	k_1 vs k_3	0.034 (0.006)	0.045 (0.011)	0.68 [0.51, 0.80]	<0.001
	k_1 vs k_4	0.034 (0.006)	0.072 (0.009)	0.86 [0.83, 0.86]	<0.001
	k_2 vs k_3	0.039 (0.009)	0.045 (0.011)	0.38 [0.15, 0.60]	0.006
	k_2 vs k_4	0.039 (0.009)	0.072 (0.009)	0.86 [0.83, 0.86]	<0.001
	k_3 vs k_4	0.045 (0.011)	0.072 (0.009)	0.85 [0.81, 0.86]	<0.001
900	k_1 vs k_2	0.031 (0.004)	0.035 (0.007)	0.37 [0.12, 0.59]	0.007
	k_1 vs k_3	0.031 (0.004)	0.039 (0.006)	0.71 [0.56, 0.81]	<0.001
	k_1 vs k_4	0.031 (0.004)	0.072 (0.013)	0.86 [0.82, 0.86]	<0.001
	k_2 vs k_3	0.035 (0.007)	0.039 (0.006)	0.42 [0.20, 0.62]	0.002
	k_2 vs k_4	0.035 (0.007)	0.072 (0.013)	0.86 [0.82, 0.86]	<0.001
	k_3 vs k_4	0.039 (0.006)	0.072 (0.013)	0.86 [0.83, 0.86]	<0.001
1200	k_1 vs k_2	0.025 (0.008)	0.032 (0.008)	0.50 [0.27, 0.67]	<0.001
	k_1 vs k_3	0.025 (0.008)	0.036 (0.006)	0.72 [0.59, 0.81]	<0.001
	k_1 vs k_4	0.025 (0.008)	0.075 (0.008)	0.86 [0.82, 0.86]	<0.001
	k_2 vs k_3	0.032 (0.008)	0.036 (0.006)	0.37 [0.14, 0.58]	0.006
	k_2 vs k_4	0.032 (0.008)	0.075 (0.008)	0.86 [0.82, 0.86]	<0.001
	k_3 vs k_4	0.036 (0.006)	0.075 (0.008)	0.86 [0.83, 0.86]	<0.001
2400	k_1 vs k_2	0.018 (0.004)	0.024 (0.004)	0.77 [0.66, 0.84]	<0.001
	k_1 vs k_3	0.018 (0.004)	0.031 (0.006)	0.86 [0.81, 0.86]	<0.001
	k_1 vs k_4	0.018 (0.004)	0.082 (0.007)	0.86 [0.82, 0.86]	<0.001
	k_2 vs k_3	0.024 (0.004)	0.031 (0.006)	0.74 [0.60, 0.83]	<0.001
	k_2 vs k_4	0.024 (0.004)	0.082 (0.007)	0.86 [0.82, 0.86]	<0.001
	k_3 vs k_4	0.031 (0.006)	0.082 (0.007)	0.86 [0.82, 0.86]	<0.001
3600	k_1 vs k_2	0.015 (0.003)	0.021 (0.003)	0.84 [0.79, 0.86]	<0.001
	k_1 vs k_3	0.015 (0.003)	0.027 (0.004)	0.86 [0.82, 0.86]	<0.001
	k_1 vs k_4	0.015 (0.003)	0.085 (0.005)	0.86 [0.83, 0.86]	<0.001
	k_2 vs k_3	0.021 (0.003)	0.027 (0.004)	0.82 [0.74, 0.85]	<0.001
	k_2 vs k_4	0.021 (0.003)	0.085 (0.005)	0.86 [0.83, 0.86]	<0.001
	k_3 vs k_4	0.027 (0.004)	0.085 (0.005)	0.86 [0.83, 0.86]	<0.001

Table S13. Post-hoc pairwise comparisons (Mann-Whitney U test) for the Mean Phase Coherence (MPC) of (x_1, y_1) across varying simulated coupling strengths (k_1 to k_4). The table details the median and interquartile range (IQR) for each group, the effect size (r) with 95% confidence intervals, and the FDR-corrected p-values across different window lengths (N).

**Duplex (x1,y1) - MPC**					
**Window (N)**	**Comparison**	**Median (IQR) Group 1**	**Median (IQR) Group 2**	**Effect Size** r $[95\%CI]$	$p_{\mathbf{fdr}}$
30	k_1 vs k_2	0.145 (0.072)	0.213 (0.124)	0.40 [0.18. 0.60]	0.002
	k_1 vs k_3	0.145 (0.072)	0.115 (0.086)	0.17 [0.01, 0.42]	0.216
	k_1 vs k_4	0.145 (0.072)	0.482 (0.437)	0.76 [0.63, 0.84]	<0.001
	k_2 vs k_3	0.213 (0.124)	0.115 (0.086)	0.50 [0.28, 0.67]	<0.001
	k_2 vs k_4	0.213 (0.124)	0.482 (0.437)	0.54 [0.34, 0.71]	<0.001
	k_3 vs k_4	0.115 (0.086)	0.482 (0.437)	0.78 [0.67, 0.85]	<0.001
60	k_1 vs k_2	0.064 (0.047)	0.049 (0.050)	0.11 [0.01, 0.37]	0.419
	k_1 vs k_3	0.064 (0.047)	0.068 (0.040)	0.08 [0.01, 0.32]	0.550
	k_1 vs k_4	0.064 (0.047)	0.561 (0.096)	0.86 [0.83, 0.86]	<0.001
	k_2 vs k_3	0.049 (0.050)	0.068 (0.040)	0.21 [0.02, 0.45]	0.112
	k_2 vs k_4	0.049 (0.050)	0.561 (0.096)	0.86 [0.82, 0.86]	<0.001
	k_3 vs k_4	0.068 (0.040)	0.561 (0.096)	0.86 [0.82, 0.86]	<0.001
120	k_1 vs k_2	0.039 (0.028)	0.038 (0.029)	0.04 [0.00, 0.30]	0.752
	k_1 vs k_3	0.039 (0.028)	0.049 (0.049)	0.15 [0.01, 0.40]	0.253
	k_1 vs k_4	0.039 (0.028)	0.574 (0.106)	0.86 [0.82, 0.86]	<0.001
	k_2 vs k_3	0.038 (0.029)	0.049 (0.049)	0.19 [0.01, 0.44]	0.165
	k_2 vs k_4	0.038 (0.029)	0.574 (0.106)	0.86 [0.82, 0.86]	<0.001
	k_3 vs k_4	0.049 (0.049)	0.574 (0.106)	0.86 [0.83, 0.86]	<0.001
240	k_1 vs k_2	0.022 (0.016)	0.027 (0.014)	0.17 [0.01, 0.41]	0.202
	k_1 vs k_3	0.022 (0.016)	0.047 (0.036)	0.56 [0.37, 0.72]	<0.001
	k_1 vs k_4	0.022 (0.016)	0.624 (0.045)	0.86 [0.82, 0.86]	<0.001
	k_2 vs k_3	0.027 (0.014)	0.047 (0.036)	0.50 [0.29, 0.69]	<0.001
	k_2 vs k_4	0.027 (0.014)	0.624 (0.045)	0.86 [0.82, 0.86]	<0.001
	k_3 vs k_4	0.047 (0.036)	0.624 (0.045)	0.86 [0.82, 0.86]	<0.001
480	k_1 vs k_2	0.018 (0.015)	0.028 (0.014)	0.36 [0.11, 0.58]	0.006
	k_1 vs k_3	0.018 (0.015)	0.044 (0.019)	0.76 [0.64, 0.83]	<0.001
	k_1 vs k_4	0.018 (0.015)	0.622 (0.050)	0.86 [0.82, 0.86]	<0.001
	k_2 vs k_3	0.028 (0.014)	0.044 (0.019)	0.61 [0.41, 0.76]	<0.001
	k_2 vs k_4	0.028 (0.014)	0.622 (0.050)	0.86 [0.82, 0.86]	<0.001
	k_3 vs k_4	0.044 (0.019)	0.622 (0.050)	0.86 [0.83, 0.86]	<0.001
600	k_1 vs k_2	0.016 (0.010)	0.020 (0.009)	0.32 [0.09, 0.55]	0.014
	k_1 vs k_3	0.016 (0.010)	0.043 (0.016)	0.81 [0.73, 0.85]	<0.001
	k_1 vs k_4	0.016 (0.010)	0.621 (0.042)	0.86 [0.82, 0.86]	<0.001
	k_2 vs k_3	0.020 (0.009)	0.043 (0.016)	0.75 [0.64, 0.83]	<0.001
	k_2 vs k_4	0.020 (0.009)	0.621 (0.042)	0.86 [0.82, 0.86]	<0.001
	k_3 vs k_4	0.043 (0.016)	0.621 (0.042)	0.86 [0.82, 0.86]	<0.001
720	k_1 vs k_2	0.014 (0.011)	0.019 (0.010)	0.28 [0.04. 0.52]	0.034
	k_1 vs k_3	0.014 (0.011)	0.042 (0.015)	0.81 [0.73, 0.85]	<0.001
	k_1 vs k_4	0.014 (0.011)	0.627 (0.035)	0.86 [0.82, 0.86]	<0.001
	k_2 vs k_3	0.019 (0.010)	0.042 (0.015)	0.79 [0.69, 0.84]	<0.001
	k_2 vs k_4	0.019 (0.010)	0.627 (0.035)	0.86 [0.83, 0.86]	<0.001
	k_3 vs k_4	0.042 (0.015)	0.627 (0.035)	0.86 [0.82, 0.86]	<0.001
900	k_1 vs k_2	0.013 (0.009)	0.019 (0.009)	0.41 [0.18, 0.60]	0.001
	k_1 vs k_3	0.013 (0.009)	0.044 (0.015)	0.85 [0.80, 0.86]	<0.001
	k_1 vs k_4	0.013 (0.009)	0.633 (0.030)	0.86 [0.82, 0.86]	<0.001
	k_2 vs k_3	0.019 (0.009)	0.044 (0.015)	0.82 [0.75, 0.85]	<0.001
	k_2 vs k_4	0.019 (0.009)	0.633 (0.030)	0.86 [0.82, 0.86]	<0.001
	k_3 vs k_4	0.044 (0.015)	0.633 (0.030)	0.86 [0.82, 0.86]	<0.001
1200	k_1 vs k_2	0.012 (0.008)	0.018 (0.007)	0.54 [0.35, 0.72]	<0.001
	k_1 vs k_3	0.012 (0.008)	0.041 (0.010)	0.85 [0.82, 0.86]	<0.001
	k_1 vs k_4	0.012 (0.008)	0.629 (0.021)	0.86 [0.82, 0.86]	<0.001
	k_2 vs k_3	0.018 (0.007)	0.041 (0.010)	0.84 [0.79, 0.86]	<0.001
	k_2 vs k_4	0.018 (0.007)	0.629 (0.021)	0.86 [0.82, 0.86]	<0.001
	k_3 vs k_4	0.041 (0.010)	0.629 (0.021)	0.86 [0.83, 0.86]	<0.001
2400	k_1 vs k_2	0.009 (0.006)	0.020 (0.005)	0.80 [0.69, 0.85]	<0.001
	k_1 vs k_3	0.009 (0.006)	0.042 (0.006)	0.86 [0.83, 0.86]	<0.001
	k_1 vs k_4	0.009 (0.006)	0.632 (0.014)	0.86 [0.83, 0.86]	<0.001
	k_2 vs k_3	0.020 (0.005)	0.042 (0.006)	0.86 [0.82, 0.86]	<0.001
	k_2 vs k_4	0.020 (0.005)	0.632 (0.014)	0.86 [0.82, 0.86]	<0.001
	k_3 vs k_4	0.042 (0.006)	0.632 (0.014)	0.86 [0.83, 0.86]	<0.001
3600	k_1 vs k_2	0.008 (0.004)	0.019 (0.003)	0.86 [0.82, 0.86]	<0.001
	k_1 vs k_3	0.008 (0.004)	0.040 (0.004)	0.86 [0.82, 0.86]	<0.001
	k_1 vs k_4	0.008 (0.004)	0.632 (0.011)	0.86 [0.82, 0.86]	<0.001
	k_2 vs k_3	0.019 (0.003)	0.040 (0.004)	0.86 [0.82, 0.86]	<0.001
	k_2 vs k_4	0.019 (0.003)	0.632 (0.011)	0.86 [0.82, 0.86]	<0.001
	k_3 vs k_4	0.040 (0.004)	0.632 (0.011)	0.86 [0.82, 0.86]	<0.001

Table S14. Post-hoc pairwise comparisons (Mann-Whitney U test) for the Average Degree (DA) extracted from the 6-layer multiplex configuration ($x_1, x_2, x_3, y_1, y_2, y_3$) across varying simulated coupling strengths (k_1 to k_4), with 30 realizations each. The table details the median and interquartile range (IQR) for each group, the effect size (r) with 95% confidence intervals, and the FDR-corrected p-values across different window lengths (N).

DA - multiplex (x1,x2,x3,y1,y2,y3)

Window (N)	Comparison	Median (IQR) Group 1	Median (IQR) Group 2	Effect Size r [95%CI]	$p_{\mathbf{fdr}}$
30	k_1 vs k_2	14.806 (4.042)	15.167 (4.097)	0.08 [0.00, 0.32]	0.6.08
	k_1 vs k_3	14.806 (4.042)	15.661 (2.939)	0.04 [0.00, 0.29]	0.808
	k_1 vs k_4	14.806 (4.042)	15.072 (5.239)	0.17 [0.01, 0.42]	0.227
	k_2 vs k_3	15.167 (4.097)	15.661 (2.939)	0.04 [0.00, 0.30]	0.795
	k_2 vs k_4	15.167 (4.097)	15.072 (5.239)	0.09 [0.01, 0.34]	0.583
	k_3 vs k_4	15.661 (2.939)	15.072 (5.239)	0.11 [0.01, 0.36]	0.460
60	k_1 vs k_2	19.783 (2.853)	21.414 (3.196)	0.29 [0.06, 0.50]	0.036
	k_1 vs k_3	19.783 (2.853)	21.244 (3.458)	0.19 [0.01, 0.42]	0.200
	k_1 vs k_4	19.783 (2.853)	22.503 (5.149)	0.34 [0.10, 0.57]	0.012
	k_2 vs k_3	21.414 (3.196)	21.244 (3.458)	0.08 [0.00, 0.36]	0.594
	k_2 vs k_4	21.414 (3.196)	22.503 (5.149)	0.15 [0.01, 0.40]	0.318
	k_3 vs k_4	21.244 (3.458)	22.503 (5.149)	0.22 [0.02, 0.46]	0.128
120	k_1 vs k_2	24.443 (1.922)	25.528 (1.414)	0.38 [0.16, 0.60]	0.004
	k_1 vs k_3	24.443 (1.922)	25.208 (1.665)	0.35 [0.12, 0.55]	0.009
	k_1 vs k_4	24.443 (1.922)	26.540 (2.092)	0.61 [0.42, 0.76]	<0.001
	k_2 vs k_3	25.528 (1.414)	25.208 (1.665)	0.02 [0.00, 0.30]	0.883
	k_2 vs k_4	25.528 (1.414)	26.540 (2.092)	0.48 [0.23, 0.67]	<0.001
	k_3 vs k_4	25.208 (1.665)	26.540 (2.092)	0.46 [0.22, 0.66]	<0.001
240	k_1 vs k_2	26.156 (0.758)	26.610 (0.889)	0.41 [0.16, 0.62]	0.002
	k_1 vs k_3	26.156 (0.758)	27.121 (0.459)	0.69 [0.53, 0.81]	<0.001
	k_1 vs k_4	26.156 (0.758)	29.333 (1.028)	0.86 [0.82, 0.86]	<0.001
	k_2 vs k_3	26.610 (0.889)	27.121 (0.459)	0.35 [0.11, 0.56]	0.0107
	k_2 vs k_4	26.610 (0.889)	29.333 (1.028)	0.86 [0.83, 0.86]	<0.001
	k_3 vs k_4	27.121 (0.459)	29.333 (1.028)	0.86 [0.82, 0.86]	<0.001
480	k_1 vs k_2	26.977 (0.379)	27.589 (0.298)	0.70 [0.57, 0.81]	<0.001
	k_1 vs k_3	26.977 (0.379)	28.012 (0.291)	0.82 [0.74, 0.86]	<0.001
	k_1 vs k_4	26.977 (0.379)	30.423 (0.655)	0.86 [0.82, 0.86]	<0.001
	k_2 vs k_3	27.589 (0.298)	28.012 (0.291)	0.57 [0.36, 0.74]	<0.001
	k_2 vs k_4	27.589 (0.298)	30.423 (0.655)	0.86 [0.83, 0.86]	<0.001
	k_3 vs k_4	28.012 (0.291)	30.423 (0.655)	0.86 [0.82, 0.86]	<0.001
600	k_1 vs k_2	27.152 (0.443)	27.693 (0.334)	0.67 [0.51, 0.79]	<0.001
	k_1 vs k_3	27.152 (0.443)	28.141 (0.384)	0.85 [0.82, 0.86]	<0.001
	k_1 vs k_4	27.152 (0.443)	30.614 (0.588)	0.86 [0.82, 0.86]	<0.001
	k_2 vs k_3	27.693 (0.334)	28.141 (0.384)	0.75 [0.64, 0.83]	<0.001
	k_2 vs k_4	27.693 (0.334)	30.614 (0.588)	0.86 [0.83, 0.86]	<0.001
	k_3 vs k_4	28.141 (0.384)	30.614 (0.588)	0.86 [0.82, 0.86]	<0.001
720	k_1 vs k_2	27.375 (0.386)	27.855 (0.214)	0.68 [0.51, 0.80]	<0.001
	k_1 vs k_3	27.375 (0.386)	28.442 (0.351)	0.86 [0.82, 0.86]	<0.001
	k_1 vs k_4	27.375 (0.386)	30.863 (0.627)	0.86 [0.83, 0.86]	<0.001
	k_2 vs k_3	27.855 (0.214)	28.442 (0.351)	0.72 [0.57, 0.82]	<0.001
	k_2 vs k_4	27.855 (0.214)	30.863 (0.627)	0.86 [0.82, 0.86]	<0.001
	k_3 vs k_4	28.442 (0.351)	30.863 (0.627)	0.86 [0.82, 0.86]	<0.001
900	k_1 vs k_2	27.505 (0.458)	28.084 (0.324)	0.79 [0.69, 0.84]	<0.001
	k_1 vs k_3	27.505 (0.458)	28.553 (0.195)	0.86 [0.82, 0.86]	<0.001
	k_1 vs k_4	27.505 (0.458)	30.999 (0.464)	0.86 [0.82, 0.86]	<0.001
	k_2 vs k_3	28.084 (0.324)	28.553 (0.195)	0.66 [0.49, 0.79]	<0.001
	k_2 vs k_4	28.084 (0.324)	30.999 (0.464)	0.86 [0.83, 0.86]	<0.001
	k_3 vs k_4	28.553 (0.195)	30.999 (0.464)	0.86 [0.82, 0.86]	<0.001
1200	k_1 vs k_2	27.634 (0.311)	28.237 (0.290)	0.83 [0.77, 0.86]	<0.001
	k_1 vs k_3	27.634 (0.311)	28.783 (0.281)	0.86 [0.83, 0.86]	<0.001
	k_1 vs k_4	27.634 (0.311)	31.245 (0.374)	0.86 [0.82, 0.86]	<0.001
	k_2 vs k_3	28.237 (0.290)	28.783 (0.281)	0.74 [0.61, 0.83]	<0.001
	k_2 vs k_4	28.237 (0.290)	31.245 (0.374)	0.86 [0.83, 0.86]	<0.001
	k_3 vs k_4	28.783 (0.281)	31.245 (0.374)	0.86 [0.82, 0.86]	<0.001
2400	k_1 vs k_2	27.870 (0.264)	28.475 (0.204)	0.85 [0.79, 0.86]	<0.001
	k_1 vs k_3	27.870 (0.264)	28.979 (0.202)	0.86 [0.82, 0.86]	<0.001
	k_1 vs k_4	27.870 (0.264)	31.467 (0.306)	0.86 [0.83, 0.86]	<0.001
	k_2 vs k_3	28.475 (0.204)	28.979 (0.202)	0.83 [0.77, 0.86]	<0.001
	k_2 vs k_4	28.475 (0.204)	31.467 (0.306)	0.86 [0.83, 0.86]	<0.001
	k_3 vs k_4	28.979 (0.202)	31.467 (0.306)	0.86 [0.83, 0.86]	<0.001
3600	k_1 vs k_2	27.941 (0.125)	28.606 (0.153)	0.86 [0.82, 0.86]	<0.001
	k_1 vs k_3	27.941 (0.125)	29.056 (0.132)	0.86 [0.82, 0.86]	<0.001
	k_1 vs k_4	27.941 (0.125)	31.526 (0.269)	0.86 [0.83, 0.86]	<0.001
	k_2 vs k_3	28.606 (0.153)	29.056 (0.132)	0.86 [0.82, 0.86]	<0.001
	k_2 vs k_4	28.606 (0.153)	31.526 (0.269)	0.86 [0.82, 0.86]	<0.001
	k_3 vs k_4	29.056 (0.132)	31.526 (0.269)	0.86 [0.82, 0.86]	<0.001

Table S15. Post-hoc pairwise comparisons (Mann-Whitney U test) for the Mutual Information of the degree series ($\bar{I}$) extracted from the 6-layer multiplex configuration ($x_1, x_2, x_3, y_1, y_2, y_3$) across varying simulated coupling strengths (k_1 to k_4). The table details the median and interquartile range (IQR) for each group, the effect size (r) with 95% confidence intervals, and the FDR-corrected p-values across different window lengths (N).

$\bar{I}$ - multiplex (x1,x2,x3,y1,y2,y3)					
**Window (N)**	**Comparison**	**Median (IQR) Group 1**	**Median (IQR) Group 2**	**Effect Size** r [95%CI]	$p_{\mathbf{fdr}}$
30	k_1 vs k_2	0.254 (0.048)	0.249 (0.051)	0.05 [0.00, 0.31]	0.754
	k_1 vs k_3	0.254 (0.048)	0.275 (0.058)	0.16 [0.01, 0.41]	0.268
	k_1 vs k_4	0.254 (0.048)	0.284 (0.070)	0.20 [0.02, 0.46]	0.1524
	k_2 vs k_3	0.249 (0.051)	0.275 (0.058)	0.20 [0.01, 0.45]	0.155
	k_2 vs k_4	0.249 (0.051)	0.284 (0.070)	0.24 [0.03, 0.49]	0.084
	k_3 vs k_4	0.275 (0.058)	0.284 (0.070)	0.08 [0.00, 0.34]	0.608
60	k_1 vs k_2	0.189 (0.031)	0.185 (0.023)	0.07 [0.00, 0.35]	0.631
	k_1 vs k_3	0.189 (0.031)	0.197 (0.039)	0.19 [0.01, 0.42]	0.201
	k_1 vs k_4	0.189 (0.031)	0.277 (0.074)	0.82 [0.75, 0.86]	<0.001
	k_2 vs k_3	0.185 (0.023)	0.197 (0.039)	0.23 [0.02, 0.46]	0.111
	k_2 vs k_4	0.185 (0.023)	0.277 (0.074)	0.80 [0.71, 0.85]	<0.001
	k_3 vs k_4	0.197 (0.039)	0.277 (0.074)	0.73 [0.60, 0.82]	<0.001
120	k_1 vs k_2	0.149 (0.010)	0.157 (0.014)	0.23 [0.02, 0.46]	0.109
	k_1 vs k_3	0.149 (0.010)	0.146 (0.023)	0.11 [0.00, 0.36]	0.461
	k_1 vs k_4	0.149 (0.010)	0.220 (0.038)	0.82 [0.73, 0.86]	<0.001
	k_2 vs k_3	0.157 (0.014)	0.146 (0.023)	0.25 [0.03, 0.49]	0.0705
	k_2 vs k_4	0.157 (0.014)	0.220 (0.038)	0.80 [0.69, 0.86]	<0.001
	k_3 vs k_4	0.146 (0.023)	0.220 (0.038)	0.83 [0.75, 0.86]	<0.001
240	k_1 vs k_2	0.130 (0.008)	0.119 (0.010)	0.60 [0.42, 0.75]	<0.001
	k_1 vs k_3	0.130 (0.008)	0.118 (0.014)	0.57 [0.35, 0.73]	<0.001
	k_1 vs k_4	0.130 (0.008)	0.180 (0.012)	0.86 [0.82, 0.86]	5.648E-17
	k_2 vs k_3	0.119 (0.010)	0.118 (0.014)	0.03 [0.01, 0.32]	0.856
	k_2 vs k_4	0.119 (0.010)	0.180 (0.012)	0.86 [0.82, 0.86]	<0.001
	k_3 vs k_4	0.118 (0.014)	0.180 (0.012)	0.86 [0.83, 0.86]	<0.001
480	k_1 vs k_2	0.118 (0.007)	0.099 (0.010)	0.82 [0.74, 0.85]	<0.001
	k_1 vs k_3	0.118 (0.007)	0.093 (0.010)	0.81 [0.68, 0.86]	<0.001
	k_1 vs k_4	0.118 (0.007)	0.159 (0.010)	0.86 [0.82, 0.86]	<0.001
	k_2 vs k_3	0.099 (0.010)	0.093 (0.010)	0.30 [0.05, 0.51]	0.032
	k_2 vs k_4	0.099 (0.010)	0.159 (0.010)	0.86 [0.82, 0.86]	<0.001
	k_3 vs k_4	0.093 (0.010)	0.159 (0.010)	0.86 [0.82, 0.86]	<0.001
600	k_1 vs k_2	0.115 (0.007)	0.098 (0.012)	0.83 [0.78, 0.86]	<0.001
	k_1 vs k_3	0.115 (0.007)	0.087 (0.010)	0.86 [0.82, 0.86]	<0.001
	k_1 vs k_4	0.115 (0.007)	0.157 (0.011)	0.86 [0.82, 0.86]	<0.001
	k_2 vs k_3	0.098 (0.012)	0.087 (0.010)	0.62 [0.44, 0.76]	<0.001
	k_2 vs k_4	0.098 (0.012)	0.157 (0.011)	0.86 [0.82, 0.86]	<0.001
	k_3 vs k_4	0.087 (0.010)	0.157 (0.011)	0.86 [0.82, 0.86]	<0.001
720	k_1 vs k_2	0.112 (0.006)	0.096 (0.009)	0.85 [0.81, 0.86]	<0.001
	k_1 vs k_3	0.112 (0.006)	0.085 (0.007)	0.86 [0.82, 0.86]	<0.001
	k_1 vs k_4	0.112 (0.006)	0.157 (0.011)	0.86 [0.83, 0.86]	<0.001
	k_2 vs k_3	0.096 (0.009)	0.085 (0.007)	0.73 [0.60, 0.81]	<0.001
	k_2 vs k_4	0.096 (0.009)	0.157 (0.011)	0.86 [0.83, 0.86]	<0.001
	k_3 vs k_4	0.085 (0.007)	0.157 (0.011)	0.86 [0.81, 0.86]	<0.001
900	k_1 vs k_2	0.110 (0.005)	0.089 (0.005)	0.86 [0.81, 0.86]	<0.001
	k_1 vs k_3	0.110 (0.005)	0.080 (0.007)	0.86 [0.82, 0.86]	<0.001
	k_1 vs k_4	0.110 (0.005)	0.155 (0.012)	0.86 [0.83, 0.86]	<0.001
	k_2 vs k_3	0.089 (0.005)	0.080 (0.007)	0.71 [0.57, 0.82]	<0.001
	k_2 vs k_4	0.089 (0.005)	0.155 (0.012)	0.86 [0.82, 0.86]	<0.001
	k_3 vs k_4	0.080 (0.007)	0.155 (0.012)	0.86 [0.82, 0.86]	<0.001
1200	k_1 vs k_2	0.110 (0.004)	0.088 (0.004)	0.86 [0.83, 0.86]	<0.001
	k_1 vs k_3	0.110 (0.004)	0.077 (0.008)	0.86 [0.82, 0.86]	<0.001
	k_1 vs k_4	0.110 (0.004)	0.155 (0.011)	0.86 [0.83, 0.86]	<0.001
	k_2 vs k_3	0.088 (0.004)	0.077 (0.008)	0.78 [0.68, 0.84]	<0.001
	k_2 vs k_4	0.088 (0.004)	0.155 (0.011)	0.86 [0.82, 0.86]	<0.001
	k_3 vs k_4	0.077 (0.008)	0.155 (0.011)	0.86 [0.82, 0.86]	<0.001
2400	k_1 vs k_2	0.108 (0.004)	0.083 (0.004)	0.86 [0.82, 0.86]	<0.001
	k_1 vs k_3	0.108 (0.004)	0.073 (0.004)	0.86 [0.83, 0.86]	<0.001
	k_1 vs k_4	0.108 (0.004)	0.155 (0.007)	0.86 [0.82, 0.86]	<0.001
	k_2 vs k_3	0.083 (0.004)	0.073 (0.004)	0.78 [0.67, 0.84]	<0.001
	k_2 vs k_4	0.083 (0.004)	0.155 (0.007)	0.86 [0.82, 0.86]	<0.001
	k_3 vs k_4	0.073 (0.004)	0.155 (0.007)	0.86 [0.82, 0.86]	<0.001
3600	k_1 vs k_2	0.109 (0.003)	0.080 (0.004)	0.86 [0.82, 0.86]	<0.001
	k_1 vs k_3	0.109 (0.003)	0.072 (0.003)	0.86 [0.83, 0.86]	<0.001
	k_1 vs k_4	0.109 (0.003)	0.157 (0.006)	0.86 [0.82, 0.86]	<0.001
	k_2 vs k_3	0.080 (0.004)	0.072 (0.003)	0.85 [0.81, 0.86]	<0.001
	k_2 vs k_4	0.080 (0.004)	0.157 (0.006)	0.86 [0.82, 0.86]	<0.001
	k_3 vs k_4	0.072 (0.003)	0.157 (0.006)	0.86 [0.82, 0.86]	<0.001

Table S16. Post-hoc pairwise comparisons (Mann-Whitney U test) for the Mean Phase Coherence (MPC) averaged for all combinations of $(x_1, x_2, x_3, y_1, y_2, y_3)$ across varying simulated coupling strengths (k_1 to k_4), with 30 realizations each. The table details the median and interquartile range (IQR) for each group, the effect size (r) with 95% confidence intervals, and the FDR-corrected p-values across different window lengths (N).

Multiplex (x1,x2,x3,y1,y2,y3) - MPC					
**Window (N)**	**Comparison**	**Median (IQR) Group 1**	**Median (IQR) Group 2**	**Effect Size r [95%CI]**	$p_{\mathbf{fdr}}$
30	k_1 vs k_2	0.331 (0.029)	0.349 (0.056)	0.29 [0.06, 0.50]	0.027
	k_1 vs k_3	0.331 (0.029)	0.345 (0.048)	0.25 [0.03, 0.49]	0.057
	k_1 vs k_4	0.331 (0.029)	0.504 (0.189)	0.83 [0.76, 0.86]	<0.001
	k_2 vs k_3	0.349 (0.056)	0.345 (0.048)	0.02 [0.00, 0.28]	0.866
	k_2 vs k_4	0.349 (0.056)	0.504 (0.189)	0.75 [0.62, 0.83]	<0.001
	k_3 vs k_4	0.345 (0.048)	0.504 (0.189)	0.75 [0.62, 0.83]	<0.001
60	k_1 vs k_2	0.290 (0.017)	0.294 (0.014)	0.21 [0.02, 0.46]	0.104
	k_1 vs k_3	0.290 (0.017)	0.329 (0.022)	0.73 [0.57, 0.84]	<0.001
	k_1 vs k_4	0.290 (0.017)	0.636 (0.051)	0.86 [0.82, 0.86]	<0.001
	k_2 vs k_3	0.294 (0.014)	0.329 (0.022)	0.63 [0.43, 0.77]	<0.001
	k_2 vs k_4	0.294 (0.014)	0.636 (0.051)	0.86 [0.83, 0.86]	<0.001
	k_3 vs k_4	0.329 (0.022)	0.636 (0.051)	0.86 [0.83, 0.86]	<0.001
120	k_1 vs k_2	0.268 (0.025)	0.278 (0.012)	0.34 [0.12, 0.56]	0.007
	k_1 vs k_3	0.268 (0.025)	0.309 (0.019)	0.78 [0.67, 0.84]	<0.001
	k_1 vs k_4	0.268 (0.025)	0.638 (0.048)	0.86 [0.82, 0.86]	<0.001
	k_2 vs k_3	0.278 (0.012)	0.309 (0.019)	0.72 [0.56, 0.82]	<0.001
	k_2 vs k_4	0.278 (0.012)	0.638 (0.048)	0.86 [0.83, 0.86]	<0.001
	k_3 vs k_4	0.309 (0.019)	0.638 (0.048)	0.86 [0.83, 0.86]	<0.001
240	k_1 vs k_2	0.251 (0.015)	0.264 (0.008)	0.61 [0.42, 0.74]	<0.001
	k_1 vs k_3	0.251 (0.015)	0.299 (0.027)	0.86 [0.82, 0.86]	<0.001
	k_1 vs k_4	0.251 (0.015)	0.632 (0.031)	0.86 [0.82, 0.86]	<0.001
	k_2 vs k_3	0.264 (0.008)	0.299 (0.027)	0.85 [0.79, 0.86]	<0.001
	k_2 vs k_4	0.264 (0.008)	0.632 (0.031)	0.86 [0.82, 0.86]	<0.001
	k_3 vs k_4	0.299 (0.027)	0.632 (0.031)	0.86 [0.82, 0.86]	<0.001
480	k_1 vs k_2	0.248 (0.006)	0.260 (0.009)	0.70 [0.54, 0.82]	<0.001
	k_1 vs k_3	0.248 (0.006)	0.296 (0.017)	0.86 [0.83, 0.86]	<0.001
	k_1 vs k_4	0.248 (0.006)	0.625 (0.024)	0.86 [0.82, 0.86]	<0.001
	k_2 vs k_3	0.260 (0.009)	0.296 (0.017)	0.86 [0.82, 0.86]	<0.001
	k_2 vs k_4	0.260 (0.009)	0.625 (0.024)	0.86 [0.82, 0.86]	<0.001
	k_3 vs k_4	0.296 (0.017)	0.625 (0.024)	0.86 [0.82, 0.86]	<0.001
600	k_1 vs k_2	0.247 (0.007)	0.259 (0.009)	0.75 [0.62, 0.83]	<0.001
	k_1 vs k_3	0.247 (0.007)	0.300 (0.018)	0.86 [0.82, 0.86]	<0.001
	k_1 vs k_4	0.247 (0.007)	0.624 (0.030)	0.86 [0.82, 0.86]	<0.001
	k_2 vs k_3	0.259 (0.009)	0.300 (0.018)	0.86 [0.82, 0.86]	<0.001
	k_2 vs k_4	0.259 (0.009)	0.624 (0.030)	0.86 [0.83, 0.86]	<0.001
	k_3 vs k_4	0.300 (0.018)	0.624 (0.030)	0.86 [0.82, 0.86]	<0.001
720	k_1 vs k_2	0.246 (0.005)	0.260 (0.007)	0.81 [0.73, 0.85]	<0.001
	k_1 vs k_3	0.246 (0.005)	0.298 (0.016)	0.86 [0.82, 0.86]	<0.001
	k_1 vs k_4	0.246 (0.005)	0.627 (0.025)	0.86 [0.82, 0.86]	<0.001
	k_2 vs k_3	0.260 (0.007)	0.298 (0.016)	0.86 [0.82, 0.86]	<0.001
	k_2 vs k_4	0.260 (0.007)	0.627 (0.025)	0.86 [0.83, 0.86]	<0.001
	k_3 vs k_4	0.298 (0.016)	0.627 (0.025)	0.86 [0.83, 0.86]	<0.001
900	k_1 vs k_2	0.246 (0.007)	0.260 (0.005)	0.86 [0.82, 0.86]	<0.001
	k_1 vs k_3	0.246 (0.007)	0.298 (0.014)	0.86 [0.82, 0.86]	<0.001
	k_1 vs k_4	0.246 (0.007)	0.631 (0.020)	0.86 [0.82, 0.86]	<0.001
	k_2 vs k_3	0.260 (0.005)	0.298 (0.014)	0.86 [0.83, 0.86]	<0.001
	k_2 vs k_4	0.260 (0.005)	0.631 (0.020)	0.86 [0.82, 0.86]	<0.001
	k_3 vs k_4	0.298 (0.014)	0.631 (0.020)	0.86 [0.82, 0.86]	<0.001
1200	k_1 vs k_2	0.244 (0.007)	0.259 (0.006)	0.86 [0.83, 0.86]	<0.001
	k_1 vs k_3	0.244 (0.007)	0.300 (0.014)	0.86 [0.82, 0.86]	<0.001
	k_1 vs k_4	0.244 (0.007)	0.631 (0.010)	0.86 [0.82, 0.86]	<0.001
	k_2 vs k_3	0.259 (0.006)	0.300 (0.014)	0.86 [0.82, 0.86]	<0.001
	k_2 vs k_4	0.259 (0.006)	0.631 (0.010)	0.86 [0.83, 0.86]	<0.001
	k_3 vs k_4	0.300 (0.014)	0.631 (0.010)	0.86 [0.83, 0.86]	<0.001
2400	k_1 vs k_2	0.243 (0.004)	0.259 (0.003)	0.86 [0.82, 0.86]	<0.001
	k_1 vs k_3	0.243 (0.004)	0.299 (0.006)	0.86 [0.82, 0.86]	<0.001
	k_1 vs k_4	0.243 (0.004)	0.634 (0.012)	0.86 [0.82, 0.86]	<0.001
	k_2 vs k_3	0.259 (0.003)	0.299 (0.006)	0.86 [0.82, 0.86]	<0.001
	k_2 vs k_4	0.259 (0.003)	0.634 (0.012)	0.86 [0.83, 0.86]	<0.001
	k_3 vs k_4	0.299 (0.006)	0.634 (0.012)	0.86 [0.83, 0.86]	<0.001
3600	k_1 vs k_2	0.242 (0.002)	0.258 (0.003)	0.86 [0.83, 0.86]	<0.001
	k_1 vs k_3	0.242 (0.002)	0.299 (0.004)	0.86 [0.83, 0.86]	<0.001
	k_1 vs k_4	0.242 (0.002)	0.635 (0.006)	0.86 [0.83, 0.86]	<0.001
	k_2 vs k_3	0.258 (0.003)	0.299 (0.004)	0.86 [0.82, 0.86]	<0.001
	k_2 vs k_4	0.258 (0.003)	0.635 (0.006)	0.86 [0.82, 0.86]	<0.001
	k_3 vs k_4	0.299 (0.004)	0.635 (0.006)	0.86 [0.82, 0.86]	<0.001

S1.3. Correlation with MPC

Table S17. Spearman rank correlation analysis between Mean Phase Coherence (MPC) and network measues—Degree (DA) and Mutual Information (I)—for the bivariate series $(x1, y1)$. Results are reported as a function of the window size (N) across $n = 120$ observations. The table shows the Spearman correlation coefficient (r), uncorrected p-values, and FDR-corrected p-values (p_{FDR}).

(x1,y1)						
**N**	**var1**	**var2**	**n°obs**	r	**p–value**	p_{FDR}
30	MPC	DA	120	0.320	<0.001	<0.01
60	MPC	DA	120	-0.082	0.372	0.390
120	MPC	DA	120	0.127	0.168	0.180
240	MPC	DA	120	0.598	<0.001	<0.001
480	MPC	DA	120	0.708	<0.001	<0.001
600	MPC	DA	120	0.784	<0.001	<0.001
720	MPC	DA	120	0.744	<0.001	<0.001
900	MPC	DA	120	0.754	<0.001	<0.001
1200	MPC	DA	120	0.826	<0.001	<0.001
2400	MPC	DA	120	0.864	<0.001	<0.001
3600	MPC	DA	120	0.904	<0.001	<0.001
30	MPC	I	120	0.071	0.437	0.447
60	MPC	I	120	0.334	<0.001	<0.001
120	MPC	I	120	0.332	<0.001	<0.001
240	MPC	I	120	0.555	<0.001	<0.001
480	MPC	I	120	0.726	<0.001	<0.001
600	MPC	I	120	0.769	<0.001	<0.001
720	MPC	I	120	0.776	<0.001	<0.001
900	MPC	I	120	0.826	<0.001	<0.001
1200	MPC	I	120	0.815	<0.001	<0.001
2400	MPC	I	120	0.915	<0.001	<0.001
3600	MPC	I	120	0.929	<0.001	<0.001

S2. Multisystem Cardiovascular Sensing (HYPOL Dataset)

This section includes the detailed results from the single-layer, duplex, and multiplex configurations applied to the HYPOL dataset.

Table S18. Statistical results of the Mann-Whitney U test for the Degree (DA) of the HYPOL dataset, comparing sex-based cardiovascular differences (147 women and 129 men) on systolic, diastolic and beat-to-beat separately. The table reports the median and interquartile range (IQR) for each group, the rank correlation effect size (r) with 95% confidence intervals, and FDR-corrected p-values as a function of the window size (N).

		DA			
**Series**	**Window size (N)**	**Median (IQR) Women**	**Median (IQR) Men**	**Effect Size r [95% CI]**	p_{fdr}
RR	60	7.095 (2.042)	7.079 (1.783)	0.02 [0.00, 0.13]	0.699
	120	7.489 (1.797)	7.517 (1.593)	0.03 [0.00, 0.15]	0.699
	180	7.611 (1.652)	7.525 (1.537)	0.02 [0.00, 0.14]	0.699
	240	7.530 (1.479)	7.671 (1.421)	0.06 [0.00, 0.17]	0.376
	300	7.562 (1.335)	7.655 (1.249)	0.09 [0.01, 0.21]	0.166
	360	7.434 (1.406)	7.651 (1.322)	0.11 [0.01, 0.24]	0.089
	420	7.460 (1.396)	7.781 (1.414)	0.13 [0.01, 0.24]	0.089
	480	7.440 (1.361)	7.772 (1.304)	0.11 [0.01, 0.24]	0.089
	540	7.481 (1.335)	7.754 (1.262)	0.13 [0.01, 0.23]	0.089
	600	7.477 (1.329)	7.796 (1.189)	0.12 [0.01, 0.24]	0.089
	660	7.473 (1.254)	7.764 (1.223)	0.12 [0.01, 0.23]	0.089
	720	7.531 (1.156)	7.790 (1.156)	0.12 [0.02, 0.24]	0.089
	780	7.492 (1.155)	7.793 (1.172)	0.12 [0.01, 0.24]	0.089
	840	7.493 (1.142)	7.758 (1.155)	0.12 [0.01, 0.23]	0.089
	900	7.476 (1.084)	7.755 (1.107)	0.12 [0.02, 0.24]	0.089
DBP	60	8.247 (1.963)	8.349 (2.364)	0.04 [0.00, 0.16]	0.679
	120	8.605 (1.746)	8.692 (1.748)	0.02 [0.00, 0.14]	0.838
	180	8.749 (1.744)	8.917 (1.648)	0.01 [0.00, 0.13]	0.933
	240	8.858 (1.459)	9.004 (1.776)	0.02 [0.00, 0.14]	0.838
	300	8.944 (1.380)	9.101 (1.830)	0.05 [0.00, 0.17]	0.570
	360	8.897 (1.410)	9.003 (1.767)	0.05 [0.00, 0.16]	0.570
	420	8.904 (1.411)	9.063 (1.605)	0.06 [0.00, 0.16]	0.570
	480	8.973 (1.317)	9.061 (1.744)	0.08 [0.00, 0.21]	0.384
	540	8.967 (1.327)	9.177 (1.659)	0.08 [0.01, 0.19]	0.384
	600	9.006 (1.368)	9.182 (1.696)	0.07 [0.00, 0.18]	0.474
	660	9.006 (1.316)	9.170 (1.561)	0.08 [0.00, 0.20]	0.384
	720	9.004 (1.323)	9.114 (1.671)	0.08 [0.01, 0.19]	0.384
	780	9.026 (1.285)	9.097 (1.582)	0.09 [0.01, 0.20]	0.384
	840	8.979 (1.171)	9.067 (1.535)	0.09 [0.00, 0.21]	0.384
	900	9.051 (1.215)	9.139 (1.522)	0.09 [0.01, 0.20]	0.384
SBP	60	8.741 (2.971)	8.592 (2.925)	0.03 [0.00, 0.15]	0.667
	120	9.353 (2.618)	9.697 (2.546)	0.08 [0.00, 0.20]	0.180
	180	9.732 (2.651)	10.121 (2.568)	0.11 [0.01, 0.22]	0.093
	240	9.918 (2.350)	10.418 (2.180)	0.14 [0.03, 0.26]	0.021
	300	9.877 (2.085)	10.665 (2.080)	0.19 [0.08, 0.31]	0.002
	360	9.961 (2.208)	10.740 (1.971)	0.20 [0.09, 0.32]	0.001
	420	9.943 (2.275)	10.851 (1.999)	0.22 [0.11, 0.34]	<0.001
	480	9.969 (2.248)	10.845 (1.940)	0.25 [0.13, 0.36]	<0.001
	540	9.981 (2.300)	10.847 (2.111)	0.24 [0.13, 0.35]	<0.001
	600	10.027 (2.315)	10.811 (2.127)	0.24 [0.13, 0.35]	<0.001
	660	10.047 (2.358)	11.037 (2.335)	0.25 [0.14, 0.37]	<0.001
	720	10.062 (2.295)	11.014 (2.103)	0.26 [0.15, 0.38]	<0.001
	780	10.073 (2.189)	11.048 (2.402)	0.27 [0.15, 0.38]	<0.001
	840	10.132 (2.316)	11.010 (2.315)	0.27 [0.15, 0.38]	<0.001
	900	10.153 (2.198)	11.054 (2.113)	0.28 [0.17, 0.39]	<0.001

Table S19. Statistical results of the Mann-Whitney U test for the Degree (DA) and Mutual Information (I) of the HYPOL dataset, comparing sex-based cardiovascular differences (147 women and 129 men) using beat-to-beat series and systolic. The table reports the median and interquartile range (IQR) for each group, the rank correlation effect size (r) with 95% confidence intervals, and FDR-corrected p-values as a function of the window size (N).

			(RR-SBP)		
**Measure**	**Window size (N)**	**Median (IQR) Women**	**Median (IQR) Men**	**Effect Size r [95% CI]**	$p_{\mathbf{fdr}}$
DA	60	8.790 (1.869)	8.920 (2.219)	0.02 [0.00, 0.15]	0.760
	120	9.400 (2.066)	9.699 (1.658)	0.07 [0.00, 0.19]	0.353
	180	9.651 (1.860)	9.975 (1.491)	0.10 [0.01, 0.22]	0.204
	240	9.724 (1.456)	10.082 (1.279)	0.15 [0.03, 0.26]	0.047
	300	9.732 (1.526)	10.135 (1.395)	0.20 [0.07, 0.31]	0.005
	360	9.814 (1.375)	10.260 (1.304)	0.22 [0.10, 0.32]	0.002
	420	9.753 (1.522)	10.417 (1.283)	0.24 [0.13, 0.35]	<0.001
	480	9.846 (1.436)	10.427 (1.261)	0.25 [0.13, 0.35]	<0.001
	540	9.834 (1.351)	10.480 (1.184)	0.26 [0.13, 0.36]	<0.001
	600	9.854 (1.341)	10.473 (1.227)	0.25 [0.14, 0.37]	<0.001
	660	9.896 (1.277)	10.481 (1.262)	0.26 [0.15, 0.37]	<0.001
	720	9.850 (1.270)	10.503 (1.319)	0.27 [0.16, 0.40]	<0.001
	780	9.869 (1.244)	10.514 (1.239)	0.28 [0.17, 0.39]	<0.001
	840	9.893 (1.257)	10.510 (1.238)	0.28 [0.17, 0.39]	<0.001
	900	9.906 (1.255)	10.510 (1.186)	0.29 [0.18, 0.40]	<0.001
I	60	0.115 (0.076)	0.112 (0.069)	0.02 [0.00, 0.15]	0.777
	120	0.089 (0.045)	0.089 (0.047)	0.03 [0.00, 0.15]	0.682
	180	0.076 (0.035)	0.079 (0.035)	0.12 [0.01, 0.22]	0.122
	240	0.067 (0.027)	0.072 (0.030)	0.16 [0.04, 0.28]	0.028
	300	0.063 (0.024)	0.066 (0.028)	0.11 [0.01, 0.22]	0.154
	360	0.058 (0.023)	0.062 (0.026)	0.14 [0.03, 0.26]	0.049
	420	0.054 (0.025)	0.060 (0.026)	0.11 [0.01, 0.23]	0.135
	480	0.053 (0.023)	0.057 (0.025)	0.12 [0.01, 0.24]	0.094
	540	0.051 (0.021)	0.056 (0.024)	0.12 [0.02, 0.25]	0.096
	600	0.049 (0.019)	0.052 (0.025)	0.13 [0.02, 0.25]	0.069
	660	0.047 (0.018)	0.051 (0.026)	0.14 [0.03, 0.25]	0.049
	720	0.046 (0.018)	0.050 (0.024)	0.18 [0.06, 0.29]	0.009
	780	0.044 (0.016)	0.049 (0.022)	0.19 [0.07, 0.30]	0.006
	840	0.043 (0.015)	0.048 (0.023)	0.19 [0.07, 0.31]	0.006
	900	0.043 (0.016)	0.049 (0.021)	0.21 [0.08, 0.32]	0.003

Table S20. Statistical results of the Mann-Whitney U test for the Degree (DA) and Mutual Information (I) of the HYPOL dataset, comparing sex-based cardiovascular differences (147 women and 129 men) using diastolic an beat-to-beat series. The table reports the median and interquartile range (IQR) for each group, the rank correlation effect size (r) with 95% confidence intervals, and FDR-corrected p-values as a function of the window size (N).

**(RR-DBP)**					
**Measure**	**Window size (N)**	**Median (IQR) Women**	**Median (IQR) Men**	**Effect Size r [95% CI]**	p_{fdr}
DA	60	8.614 (1.642)	8.833 (1.612)	0.04 [0.00, 0.15]	0.831
	120	9.048 (1.654)	9.215 (1.535)	0.05 [0.00, 0.17]	0.757
	180	9.161 (1.515)	9.310 (1.324)	0.04 [0.00, 0.16]	0.810
	240	9.298 (1.265)	9.404 (1.159)	0.07 [0.00, 0.20]	0.702
	300	9.280 (1.167)	9.472 (1.140)	0.11 [0.01, 0.23]	0.327
	360	9.254 (1.088)	9.521 (1.091)	0.13 [0.01, 0.24]	0.223
	420	9.269 (1.036)	9.543 (1.173)	0.14 [0.03, 0.25]	0.134
	480	9.292 (0.920)	9.513 (1.206)	0.15 [0.03, 0.26]	0.134
	540	9.289 (0.965)	9.509 (1.123)	0.16 [0.04, 0.27]	0.134
	600	9.398 (0.956)	9.460 (1.173)	0.14 [0.01, 0.25]	0.150
	660	9.362 (1.003)	9.525 (1.083)	0.15 [0.02, 0.25]	0.134
	720	9.382 (0.942)	9.493 (1.036)	0.15 [0.03, 0.26]	0.134
	780	9.371 (0.964)	9.506 (0.995)	0.15 [0.03, 0.26]	0.134
	840	9.299 (0.938)	9.525 (1.026)	0.16 [0.04, 0.28]	0.134
	900	9.323 (0.905)	9.547 (1.032)	0.16 [0.05, 0.28]	0.134
I	60	0.116 (0.057)	0.111 (0.062)	0.01 [0.00, 0.14]	0.906
	120	0.080 (0.036)	0.083 (0.038)	0.02 [0.00, 0.15]	0.885
	180	0.068 (0.033)	0.070 (0.026)	0.01 [0.00, 0.15]	0.906
	240	0.065 (0.027)	0.063 (0.026)	0.01 [0.00, 0.14]	0.906
	300	0.061 (0.026)	0.058 (0.021)	0.07 [0.00, 0.18]	0.702
	360	0.058 (0.026)	0.056 (0.018)	0.07 [0.00, 0.19]	0.702
	420	0.055 (0.026)	0.052 (0.017)	0.07 [0.00, 0.19]	0.702
	480	0.054 (0.023)	0.051 (0.019)	0.09 [0.01, 0.20]	0.488
	540	0.052 (0.023)	0.049 (0.018)	0.11 [0.01, 0.22]	0.381
	600	0.052 (0.024)	0.047 (0.017)	0.10 [0.01, 0.22]	0.446
	660	0.049 (0.024)	0.047 (0.017)	0.10 [0.01, 0.22]	0.446
	720	0.048 (0.023)	0.046 (0.016)	0.07 [0.00, 0.19]	0.702
	780	0.048 (0.025)	0.046 (0.015)	0.06 [0.00, 0.18]	0.702
	840	0.046 (0.024)	0.045 (0.016)	0.06 [0.00, 0.17]	0.726
	900	0.047 (0.025)	0.044 (0.016)	0.06 [0.01, 0.19]	0.702

Table S21. Statistical results of the Mann-Whitney U test for the Degree (DA) and Mutual Information (I) of the HYPOL dataset, comparing sex-based cardiovascular differences (147 women and 129 men) using systolic and diastolic series. The table reports the median and interquartile range (IQR) for each group, the rank correlation effect size (r) with 95% confidence intervals, and FDR-corrected p-values as a function of the window size (N).

		**(SBP-DBP)**			
**Measure**	**Window size (N)**	**Median (IQR) Women**	**Median (IQR) Men**	**Effect Size** r [95%CI]	$p_{\mathbf{fdr}}$
DA	60	9.258 (2.157)	9.414 (2.516)	0.05 [0.00, 0.16]	0.799
	120	10.047 (2.121)	10.189 (2.350)	0.07 [0.00, 0.19]	0.573
	180	10.338 (1.922)	10.341 (1.688)	0.08 [0.01, 0.19]	0.537
	240	10.480 (1.710)	10.524 (1.834)	0.10 [0.01, 0.22]	0.245
	300	10.402 (1.669)	10.790 (1.545)	0.17 [0.06, 0.28]	0.027
	360	10.384 (1.744)	10.850 (1.392)	0.18 [0.07, 0.30]	0.017
	420	10.432 (1.578)	10.900 (1.398)	0.20 [0.09, 0.30]	0.006
	480	10.408 (1.538)	10.995 (1.503)	0.23 [0.12, 0.35]	0.001
	540	10.418 (1.550)	11.026 (1.553)	0.23 [0.11, 0.34]	0.001
	600	10.508 (1.577)	11.051 (1.621)	0.22 [0.11, 0.33]	0.002
	660	10.492 (1.542)	11.070 (1.589)	0.23 [0.12, 0.35]	0.001
	720	10.520 (1.518)	11.156 (1.636)	0.24 [0.12, 0.35]	0.001
	780	10.512 (1.494)	11.134 (1.526)	0.25 [0.13, 0.36]	0.001
	840	10.500 (1.460)	11.207 (1.582)	0.25 [0.13, 0.36]	0.001
	900	10.537 (1.440)	11.185 (1.527)	0.26 [0.15, 0.37]	0.001
I	60	0.187 (0.172)	0.187 (0.129)	0.01 [0.00, 0.13]	0.987
	120	0.156 (0.143)	0.154 (0.130)	0.06 [0.00, 0.18]	0.734
	180	0.140 (0.113)	0.143 (0.097)	0.01 [0.00, 0.15]	0.987
	240	0.140 (0.092)	0.137 (0.091)	0.03 [0.00, 0.15]	0.913
	300	0.130 (0.086)	0.131 (0.084)	0.06 [0.00, 0.17]	0.734
	360	0.128 (0.084)	0.131 (0.081)	0.05 [0.00, 0.16]	0.784
	420	0.123 (0.079)	0.123 (0.083)	0.04 [0.00, 0.15]	0.822
	480	0.120 (0.076)	0.124 (0.082)	0.05 [0.00, 0.16]	0.799
	540	0.117 (0.069)	0.118 (0.074)	0.03 [0.00, 0.16]	0.879
	600	0.113 (0.072)	0.112 (0.073)	0.00 [0.00, 0.14]	0.987
	660	0.114 (0.068)	0.114 (0.069)	0.02 [0.00, 0.14]	0.987
	720	0.112 (0.073)	0.112 (0.064)	0.02 [0.00, 0.14]	0.987
	780	0.112 (0.068)	0.109 (0.066)	0.01 [0.00, 0.14]	0.987
	840	0.108 (0.067)	0.105 (0.067)	0.00 [0.00, 0.14]	0.987
	900	0.105 (0.069)	0.108 (0.062)	0.00 [0.00, 0.13]	0.987

Table S22. Statistical results of the Mann-Whitney U test for the Degree (DA) and Mutual Information ($\bar{I}$) of the HYPOL dataset, comparing sex-based cardiovascular differences (147 women and 129 men) using systolic, diastolic and beat-to-beat series. The table reports the median and interquartile range (IQR) for each group, the rank correlation effect size (r) with 95% confidence intervals, and FDR-corrected p-values as a function of the window size (N).

(RR-SBP-DBP)					
**Measure**	**Window size (N)**	**Median (IQR) Women**	**Median (IQR) Men**	**Effect Size r [95% CI]**	p_{fdr}
DA	60	10.000 (1.826)	10.074 (2.083)	0.04 [0.00, 0.16]	0.753
	120	10.567 (1.751)	10.703 (1.742)	0.07 [0.00, 0.20]	0.429
	180	10.728 (1.679)	10.827 (1.538)	0.08 [0.00, 0.20]	0.418
	240	10.836 (1.375)	10.965 (1.413)	0.12 [0.02, 0.23]	0.171
	300	10.800 (1.345)	11.082 (1.264)	0.17 [0.06, 0.29]	0.022
	360	10.850 (1.303)	11.212 (1.066)	0.19 [0.08, 0.31]	0.009
	420	10.828 (1.331)	11.260 (1.100)	0.21 [0.10, 0.32]	0.003
	480	10.831 (1.221)	11.224 (1.123)	0.23 [0.11, 0.35]	0.001
	540	10.842 (1.100)	11.270 (1.107)	0.24 [0.11, 0.34]	<0.001
	600	10.937 (1.168)	11.273 (1.152)	0.23 [0.12, 0.34]	0.001
	660	10.947 (1.152)	11.306 (1.130)	0.24 [0.12, 0.35]	<0.001
	720	10.959 (1.132)	11.347 (1.162)	0.25 [0.13, 0.36]	<0.001
	780	10.947 (1.132)	11.318 (1.114)	0.25 [0.13, 0.36]	<0.001
	840	10.914 (1.150)	11.351 (1.118)	0.26 [0.14, 0.37]	<0.001
	900	10.907 (1.136)	11.338 (1.198)	0.26 [0.15, 0.37]	<0.001
$\bar{I}$	60	0.149 (0.078)	0.144 (0.065)	0.01 [0.00, 0.14]	0.972
	120	0.116 (0.055)	0.114 (0.047)	0.01 [0.00, 0.13]	0.955
	180	0.099 (0.042)	0.104 (0.033)	0.04 [0.00, 0.16]	0.726
	240	0.090 (0.041)	0.096 (0.035)	0.09 [0.01, 0.21]	0.366
	300	0.086 (0.036)	0.089 (0.032)	0.09 [0.01, 0.21]	0.366
	360	0.083 (0.034)	0.087 (0.031)	0.09 [0.01, 0.20]	0.385
	420	0.079 (0.034)	0.083 (0.029)	0.08 [0.01, 0.19]	0.418
	480	0.079 (0.032)	0.081 (0.031)	0.08 [0.00, 0.20]	0.418
	540	0.075 (0.031)	0.078 (0.028)	0.07 [0.00, 0.19]	0.429
	600	0.074 (0.031)	0.076 (0.029)	0.05 [0.00, 0.16]	0.624
	660	0.073 (0.029)	0.075 (0.026)	0.07 [0.00, 0.19]	0.473
	720	0.073 (0.031)	0.074 (0.026)	0.08 [0.00, 0.20]	0.418
	780	0.071 (0.032)	0.073 (0.025)	0.07 [0.00, 0.19]	0.429
	840	0.068 (0.033)	0.070 (0.025)	0.07 [0.00, 0.19]	0.429
	900	0.067 (0.033)	0.072 (0.024)	0.08 [0.00, 0.20]	0.418

S3. Results of paired Wilcoxon test on EDA data

The proposed framework was evaluated on wearable EDA sensing data acquired during an MBSR intervention. The differences between the first and last sessions of the MBSR program were evaluated using pairwise Wilcoxon tests with FDR correction (FDR, Benjamini–Hochberg procedure, denoted as p_{FDR},$m = 4$). The proposed descriptors (DA and I) revealed an increase in meditation values from the first to the last session, considering both window lengths (60 s and 180 s). In particular:

- DA: $N = 60$, Meditation last session > Meditation first session ,$p = 6.1e-5$,$p_{FDR} = 1.22e-4$; $N = 180$ Meditation last session > Meditation first session $p = 3.05e-4$,$p_{FDR} = 6.1e-4$;
- I: $N = 60$, Meditation last session >Meditation first session $p = 6.1e-5$,$p_{FDR} = 2.44e-4$; $N = 180$ Meditation last session> Meditation first session $6.1e-5$,$p_{FDR} = 2.44e-4$;

Although this trend was consistent, not all differences between phases and sessions were equally captured by both descriptors. The difference in DA between resting state and meditation was significant in both the first and last sessions for a window length of 60 s($p = 4.27e-4$,$p_{FDR} = 5.69e-4$;$p = 6.1e-5$,$p_{FDR} = 1.22e-4$), whereas for a window length of 180 s it was significant only in the first session ($p = 6.1e-5$,$p_{FDR} = 2.44e-4$,Fig.S1). The difference in I between the first session resting state and meditation was significant only for a window length of 180 s ($p = 1.53e-3$,$p_{FDR} = 3.06e-3$,Fig.S2).

Table S23. Visibility graph DA descriptor for the Trainer–Trainee dyad (EDA-derived SMNA), comparing meditation and non-meditation conditions during the first and last session, for a window length of 60 s ($N = 15$ dyads). Values are reported as median [interquartile range]. P-values were obtained using the paired Wilcoxon signed-rank test and corrected for multiple comparisons within this table (Benjamini–Hochberg FDR, $m = 4$); asterisks indicate significance levels.

Configuration	Comparison	Median A [Q1, Q3]	Median B [Q1, Q3]	p / p_{fdr}	Sig.
Session = First	Rest vs Meditation	6.39 [4.83, 7.12]	4.69 [3.84, 5.77]	<0.001 / <0.001	***
Session = Last	Rest vs Meditation	6.64 [5.45, 7.05]	7.05 [5.97, 7.41]	<0.001 / <0.001	***
Phase = Rest	First vs Last	6.39 [4.83, 7.12]	6.64 [5.45, 7.05]	1 / 1	ns
Phase = Meditation	First vs Last	4.69 [3.84, 5.77]	7.05 [5.97, 7.41]	<0.001 / <0.001	***

Table S24. Visibility graph DA descriptor for the Trainer–Trainee dyad (EDA-derived SMNA), comparing meditation and non-meditation conditions during the first and last session, for a window length of 180 s ($N = 15$ dyads). Values are reported as median [interquartile range]. P-values were obtained using the paired Wilcoxon signed-rank test and corrected for multiple comparisons within this table (Benjamini–Hochberg FDR, $m = 4$); asterisks indicate significance levels.

Configuration	Comparison	Median A [Q1, Q3]	Median B [Q1, Q3]	p / p_{fdr}	Sig.
Session = First	Rest vs Meditation	8.50 [7.30, 10.02]	6.66 [5.76, 7.74]	<0.001 / <0.001	***
Session = Last	Rest vs Meditation	8.57 [5.97, 9.16]	8.99 [7.87, 9.09]	0.208 / 0.208	ns
Phase = Rest	First vs Last	8.50 [7.30, 10.02]	8.57 [5.97, 9.16]	0.188 / 0.208	ns
Phase = Meditation	First vs Last	6.66 [5.76, 7.74]	8.99 [7.87, 9.09]	<0.001 /<0.001	***

Table S25. Visibility graph I descriptor for the Trainer–Trainee dyad (EDA-derived SMNA), comparing meditation and non-meditation conditions during the first and last session, for a window length of 60 s ($N = 15$ dyads). Values are reported as median [interquartile range]. P-values were obtained using the paired Wilcoxon signed-rank test and corrected for multiple comparisons within this table (Benjamini–Hochberg FDR, $m = 4$); asterisks indicate significance levels.

Configuration	Comparison	Median A [Q1, Q3]	Median B [Q1, Q3]	p / p_{fdr}	Sig.
Session = First	Rest vs Meditation	7e-04 [4e-04, 1.32e-2]	4e-04 [2e-04, 1.1e-3]	0.073/ 0.146	ns
Session = Last	Rest vs Meditation	1.7 e-3[7e-04, 2.3e-3]	0.003 [1.7e-3, 0.007]	0.208/ 0.277	ns
Phase = Rest	First vs Last	7e-04 [4e-04, 1.32e-2]	1.7 e-3[7e-04, 2.3e-3]	0.33 / 0.33	ns
Phase = Meditation	First vs Last	4e-04 [2e-04, 1.1e-3]	0.003 [1.7e-3, 0.007]	<0.001 / <0.001	***

Table S26. Visibility graph I descriptor for the Trainer–Trainee dyad (EDA-derived SMNA), comparing meditation and non-meditation conditions during the first and last session, for a window length of 180 s ($N = 15$ dyads). Values are reported as median [interquartile range]. P-values were obtained using the paired Wilcoxon signed-rank test and corrected for multiple comparisons within this table (Benjamini–Hochberg FDR, $m = 4$); asterisks indicate significance levels.

Configuration	Comparison	Median A [Q1, Q3]	Median B [Q1, Q3]	p / p_{fdr}	Sig.
Session = First	Rest vs Meditation	9e-04 [3e-04, 5.1e-3]	1e-04 [1e-04, 5e-04]	<0.01 / <0.01	**
Session = Last	Rest vs Meditation	9e-04 [5e-04, 1.2e-3]	3.5e-3 [5e-04, 4.6e-3]	0.208 / 0.277	ns
Phase = Rest	First vs Last	9e-04 [3e-04, 5.1e-3]	9e-04 [5e-04, 1.2e-3]	0.421 / 0.421	ns
Phase = Meditation	First vs Last	1e-04 [1e-04, 5e-04]	3.5e-3 [5e-04, 4.6e-3]	<0.001 / <0.001	***

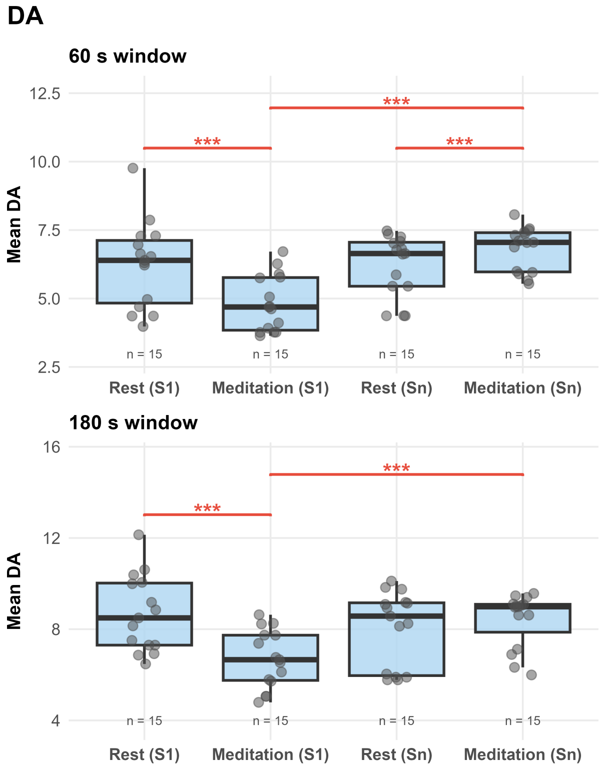

Figure S1. The boxplots represent the comparison of the DA values, resulting from the SMNA visibility graph, between rest and meditation for the first ($S1$) and last session (Sn), for both window lengths (60 s and 180 s). Window-level values were averaged by grouping by Subject, Phase, and Session, and the boxplots were computed on these averaged values. Significance thresholds are represented by $* = p < 0.05$, $** = p < 0.01$, $*** = p < 0.001$; red lines indicate significant p-values after FDR correction. Note that y-axis scales differ between the 60 s and 180 s panels and are not directly comparable.

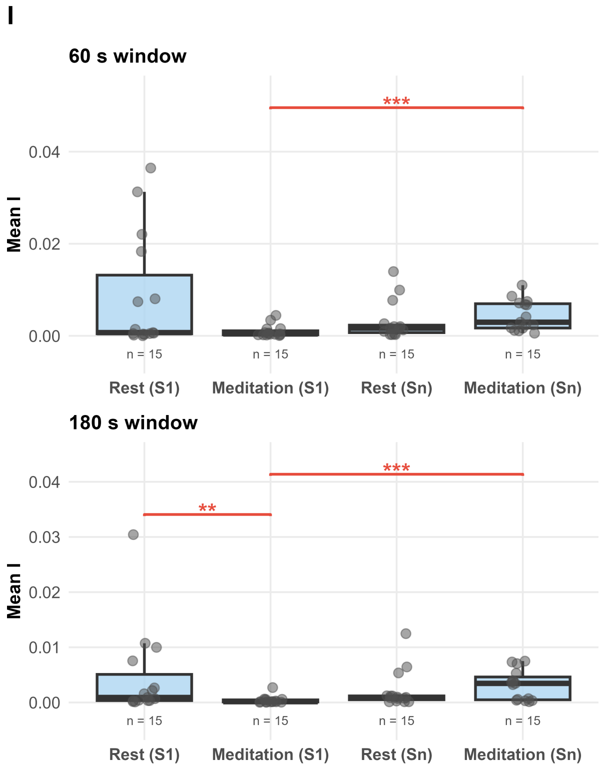

Figure S2. The boxplots represent the comparison of the I values, resulting from the SMNA visibility graph, between rest and meditation for the first (*S1*) and last session (*Sn*), for both window lengths (60 s and 180 s). Window-level values were averaged by grouping by Subject, Phase, and Session, and the boxplots were computed on these averaged values. Significance thresholds are represented by $* = p < 0.05$, $** = p < 0.01$, $*** = p < 0.001$; red lines indicate significant p-values after FDR correction. Note that y-axis scales differ between the 60 s and 180 s panels and are not directly comparable.

S3.1. Surrogates Analysis

Table S27. Statistical comparison between real dyadic interactions and the global surrogate distribution for the average degree metric (DA) across temporal windows ($N = 60\,\text{s}$ and $N = 180\,\text{s}$), with FDR correction ($m = 4$).

Window (s)	Comparison	n_{Real}	n_{Surr}	KS Stat (D)	KS p_{raw}	KS p_{FDR}	Mann–Whitney U p_{raw}	Mann–Whitney U p_{FDR}
60	Real Med (First) vs. Surr ALL	135	17,183	0.004	0.9967	0.9967 ^{ns}	1.0000	1.0000 ^{ns}
60	Real Rest (First) vs. Surr ALL	45	17,183	0.153	0.1961	0.3004 ^{ns}	0.1284	0.2568 ^{ns}
60	Real Med (Last) vs. Surr ALL	135	17,183	0.245	1.85e-8	7.41e-8 ***	5.25e-10	2.10e-9 ***
60	Real Rest (Last) vs. Surr ALL	45	17,183	0.129	0.2253	0.3004 ^{ns}	0.1983	0.2643 ^{ns}
180	Real Med (First) vs. Surr ALL	45	2,080	0.003	0.9990	0.9990 ^{ns}	0.9996	0.9996 ^{ns}
180	Real Rest (First) vs. Surr ALL	15	2,080	0.263	0.1275	0.2549 ^{ns}	0.0286	0.0572 ^{ns}
180	Real Med (Last) vs. Surr ALL	45	2,080	0.252	0.0020	0.0082 **	0.0017	0.0067 **
180	Real Rest (Last) vs. Surr ALL	15	2,080	0.219	0.2394	0.3193 ^{ns}	0.1887	0.2516 ^{ns}

Note: DA: Graph average degree, n_{Real} and n_{Surr} represent the number of observation windows. KS Stat (D) is the Kolmogorov–Smirnov test statistic (effect size). p-values are reported for one-tailed tests (Real > Surrogate). p_{FDR} denotes the Benjamini–Hochberg False Discovery Rate correction across the 4 condition comparisons per window length. Significance levels: $^{*}p_{\text{FDR}} < 0.05$, $^{**}p_{\text{FDR}} < 0.01$, $^{***}p_{\text{FDR}} < 0.001$, ^{ns}non-significant.

Table S28. Statistical comparison between real dyadic interactions and the global surrogate distribution for the mutual information metric (I) across temporal windows ($N = 60\,\text{s}$ and $N = 180\,\text{s}$), with FDR correction ($m = 4$).

Window (s)	Comparison	n_{Real}	n_{Surr}	KS Stat (D))	KS p_{raw}	KS p_{FDR}	Mann–Whitney U p_{raw}	Mann–Whitney U p_{FDR}
60	Real Med (First) vs. Surr ALL	135	17,183	0.039	0.6822	0.6822 ^{ns}	1.0000	1.0000 ^{ns}
60	Real Rest (First) vs. Surr ALL	45	17,183	0.202	0.0574	0.0766 ^{ns}	0.1635	0.2180 ^{ns}
60	Real Med (Last) vs. Surr ALL	135	17,183	0.191	2.03e-5	8.12e-5 ***	1.07e-4	4.26e-4 ***
60	Real Rest (Last) vs. Surr ALL	45	17,183	0.231	0.0084	0.0169 *	0.0097	0.0194 *
180	Real Med (First) vs. Surr ALL	45	2,080	0.005	0.9980	0.9980 ^{ns}	1.0000	1.0000 ^{ns}
180	Real Rest (First) vs. Surr ALL	15	2,080	0.312	0.0547	0.0729 ^{ns}	0.0205	0.0410 *
180	Real Med (Last) vs. Surr ALL	45	2,080	0.248	0.0025	0.0099 **	0.0012	0.0049 **
180	Real Rest (Last) vs. Surr ALL	15	2,080	0.380	0.0136	0.0271 *	0.0452	0.0602 ^{ns}

Note: I: Second-order mutual information metric. n_{Real} and n_{Surr} represent the number of observation windows. KS Stat (D) is the Kolmogorov–Smirnov test statistic (effect size). p-values are reported for one-tailed tests (Real > Surrogate). p_{FDR} denotes the Benjamini–Hochberg False Discovery Rate correction across the 4 condition comparisons per window length. Significance levels: $^{*}p_{\text{FDR}} < 0.05$, $^{**}p_{\text{FDR}} < 0.01$, $^{***}p_{\text{FDR}} < 0.001$, ^{ns}non-significant.

S3.2. Linear Mixed model

Table S29. Results of the Type II ANOVA (Satterthwaite's method) for the linear mixed-effects models fitted on DA and I computed over 60 s and 180 s aggregation w indows. M odel: $Meas_{aggregated} \sim Phase \times Session + Trainer + Sex + (1|Subject)$.

	DA **(60 s window)**			DA **(180 s window)**			I**(60 s window)**			I**(180 s window)**		
**Effect** (df_1, df_2)	F	p	η_p^2	F	p	η_p^2	F	p	η_p^2	F	p	η_p^2
Session (1,42)	22.39	< 0.001	0.35	3.98	0.053	0.09	0.37	0.547	0.009	0.04	0.848	0.001
Phase (1,42)	3.61	0.064	0.08	9.05	0.004	0.18	3.53	0.067	0.08	2.13	0.152	0.05
Trainer (1,12)	2.59	0.134	0.18	0.21	0.657	0.02	0.90	0.362	0.07	0.55	0.471	0.04
Sex (1,12)	0.65	0.436	0.05	0.18	0.682	0.01	0.04	0.852	0.003	0.10	0.760	0.008
Phase × Session (1,42)	24.57	< 0.001	0.37	19.98	< 0.001	0.32	6.99	0.011	0.14	5.72	0.021	0.12

Note. η_p^2 = partial eta squared. Marginal / conditional R^2: DA 60 s = .355/.684; DA 180 s = .215/.643; I 60 s = .173/NA; I 180 s = .134/NA. Conditional R^2 could not be computed for I models (near-zero random-intercept variance).

Table S30. Results of the post -hoc comparison for the linear mixed-effects models fitted on DA and I, computed over 60 s and 180 s aggregation windows. Model: $Meas_{aggregated} \sim Phase \times Session + Trainer + Sex + (1|Subject)$. The table reports the results for the interaction Session *time* Phase

**Model**	**Level**	**Coomparison**	**Estimate**	**SE**	**df**	t	p	**Cohen's** d
DA (N=60)	Meditation	First − Last	-1.993	0.291	42	-6.85	< 0.001	-2.50
	Rest	First − Last	0.046	0.291	42	0.16	0.874	0.06
	First	Meditation − Rest	-1.411	0.291	42	-4.85	< 0.001	-1.77
	Last	Meditation − Rest	0.629	0.291	42	2.16	0.037	0.79
DA (N=180)	Meditation	First − Last	-1.714	0.375	42	-4.57	< 0.001	-1.67
	Rest	First − Last	0.656	0.375	42	1.75	0.087	0.64
	First	Meditation − Rest	-1.983	0.375	42	-5.29	< 0.001	-1.93
	Last	Meditation − Rest	0.387	0.375	42	1.03	0.307	0.38
I(N=60)	Meditation	First − Last	-0.00341	0.00236	42	-1.44	0.157	-0.53
	Rest	First − Last	0.00544	0.00236	42	2.30	0.027	0.84
	First	Meditation − Rest	-0.00756	0.00236	42	-3.20	0.003	-1.17
	Last	Meditation − Rest	0.00128	0.00236	42	0.54	0.592	0.20
I(N=180)	Meditation	First − Last	-0.00279	0.00153	42	-1.83	0.075	-0.67
	Rest	First − Last	0.00237	0.00153	42	1.55	0.128	0.57
	First	Meditation − Rest	-0.00416	0.00153	42	-2.72	0.009	-0.99
	Last	Meditation − Rest	0.00101	0.00153	42	0.66	0.513	0.24

Disclaimer/Publisher's Note: The statements, opinions and data contained in all publications are solely those of the individual author(s) and contributor(s) and not of MDPI and/or the editor(s). MDPI and/or the editor(s) disclaim responsibility for any injury to people or property resulting from any ideas, methods, instructions or products referred to in the content.
